# Divergent Trajectories of Pediatric All-Form Tuberculosis and Multidrug-Resistant Tuberculosis from 1990 to 2021

**DOI:** 10.3390/microorganisms14071467

**Published:** 2026-07-03

**Authors:** Qing Zhang, De Chang

**Affiliations:** 1 Department of Pulmonary and Critical Care Medicine at the Seventh Medical Center, Chinese PLA General Hospital, Beijing 100700, China; zq_10084@163.com; 2 College of Pulmonary and Critical Care Medicine at the Eighth Medical Center, Chinese PLA General Hospital, Beijing 100091, China; 3 Department of Pulmonary and Critical Care Medicine, Medical School of Chinese People’s Liberation Army, Beijing 100853, China

**Keywords:** children, drug resistance, health inequality, sex disparities, mortality-to-incidence ratio

## Abstract

Using Global Burden of Disease 2021 modeled estimates, we assessed the burden, temporal trends, and inequalities of pediatric all-form tuberculosis (TB) and multidrug-resistant TB (MDR-TB) from 1990 to 2021 across 204 countries and territories. Compared with all-form TB, pediatric MDR-TB showed a distinct and less favorable estimated trajectory. Although GBD-based estimates suggested overall declines in pediatric all-form TB incidence and mortality, the MDR-to-all-form ratio increased worldwide for both incidence and mortality, suggesting a growing proportional contribution of MDR-TB within the estimated pediatric TB burden. In 2021, pediatric MDR-TB remained concentrated in low- and low–middle-SDI settings, where modeled socioeconomic inequalities appeared to become more pronounced over time. Mortality relative to incidence was highest among children aged under 5 years, with particularly elevated and imprecise mortality-to-incidence ratios for MDR-TB. Sex disparities also evolved differently by disease type: they generally narrowed for all-form TB but were more heterogeneous and in some settings widened for MDR-TB. These GBD-based findings suggest that progress in overall pediatric TB control may not have translated evenly to drug-resistant disease and highlight the need for pediatric TB strategies that explicitly address drug resistance, early childhood vulnerability, and inequitable access to diagnosis and treatment. Due to the sparsity of global pediatric data, no independent external validation was performed; findings are based on internal sensitivity analyses of GBD estimates.

## 1. Introduction

Tuberculosis (TB) remains a major cause of morbidity and mortality worldwide, and children continue to bear a substantial yet historically under-recognized share of the burden [1,2,3]. According to the Global Tuberculosis Report 2025, although global TB services have partly recovered from the disruptions caused by the COVID-19 pandemic, drug-resistant TB (DR-TB) remains a major obstacle to achieving the End TB Strategy targets [1]. Children account for an estimated 12% of the global TB burden [1,2], and untreated pediatric TB is associated with a high risk of death [3,4]. Compared with drug-susceptible disease, pediatric multidrug-resistant TB (MDR-TB) poses additional challenges because of difficulties in bacteriological confirmation, prolonged and complex treatment, and poorer clinical outcomes [3,5,6].

Recent epidemiological studies, including the GBD 2021 analysis and several updated reports on pediatric all-form TB and MDR-TB, have provided important estimates of disease burden, temporal trends, and future projections [2,7,8,9]. However, most previous studies have focused primarily on describing incidence and mortality patterns separately for all-form TB or MDR-TB [2,7,8,9]. As a result, limited attention has been paid to whether long-term progress in overall pediatric TB control has been accompanied by comparable progress in pediatric MDR-TB. Direct comparisons of the spatiotemporal trajectories of all-form TB and MDR-TB remain scarce [2,7,8,9].

This evidence gap is important because improvements in aggregate TB indicators may obscure substantial heterogeneity across disease type, age, sex, and socioeconomic context [2,3,7,8,9]. In particular, it remains unclear whether declines in the overall pediatric TB burden have been matched by similar changes in MDR-TB, or whether inequalities in age-specific mortality, sex disparities, and socioeconomic distribution have persisted or widened over time. Clarifying these patterns is essential for understanding whether current TB control gains are being shared equitably across pediatric populations.

Using the Global Burden of Disease (GBD) 2021 framework [10,11,12], this study aimed to compare the long-term epidemiological trajectories of pediatric all-form TB and MDR-TB from 1990 to 2021. Specifically, we assessed the changing contribution of MDR-TB to the overall pediatric TB burden using the MDR-to-all-form ratio (MAR) and Joinpoint regression; examined temporal changes in sex disparities using the female-to-male ratio (FMR); evaluated socioeconomic inequality using the slope index of inequality (SII) and concentration index; and further explored country-level heterogeneity in 16 high-burden countries. By integrating these dimensions within a unified analytical framework, this study seeks to move beyond descriptive reporting and provide a more comprehensive understanding of the changing burden and inequalities of pediatric TB.

## 2. Materials and Methods

### 2.1. Study Design and Data Source

This comparative time-series study used publicly available estimates from the Global Burden of Disease Study 2021 (GBD 2021), coordinated by the Institute for Health Metrics and Evaluation (IHME) (https://vizhub.healthdata.org/gbd-results/, accessed on 12 December 2025) [10,11,12,13]. GBD 2021 provides standardized and comparable estimates of health loss for 371 diseases and injuries across 204 countries and territories from 1990 to 2021 [10,11,12,13].

We extracted age-standardized and sex-specific estimates of incidence, mortality, and disability-adjusted life years (DALYs) for pediatric all-form TB and MDR-TB among HIV-negative individuals aged 0–14 years. GBD 2021 was selected to ensure consistency with the most recent comprehensive TB-specific GBD analyses [2,9]. This study was reported, where applicable, in accordance with the STROBE statement [14].

#### GBD Modeling Framework and Uncertainty

The GBD estimation process synthesizes multiple data sources, including vital registration systems, surveillance systems, surveys, and published literature, using standardized modeling frameworks to enhance cross-national comparability [10,11,12,13]. Key models include Cause of Death Ensemble modeling (CODEm) for mortality estimation and DisMod-MR 2.1 for incidence and prevalence estimation [10,11,12]. These models account for data heterogeneity, adjust for known sources of bias such as underreporting and misclassification, and propagate uncertainty throughout the estimation process [10,11,12,13]. All GBD estimates are reported with 95% uncertainty intervals (UIs), which reflect uncertainty arising from sampling error, model specification, and data sparsity [10,11,12,13]. This issue is particularly relevant for pediatric MDR-TB estimates in settings with limited primary data [6,9]. Detailed methodology is available in the GBD 2021 capstone publications [10,11,12,13].

### 2.2. Case Definitions

In accordance with the GBD 2021 framework [10,12], tuberculosis categories were defined as follows:

- All-form tuberculosis (all-form TB): all clinically diagnosed or bacteriologically confirmed tuberculosis cases caused by Mycobacterium tuberculosis, including both pulmonary and extrapulmonary disease among HIV-negative individuals [10,12]. In the present study, all-form TB represents the aggregate pediatric TB burden, including both drug-susceptible and drug-resistant forms.
- Drug-susceptible tuberculosis (DS-TB): tuberculosis that is susceptible to both isoniazid and rifampicin among HIV-negative individuals [10].
- Multidrug-resistant tuberculosis (MDR-TB): tuberculosis resistant to at least isoniazid and rifampicin, but without concurrent resistance meeting the definition of extensively drug-resistant tuberculosis (XDR-TB), among HIV-negative individuals [10].

In this study, MDR-TB was contrasted with all-form TB rather than analyzed only alongside DS-TB as a parallel subtype. This approach was adopted to assess whether temporal changes in the burden of pediatric MDR-TB paralleled those observed for the overall pediatric TB epidemic.

### 2.3. Calculation of Age-Standardized Rates (ASRs)

To enable comparisons across populations with different age structures, age-standardized rates (ASRs) per 100,000 population were calculated using the direct standardization method and the GBD 2021 standard population [10,11,13]. The GBD provides estimates for three pediatric age groups: <5, 5–9, and 10–14 years [10,11]. ASRs were calculated as:

$$\mathrm{ASR}=\frac{\sum_{i=1}^{A}(a_{i}\times w_{i})}{\sum_{i=1}^{A}w_{i}}$$

Here, a_i_ is the age-specific rate for the i-th age group, w_i_ is the corresponding standard population weight, and A is the number of age groups (three in this study). We reported the age-standardized incidence rate (ASIR), age-standardized mortality rate (ASMR), and age-standardized DALY rate (ASDR).

### 2.4. Mortality-to-Incidence Ratio (MIR)

The mortality-to-incidence ratio (MIR) was used as a descriptive population-level indicator of deaths relative to incident cases in pediatric TB. It was calculated as:

$$\mathrm{MIR}\;(\%)=\left(\frac{\mathrm{Deaths}}{\mathrm{Incident}\;\mathrm{cases}}\right)\times 100\%$$

MIR should be interpreted as an approximate population-level measure of mortality relative to incidence and is not equivalent to clinical case fatality. It may be influenced by multiple factors, including diagnostic access, treatment effectiveness, reporting completeness, survival, and GBD modeling assumptions. This caveat is particularly important for pediatric MDR-TB, for which primary mortality and incidence data are sparse in many settings. In the absence of globally comparable longitudinal pediatric cohort data, MIR was used here only as a pragmatic summary measure of mortality relative to incidence. To account for uncertainty in the GBD summary estimates, 95% UIs for MIR were approximated using the delta method on the basis of published point estimates and interval bounds. Detailed derivations and uncertainty propagation procedures are provided in the Supplementary Materials.

### 2.5. Temporal Trend Analysis and Operational Definition of Relative Divergence

#### 2.5.1. Joinpoint Regression Analysis

Temporal trends from 1990 to 2021 were analyzed using Joinpoint regression (Joinpoint Regression Program, version 5.1.0; National Cancer Institute, Bethesda, MD, USA) [15]. This method identifies time points at which the slope of a log-linear trend changes significantly. A maximum of 4 joinpoints was allowed, and the optimal model was selected using permutation tests with 4499 permutations [15]. For each outcome, the annual percentage change (APC) was estimated for each segment, and the average annual percentage change (AAPC) was calculated as a weighted average of the segment-specific APCs over the full study period, together with its 95% confidence interval (CI). A positive AAPC indicates an increasing trend, whereas a negative AAPC indicates a decreasing trend. Joinpoint regression was performed using annual GBD point estimates. Therefore, the confidence intervals and *p*-values from Joinpoint models reflect uncertainty in the fitted temporal trend based on point estimates and do not fully propagate posterior uncertainty from the GBD modeling framework. These results were interpreted as descriptive evidence of temporal patterns rather than definitive hypothesis tests.

#### 2.5.2. Calculation of the MDR-to-All-Form Ratio and Operational Classification

To provide an operational definition of the relative differences between pediatric all-form TB and MDR-TB, we calculated the MDR-to-all-form ratio (MAR). MAR was intended as a descriptive compositional indicator of the relative contribution of MDR-TB to the overall pediatric TB burden. It was not interpreted as an independent measure of MDR-TB transmission, true underlying incidence, mortality risk, or clinical severity. Furthermore, grouping results into “relative divergence,” “stability,” and “convergence” based on AAPC *p*-values was used to provide exploratory and descriptive operational labels summarizing the direction of MAR changes, rather than implying discrete biological or mechanistically defined states. MAR was calculated separately for incidence and mortality as follows:

$${\mathrm{MAR}}_{t}^{\mathrm{inc}}=\frac{\text{MDR-TB}\;{\mathrm{ASIR}}_{t}}{\text{all-form}\;\mathrm{TB}\;{\mathrm{ASIR}}_{t}}\times 100\%$$

$${\mathrm{MAR}}_{t}^{\mathrm{death}}=\frac{\text{MDR-TB}\;{\mathrm{ASMR}}_{t}}{\text{all-form}\;\mathrm{TB}\;{\mathrm{ASMR}}_{t}}\times 100\%$$

Here, t denotes calendar year and inc denotes incidence, ASIR indicates the age-standardized incidence rate, and ASMR indicates the age-standardized mortality rate.

Because MDR-TB is a subset of all-form TB, the numerator and denominator of MAR are mathematically linked, introducing compositional dependency. Therefore, an increasing MAR should be interpreted as an increase in the modeled proportional share of MDR-TB within the estimated pediatric TB burden, rather than as direct evidence of worsening MDR-TB transmission or true incidence. MAR may also be sensitive to changes in diagnostic capacity, drug-susceptibility testing coverage, surveillance completeness, and GBD modeling assumptions.

Because the publicly downloaded GBD 2021 data used in this study provided annual point estimates and corresponding 95% UIs, but not posterior draws or covariance matrices, the uncertainty of MAR was approximated from the published summary estimates. Standard errors for all-form TB and MDR-TB rates were approximated as (upper bound−lower bound)/3.92 under a normal approximation, and uncertainty for the ratio was propagated using the delta method. Because covariance information between MDR-TB and all-form TB estimates was unavailable, uncertainty propagation for MAR necessarily relied on simplifying assumptions. Accordingly, the annual 95% UIs for MAR should be interpreted as approximate rather than draw-based intervals.

Annual point estimates of MAR^inc^ and MAR^death^ were calculated in R software (version 4.5.2) and then analyzed in the Joinpoint Regression Program using the model settings described above [15]. The resulting AAPC estimates, 95% CIs, and corresponding *p*-values were used to characterize long-term changes in MAR. Because the Joinpoint analyses were conducted on annual point estimates, the AAPC confidence intervals and *p*-values reflect trend model uncertainty and do not fully propagate the underlying GBD posterior uncertainty [10,11,12,13].

#### 2.5.3. Complementary Interaction Test of Long-Term Temporal Slope Differences

As a complementary formal analysis, we fitted log-linear regression models including calendar year, disease type, and a year-by-disease-type interaction term to compare temporal trends between pediatric MDR-TB and all-form TB. To avoid undefined values during log-transformation in strata with zero or extremely low rates, a small constant (1 × 10^−9^) was added to the rates prior to analysis. The model was specified as:

$$\ln(\mathrm{Rate}\;+\;1\;\times \;10^{-9})\;=\;\beta_{0}\;+\;\beta_{1}\mathrm{Year}\;+{\;\beta }_{2}\mathrm{Type}\;+\;\beta_{3}(\mathrm{Year}\;\times \;\mathrm{Type})\;+\;\epsilon$$

Here, Rate represents the age-standardized incidence or mortality rate, Year was modeled as a continuous variable (1990–2021), and Type was coded as a binary indicator with all-form TB as the reference group. The interaction coefficient (β_3_) represents the difference in the log-linear annual slope between MDR-TB and all-form TB. A positive β_3_ indicates a more upward, or less downward, temporal trend for MDR-TB relative to all-form TB, whereas a negative β_3_ indicates a more downward temporal trend for MDR-TB relative to all-form TB. P values for the interaction term were used to test whether the long-term temporal slopes differed between the two disease categories. Ninety-five percent CIs for interaction coefficients were derived from model-based standard errors. This complementary analysis was performed at the global level, across SDI groups, and across 21 GBD regions, with results presented in Figure S2 and Table S8.

We acknowledge that annual repeated estimates may exhibit temporal autocorrelation. Because this log-linear interaction model was intended as a supplementary descriptive comparison of average long-term relative changes rather than a fully specified time-series model, its nominal *p*-values were interpreted descriptively rather than as definitive inferential evidence.

### 2.6. Female-to-Male Ratio (FMR)

Sex disparity was quantified using the female-to-male ratio (FMR), calculated as the age-standardized rate in females divided by that in males for each calendar year, location, and disease category.

$$\mathrm{FMR}=\frac{{\mathrm{Rate}}_{\mathrm{female}}}{{\mathrm{Rate}}_{\mathrm{male}}}$$

Here, Rate refers to either ASIR or ASMR.

FMRs were derived separately for ASIR and ASMR for all-form TB and MDR-TB at the global level, across SDI regions, and in selected high-burden countries during 1990–2021. An FMR of >1 indicated a higher burden in females, an FMR of <1 indicated a higher burden in males, and an FMR of =1 indicated no sex difference. Trends in FMRs over time were used to characterize changes in the magnitude and direction of sex-specific disparities.

### 2.7. Health Inequalities Analysis

Health inequalities were assessed in relation to the socio-demographic index (SDI), a composite indicator of development ranging from 0 to 1 and based on income per capita, educational attainment, and total fertility rate [10,11,13]:

- Slope index of inequality (SII): The SII was used to quantify absolute inequality [16]. Weighted least-squares regression was performed by regressing each health outcome against the relative socioeconomic rank of each country, derived from SDI and weighted by population size [16]. The SII represents the absolute difference in the predicted outcome between the hypothetically least advantaged population (rank = 0) and the most advantaged population (rank = 1) [16].
- Concentration index: The concentration index was used to quantify relative inequality [16,17]. It was derived from the concentration curve, which plots the cumulative proportion of the health outcome against the cumulative proportion of the population ranked by SDI [16,17]. A negative concentration index indicates that the outcome is disproportionately concentrated among lower-SDI populations [17]. Its value ranges from −1 to +1, with larger absolute values indicating greater relative inequality [17].

### 2.8. Statistical Analysis

All data processing, rate calculations, MIR and MAR estimations, and inequality analyses were performed in R statistical software (version 4.5.2). The complete and annotated R analysis scripts, detailing all specific packages and customized functions used, are provided as Supplementary Materials to ensure computational reproducibility. Joinpoint regression was conducted using the Joinpoint Regression Program version 5.1.0, with a maximum of four joinpoints, utilizing the Grid Search method and permutation tests with 4499 permutations for model selection and significance testing. Statistical significance was defined as a two-sided *p* < 0.05. Given the large number of subgroup analyses across SDI strata, GBD regions, sex groups, age groups, countries, and outcomes, these analyses were considered primarily descriptive and exploratory. Nominal *p*-values were not adjusted for multiplicity and should therefore be interpreted cautiously.

## 3. Results

### 3.1. Global Burden and Distribution in 2021

In 2021, there were an estimated 759,300 (95% UI: 596,056 to 949,256) incident cases and 70,659 (53,652 to 89,521) deaths from all-form TB among children globally, corresponding to an ASIR of 38.13 (26.69 to 52.60) per 100,000. For pediatric MDR-TB, the corresponding numbers were 32,515 (20,968 to 51,288) incident cases and 5887 (2467 to 11,332) deaths. Low- and low–middle-SDI regions accounted for 74.8% of global MDR-TB incidence and 92.0% of MDR-TB mortality, indicating a marked concentration of burden in less developed settings. Detailed estimates by SDI stratum and GBD region are presented in Table S1, and estimates for 204 countries and territories are provided in Table S2.

### 3.2. Age-Specific Burden and Mortality Patterns

**Figure 1:**
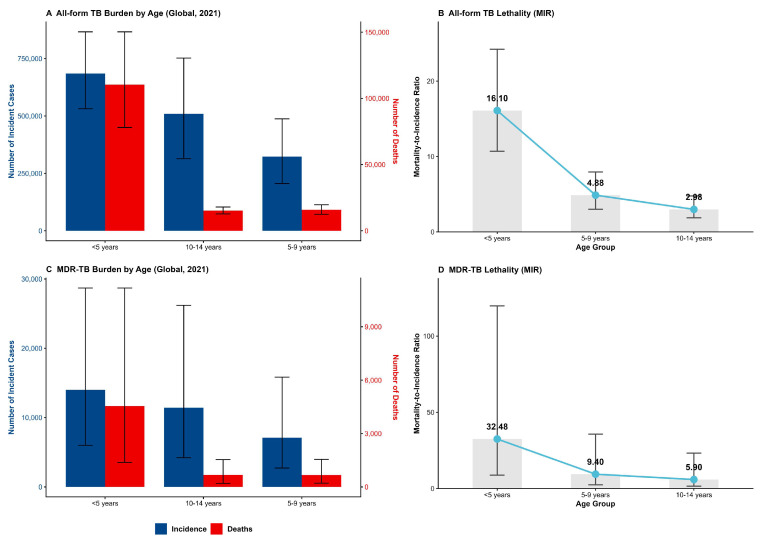
Age-stratified burden and mortality-to-incidence ratio of pediatric all-form TB and MDR-TB globally in 2021. (**A**,**C**) Absolute number of incident cases and deaths stratified by age group (<5, 5–9, and 10–14 years). (**B**,**D**) Mortality-to-incidence ratio (MIR). Panel (**D**) shows that children <5 years had an MDR-TB MIR of 32.5%, which is approximately 5.5 times that of adolescents (10–14 years).

Age-specific patterns differed between incidence and mortality in 2021. Incident cases were observed across all pediatric age groups, whereas deaths were most concentrated in children aged < 5 years for both all-form TB and MDR-TB (Figure 1A,C; Table S3). Globally, the MIR showed a marked age gradient. For all-form TB, the MIR declined from 16.10% (95% UI: 10.70 to 24.22) in children aged < 5 years to 4.88% (2.99 to 7.94) in those aged 5 to 9 years and 2.98% (1.87 to 4.77) in those aged 10 to 14 years. A similar but steeper gradient was observed for MDR-TB, with MIRs of 32.48% (8.81 to 119.79), 9.40% (2.48 to 35.63), and 5.90% (1.50 to 23.17), respectively (Figure 1B,D; Table S3).

The under-5 MIR for MDR-TB was therefore about two times that for all-form TB in the same age group and more than five times that in adolescents aged 10 to 14 years. Substantial geographic heterogeneity was also observed. Among children aged < 5 years with MDR-TB, the highest MIR estimates were seen in low-SDI regions (42.90% [16.31 to 112.83]) and in several sub-Saharan African regions, including western sub-Saharan Africa (43.18% [10.67 to 174.68]) and eastern sub-Saharan Africa (41.49% [14.12 to 121.90]) (Table S3).

### 3.3. Differences in Modeled Epidemiological Trajectories of All-Form TB and MDR-TB

**Figure 2:**
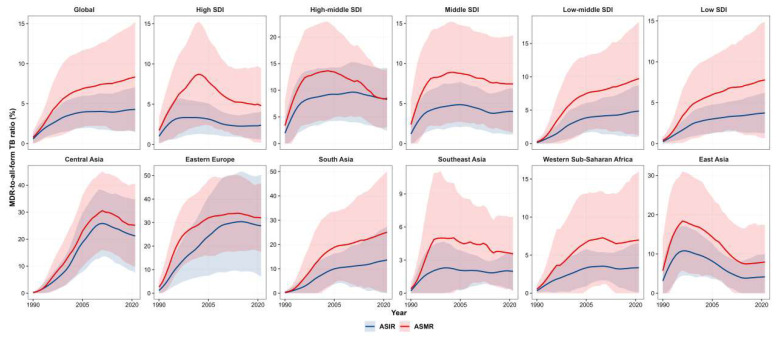
Spatiotemporal trajectories of the MDR-to-all-form ratio (MAR) in pediatric tuberculosis globally and in selected regions from 1990 to 2021. This figure presents temporal trends in the MDR-to-all-form ratio (MAR, %) for incidence-based burden (blue lines) and mortality-based burden (red lines) among children aged 0–14 years. The 12 panels show trajectories at multiple geographic levels: global; five socio-demographic index (SDI) regions; selected Global Burden of Disease (GBD) regions. These specific GBD regions were selected for visualization because they collectively encompass the areas with the highest absolute burden of pediatric MDR-TB (e.g., South Asia, Sub-Saharan Africa) and those exhibiting the most extreme relative differences (highest MAR, e.g., Eastern Europe, Central Asia). Shaded areas indicate approximate 95% uncertainty intervals (UIs) for annual MAR values, derived from published GBD point estimates and interval bounds using delta-method-based uncertainty propagation. Y-axes are presented on free scales across panels to accommodate substantial between-location heterogeneity; therefore, visual comparisons should focus primarily on within-panel temporal patterns rather than absolute magnitudes across panels.

We observed different modeled trajectories of all-form TB and MDR-TB between 1990 and 2021, reflected by a steady increase in the proportional contribution of MDR-TB, as measured by the MAR, despite declining or stable all-form TB rates (Figure 2). A fixed-axis supplementary version of these MAR trajectories, including approximate 95% uncertainty intervals, is provided in Figure S1 to facilitate comparison of absolute differences across regions and SDI strata. Globally, the incidence MAR rose from 0.57% (95% UI 0.00 to 1.15) in 1990 to 4.27% (1.51 to 7.03) in 2021, while the mortality MAR increased from 0.77% (0.00 to 1.55) to 8.32% (1.40 to 15.24). By 2021, MAR was highest in Eastern Europe (incidence: 28.63%; mortality: 32.00%) and Central Asia (21.19% and 25.15%, respectively), with similarly elevated levels in South Asia (Tables S4 and S5). Across SDI strata, the highest incidence and mortality MARs in 2021 were observed in high–middle-SDI (8.27%) and low–middle-SDI (9.73%) regions, respectively.

**Figure 3:**
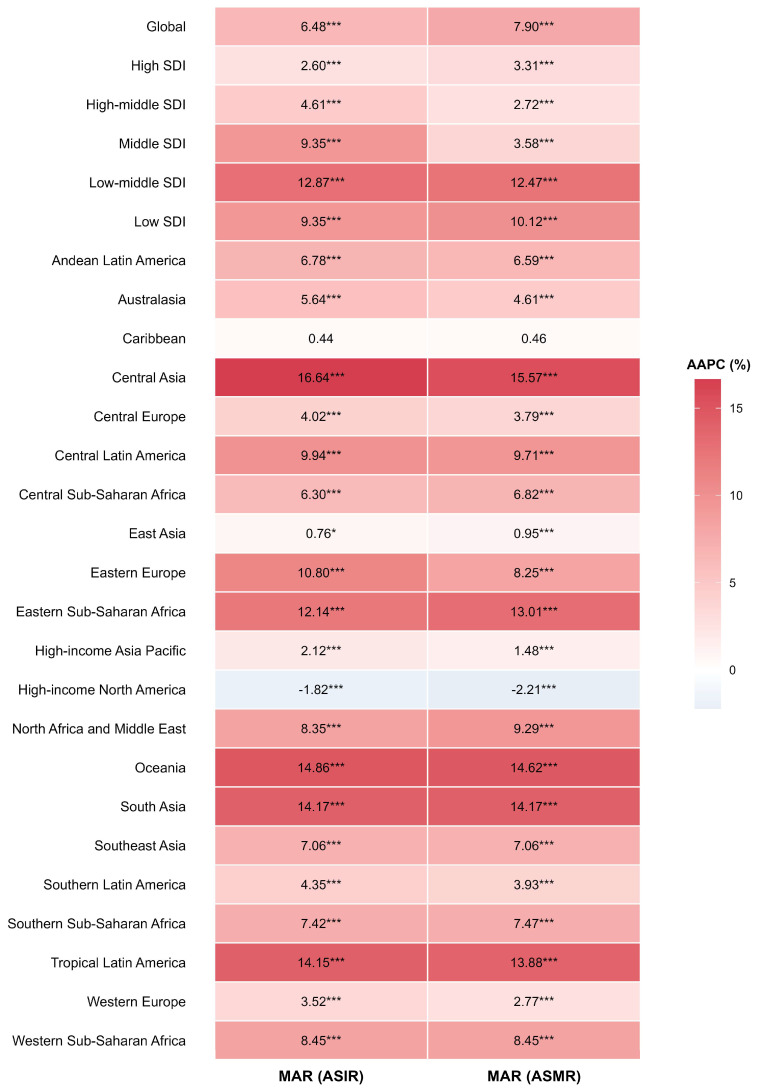
Joinpoint-based summary of temporal changes in the MDR-to-all-form ratio (MAR) from 1990 to 2021. This heatmap summarizes the AAPC in the MAR for incidence-based and mortality-based burden at the global level and across SDI and GBD regions from 1990 to 2021. The color gradient represents the direction and magnitude of the AAPC, ranging from blue (decrease) to red (increase), with white indicating values near 0. Numeric values within the cells denote AAPC point estimates. Statistical significance from Joinpoint regression models is indicated by asterisks (*** *p* < 0.001, * *p* < 0.05). Reported *p*-values are nominal, were not adjusted for multiple comparisons, and do not fully propagate GBD posterior uncertainty. Corresponding 95% CIs are provided in Supplementary Tables S6 and S7. Abbreviations: AAPC, average annual percentage change; MAR, MDR-to-all-form ratio; SDI, socio-demographic index; CI, confidence interval; GBD, Global Burden of Disease.

Joinpoint analysis of MAR further characterized these temporal changes (Figure 3; Tables S6 and S7). Globally, MAR increased notably for both incidence (AAPC: 6.48% [95% CI: 5.71 to 7.26], *p* < 0.001) and mortality (AAPC: 7.90% [7.51 to 8.29], *p* < 0.001). Regionally, the largest increases were observed in Central Asia, South Asia, Oceania, and Tropical Latin America (all AAPCs >13.0%, *p* < 0.001). At the SDI level, low–middle-SDI regions showed the most rapid increases in both incidence MAR (AAPC: 12.87% [12.35 to 13.40], *p* < 0.001) and mortality MAR (AAPC: 12.47% [11.93 to 13.02], *p* < 0.001). In contrast, high-income North America showed notable declines in both metrics (AAPCs < −1.80%). Together, these findings indicate that MDR-TB accounted for an increasing share of the overall pediatric TB burden over time, even in settings where all-form TB incidence was declining. It should be noted that the reported *p*-values in these exploratory analyses are nominal, were not adjusted for multiple comparisons, and reflect model-fit uncertainty rather than fully propagating the underlying GBD posterior uncertainty.

As a complementary formal test, we additionally fitted year-by-disease-type interaction models to assess whether long-term temporal slopes differed between MDR-TB and all-form TB (Figure S2 and Table S8). At the global level, the interaction term was statistically significant for both incidence (*β* = 0.038, *p* < 0.001) and mortality (*β* = 0.051, *p* < 0.001), indicating that the long-term log-linear temporal slopes of pediatric MDR-TB differed from those of all-form TB over 1990–2021. Marked geographic heterogeneity was observed. The largest positive interaction coefficients for incidence were identified in low–middle-SDI (*β* = 0.077, *p* < 0.001) and low-SDI regions (*β* = 0.057, *p* < 0.001), indicating a more upward, or less downward, incidence trend for MDR-TB relative to all-form TB in these settings. By contrast, interaction terms were not statistically significant in high-SDI regions for either incidence or mortality (*p* > 0.05), suggesting more similar long-term temporal slopes between the two disease categories. Overall, these complementary interaction analyses were broadly consistent with the MAR-based findings at the global level and across many high-burden settings.

To evaluate the robustness of the observed relative divergence patterns, we conducted several sensitivity analyses. First, because the widespread rollout of rapid molecular diagnostics primarily occurred in the latter half of the study period, we restricted the temporal trend analysis of MAR to the 2000–2021 period. The increasing trends in MAR remained highly significant globally and across lower-SDI regions during this restricted timeframe (Table S15). Second, to address potential mathematical artifacts introduced by age-standardization procedures, we recalculated MAR using absolute incidence and mortality counts (count-based MAR). This alternative definition yielded consistent temporal slopes, confirming the increasing proportional share of MDR-TB over time (Table S16). Finally, sensitivity analyses confirmed that the year-by-disease-type interaction models were robust to the selection of alternative log-transformation constants (10^−3^, 10^−6^, and 10^−9^) for handling near-zero rates in specific strata (Table S17).

### 3.4. Temporal Changes in Sex Disparities

**Figure 4:**
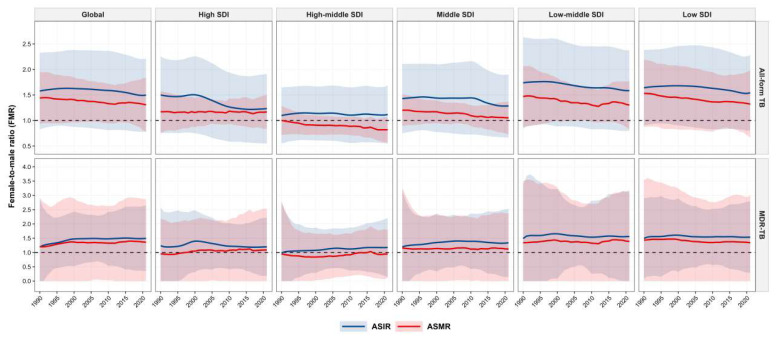
Temporal trends in sex disparities (female-to-male ratio) for pediatric all-form TB and MDR-TB globally and across SDI regions from 1990 to 2021. This figure presents the temporal trends of sex disparities, quantified using the female-to-male ratio (FMR) of age-standardized rates among HIV-negative children aged 0–14 years. Columns display data at the global level and across the five Socio-demographic Index (SDI) regions. The first row presents the FMRs for pediatric all-form TB, and the second row presents the FMRs for MDR-TB. Within each panel, blue lines represent the FMR of the age-standardized incidence rate (ASIR), and red lines represent the FMR of the age-standardized mortality rate (ASMR). Solid lines indicate annual point estimates, while shaded areas represent approximate 95% uncertainty intervals (UIs) derived via delta-method-based uncertainty propagation. The horizontal dashed line at 1.0 denotes sex parity; an FMR > 1 indicates a higher burden in females, whereas an FMR < 1 indicates a higher burden in males.

Sex disparities showed distinct temporal patterns for all-form TB and MDR-TB (Figure 4 and Tables S9–S11). Globally, the point estimates of the female-to-male ratio (FMR) for all-form TB remained above 1 for both incidence and mortality throughout 1990–2021, although both measures declined modestly over time. The FMR for ASIR decreased from 1.58 (95% UI: 0.83 to 2.33) in 1990 to 1.50 (0.78 to 2.21) in 2021, while the FMR for ASMR declined from 1.44 (0.93 to 1.95) to 1.31 (0.77 to 1.84) (Table S9). Joinpoint analysis indicated an overall narrowing of sex disparities in all-form TB, with negative AAPCs for both incidence (−0.18% [−0.21 to −0.15], *p* < 0.001) and mortality (−0.32% [−0.41 to −0.23], *p* < 0.001) (Table S10). This decline was not entirely monotonic: the incidence FMR increased slightly during 1990–1996 before declining, whereas the mortality FMR showed a brief rebound during 2011–2016 amid its overall downward trend. Across SDI strata, incidence disparities narrowed in all but high–middle-SDI regions, where they remained stable; similarly, mortality disparities declined across all strata except for high SDI.

For MDR-TB, the global point estimates of FMR also remained above 1, but their temporal trajectories contrasted with those of all-form TB. The FMR for ASIR increased from 1.19 (0.00 to 2.85) in 1990 to 1.49 (0.34 to 2.65) in 2021, and the FMR for ASMR rose from 1.20 (0.00 to 2.91) to 1.36 (0.00 to 2.87). Consistent with this pattern, global AAPCs were positive for both incidence (0.65% [0.60 to 0.70], *p* < 0.001) and mortality (0.42% [0.29 to 0.55], *p* < 0.001), indicating widening sex disparities over time (Table S11). At the SDI level, MDR-TB incidence disparities showed a notable increase in high-middle (AAPC: 0.44% [0.31 to 0.56], *p* < 0.001) and middle SDI regions (0.29% [0.21 to 0.37], *p* < 0.001), while remaining stable elsewhere. Mortality disparities showed greater heterogeneity, increasing in high-SDI regions (0.39% [0.08 to 0.70], *p* = 0.015), declining in low-SDI regions (−0.22% [−0.30 to −0.13], *p* < 0.001), and remaining stable in other strata. Overall, these findings indicate that sex disparities in all-form TB gradually narrowed, whereas those in MDR-TB were more heterogeneous and widened in several settings.

At the national level, data from the 16 high-burden countries further illustrated the divergence between all-form TB and MDR-TB (Tables S12–S14). For all-form TB, sex disparities showed a notable narrowing in most of these countries, with predominantly negative AAPCs observed for both incidence and mortality (Figure S3 and Table S13). By contrast, temporal trends in MDR-TB FMR were more heterogeneous (Figure S4 and Table S14). Although a few countries showed modest reductions in sex disparities, several others exhibited stable or increasing trends, reflected by non-significant or positive AAPCs. By 2021, the point estimates of MDR-TB incidence FMR remained above 1 in almost all 16 countries (Table S12). Together, these national-level findings indicate substantial heterogeneity in MDR-TB sex disparities across high-burden countries, while female predominance in incidence persisted in most settings.

### 3.5. Socioeconomic Inequalities in TB Burden

**Figure 5:**
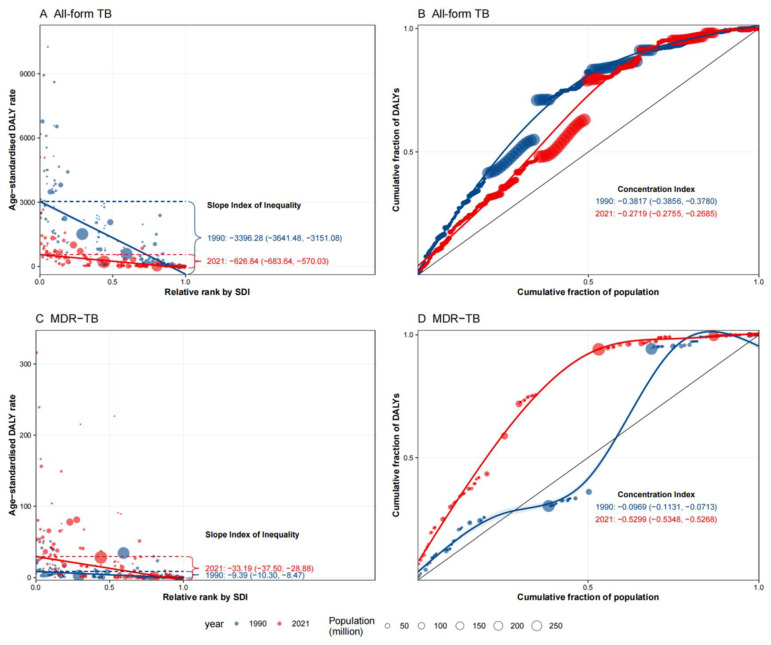
Diverging trends in socioeconomic inequalities for pediatric all-form TB and MDR-TB from 1990 to 2021. Analysis of socioeconomic inequality evolution based on the socio-demographic index (SDI). (**A**,**C**) Slope index of inequality (SII) analysis, representing absolute inequality. The absolute gap for all-form TB narrowed between 1990 and 2021 (**A**), whereas absolute inequality for MDR-TB widened, as evidenced by the SII decreasing from −9.39 in 1990 to −33.19 in 2021 (**C**). (**B**,**D**) Concentration index analysis (Lorenz curves), representing relative inequality. All-form TB inequality showed a converging trend (**B**), whereas MDR-TB relative inequality intensified over the three decades, reaching −0.53 in 2021 (**D**). A negative SII or concentration index indicates that the disease burden is disproportionately concentrated among populations with lower SDI levels.

Marked socioeconomic inequalities were observed in the distribution of pediatric TB burden across SDI levels, with contrasting patterns between all-form TB and MDR-TB (Figure 5). For all-form TB, absolute inequality in ASDRs decreased substantially over time, with the SII narrowing from −3396.28 (95% CI: −3641.48 to −3151.08) in 1990 to −626.84 (−683.64 to −570.03) in 2021. Relative inequality also improved, as the concentration index changed from −0.3817 (−0.3856 to −0.3780) in 1990 to −0.2719 (−0.2755 to −0.2685) in 2021.

In contrast, inequality in the pediatric MDR-TB burden worsened over the same period. The SII became more negative, increasing in magnitude from −9.39 (−10.30 to −8.47) in 1990 to −33.19 (−37.50 to −28.88) in 2021, indicating a widening absolute gap in ASDRs between lower- and higher-SDI populations. Relative inequality showed a similar pattern: the concentration index shifted from −0.0969 (−0.1131 to −0.0713) in 1990 to −0.5299 (−0.5348 to −0.5268) in 2021.

## 4. Discussion

This study provides an updated global assessment of the burden, temporal trends, and inequalities of pediatric TB and MDR-TB from 1990 to 2021 using GBD 2021 modeled estimates. Several findings merit emphasis. First, the estimated temporal trajectories of all-form TB and MDR-TB appeared to diverge over the past three decades. While the modeled burden of all-form TB generally declined, MDR-TB represented an increasing proportional share of the estimated overall pediatric TB burden. Second, pediatric MDR-TB remained disproportionately concentrated in low- and low–middle-SDI settings, suggesting persistent socioeconomic inequality in modeled burden. Third, marked age- and sex-related heterogeneity was observed: mortality relative to incidence was highest among children aged < 5 years, and sex disparities evolved differently for all-form TB and MDR-TB. Together, these GBD-based findings suggest that progress in overall pediatric TB control may not have translated to drug-resistant disease evenly. It is important to note that no independent external validation of these GBD-modeled estimates was performed, as globally comparable independent surveillance datasets for pediatric MDR-TB are currently lacking. Instead, we relied on rigorous internal sensitivity analyses and robustness checks.

The differing modeled trajectories of all-form TB and MDR-TB likely reflect differences in the complexity of prevention, diagnosis, and treatment [1,3,6]. Declines in all-form TB are broadly consistent with improvements in living conditions, expansion of routine TB services, increased access to first-line treatment, and, for severe pediatric TB outcomes, sustained BCG vaccination coverage [1,2,6]. By contrast, effective control of pediatric MDR-TB depends not only on general TB control, but also on early detection of drug resistance, access to bacteriological confirmation, timely drug-susceptibility testing, availability of second-line regimens, and interruption of transmission from infectious adult-source cases [3,4,5,6,18,19]. These additional requirements may partly explain why progress against pediatric MDR-TB has been slower, less stable, and more heterogeneous than that observed for all-form TB [3,4,5,6].

Importantly, the observed increase in MAR must be interpreted cautiously. Because MDR-TB is a subset of all-form TB, the numerator and denominator are mathematically linked, introducing compositional dependency. MAR should therefore be viewed primarily as a descriptive, surveillance-sensitive indicator rather than as independent inferential evidence of MDR-TB transmission or true incidence. Because the published GBD summary estimates do not provide covariance information between all-form TB and MDR-TB estimates, uncertainty propagation for MAR necessarily relied on simplifying assumptions. Furthermore, an increasing MAR does not exclusively indicate a true epidemiological worsening of MDR-TB burden. The widespread adoption of rapid molecular diagnostics, such as GeneXpert MTB/RIF, particularly after 2010, has substantially improved the detection of rifampicin resistance globally [20,21]. Therefore, the observed difference in trajectories likely reflects a complex combination of true epidemiological changes, improved detection of drug resistance, evolving surveillance systems, and GBD modeling assumptions. Part of the increasing MAR observed in recent years may represent an apparent increase due to this improved detection capacity rather than solely a true upward shift in MDR-TB epidemiology.

Our findings also highlight pronounced age-related vulnerability, particularly among children aged <5 years. Although incident cases occurred across all pediatric age groups, deaths were concentrated in the youngest children, and MIR estimates were higher for MDR-TB than for all-form TB. However, it is crucial to note that MIR in GBD-modeled datasets should be interpreted as an approximate population-level measure of mortality relative to incidence and is not equivalent to clinical case fatality. The wide uncertainty intervals observed, particularly for MDR-TB in children aged under 5 years, limit definitive inferences. This pattern is nevertheless biologically and programmatically plausible [3,4,6]. Young children are more vulnerable to rapid disease progression following infection, are less able to produce respiratory specimens, and often present with paucibacillary or non-specific disease, all of which complicate diagnosis and delay treatment initiation [3,4,6,19]. The especially high MIRs observed in low-SDI and sub-Saharan African settings may suggest that age-related vulnerability is compounded by health-system constraints, limited access to diagnostics, and broader contextual disadvantage [3,6,19], although these findings should be framed as hypotheses rather than direct empirical observations.

The concentration of pediatric MDR-TB burden in lower-SDI settings underscores the continuing role of structural inequality [6,22,23]. Children living in these settings are more likely to face delayed diagnosis, restricted access to drug-susceptibility testing, treatment interruption, undernutrition, and sustained household exposure to untreated or inadequately treated adult TB [6,18,22,23]. For MDR-TB in particular, weaknesses in laboratory capacity, referral systems, and access to child-friendly second-line treatment may contribute to both under-detection and poor outcomes [5,6,19]. The widening socioeconomic inequality observed for pediatric MDR-TB, in contrast to the improving inequality pattern for all-form TB, may suggest that current gains in childhood TB control have been uneven and that disadvantaged populations may have benefited less from advances in diagnosis and treatment of drug-resistant disease [6,22,23].

Sex disparities represented another important dimension of heterogeneity. Globally, the point estimates of the female-to-male ratio remained above 1 for both incidence and mortality, but temporal patterns differed by disease type. For all-form TB, sex disparities gradually narrowed over time, as indicated by declining FMRs and negative AAPCs. In contrast, for MDR-TB, global FMRs increased modestly and AAPCs were positive for both incidence and mortality, suggesting widening disparities overall. Country-level analyses of 16 high-burden countries further reinforced this difference: most countries showed narrowing sex disparities for all-form TB, whereas patterns for MDR-TB were considerably more heterogeneous, with stable or increasing disparities in several settings. These findings suggest that sex differences in pediatric TB are context-specific and may differ fundamentally from adult TB epidemiology. Unlike adult TB, where males typically exhibit a higher burden partly driven by occupational exposure, smoking, alcohol use, and other behavioral risk factors [24,25], GBD-based pediatric estimates in this study showed a slightly higher burden among females globally. This unexpected female predominance, together with the more heterogeneous and widening disparities observed for MDR-TB, warrants further investigation. These patterns may reflect a complex interplay of sex-specific care-seeking patterns, household decision-making, differential access to diagnostic evaluation and treatment, and potential notification biases [24,26]. Furthermore, age-dependent immunological differences during early childhood and adolescence may contribute to these patterns, although sparse pediatric data and GBD modeling assumptions may also influence sex-specific estimates [24,27]. Importantly, the less favorable patterns observed for MDR-TB imply that gains in sex equity may not have been shared equally across disease types.

The country-level findings also have practical implications. Although global and SDI-level analyses are useful for identifying broad patterns, the substantial heterogeneity across the 16 high-burden countries indicates that national responses cannot rely on aggregate assumptions alone. In some countries, sex disparities in MDR-TB remained persistent despite narrowing disparities in all-form TB, suggesting that routine TB control gains do not necessarily extend to drug-resistant disease. This reinforces the need for country-specific strategies that strengthen pediatric contact investigation, improve access to rapid molecular diagnostics, expand decentralized drug-susceptibility testing, and ensure timely initiation of appropriate treatment for children exposed to or affected by MDR-TB [6,18,19].

Taken together, these findings have several policy implications. First, declines in all-form TB should not be interpreted as evidence of equivalent progress against pediatric MDR-TB. Second, reducing the burden of pediatric MDR-TB will likely require stronger integration of child contact management, rapid diagnosis, resistance testing, and access to effective second-line regimens within routine TB services [3,4,5,6,19]. Third, the persistence of socioeconomic and sex-related inequalities suggests that biomedical interventions alone are insufficient; equitable progress will also depend on improving access to care among children living in disadvantaged households and health systems [22,23]. A more targeted pediatric TB control strategy is therefore needed—one that addresses not only overall burden reduction, but also drug resistance, age-specific vulnerability, and unequal access to diagnosis and treatment [1,3,6].

This study has several limitations. First, it was based on GBD 2021 modeled estimates and therefore inherits the assumptions and uncertainties of the GBD modeling framework [10,11,12,13]. In settings with sparse surveillance data, weak vital registration systems, or limited microbiological confirmation, the true burden may be under- or over-estimated [10,11,12,13]. This issue is particularly important for pediatric MDR-TB, for which primary data are often limited. Additionally, because the publicly available GBD summary dataset does not provide a standardized country-level data-quality flag or posterior draw structure, we were unable to formally exclude low-data-quality countries in our sensitivity analyses. As with many macro-epidemiological analyses, no independent external validation was performed, and our evaluations were strictly internal robustness checks. Second, this was an ecological analysis; therefore, the observed differences by age, sex, country, and SDI should not be interpreted as evidence of individual-level causation. Third, pediatric TB, particularly pediatric MDR-TB, remains underdiagnosed in many settings because of non-specific clinical presentation and the difficulty of obtaining bacteriological confirmation in children [3,4,19]. Changes in diagnostic capacity, including the expanded use of rapid molecular tests such as GeneXpert MTB/RIF [20,21], may affect both level estimates and temporal trends. Fourth, MAR is a descriptive compositional indicator rather than an independent epidemiological measure. Because MDR-TB is a subset of all-form TB, the numerator and denominator are mathematically linked, and the resulting ratios should be interpreted as modeled proportional shares rather than direct evidence of MDR-TB transmission or true incidence. Fifth, uncertainty intervals for derived indicators such as MAR, MIR, and FMR were approximated from published GBD summary estimates and interval bounds. Because posterior draws and covariance information were not available in the publicly downloaded dataset, these intervals do not fully propagate GBD posterior uncertainty and should be regarded as approximate. Sixth, uncertainty intervals for several MDR-TB indicators—especially age- or sex-stratified estimates at the country level—were wide, and point estimates should therefore be interpreted cautiously. Seventh, given the large number of subgroup analyses performed across SDI strata, GBD regions, sex groups, age groups, countries, and outcomes, the subgroup findings should be regarded as descriptive and exploratory. Nominal *p*-values were not adjusted for multiplicity and should therefore be interpreted cautiously. Finally, APC and AAPC derived from Joinpoint regression summarize temporal change but do not identify the underlying clinical, social, diagnostic, or programmatic mechanisms responsible for these patterns [15].

## 5. Conclusions

From 1990 to 2021, GBD 2021-modeled estimates suggested different modeled temporal trajectories between pediatric all-form TB and MDR-TB worldwide, with slower and more heterogeneous estimated progress for MDR-TB than for all-form TB. Pediatric MDR-TB remained concentrated in lower-SDI settings, mortality relative to incidence was highest among children aged < 5 years, and sex disparities evolved differently by disease type: they generally narrowed for all-form TB but were more heterogeneous and in some settings widened for MDR-TB. These findings should be interpreted in light of GBD modeling uncertainty, diagnostic changes, and the compositional nature of MAR. Nevertheless, they highlight the need for pediatric TB strategies that go beyond overall burden reduction and explicitly address drug resistance, early childhood vulnerability, and inequitable access to diagnosis and treatment.

## Data Availability

The GBD 2021 data are openly available at the Global Health Data Exchange (GHDx) platform (https://vizhub.healthdata.org/gbd-results/, accessed on 12 December 2025). The annotated R analysis scripts used for data processing, delta-method approximations, and interaction modeling in this study are provided as Supplementary Materials to enhance computational reproducibility.

## Supplementary Materials

The following supporting information can be downloaded at: https://www.mdpi.com/article/10.3390/microorganisms14071467/s1, Supplementary Methodology: Derivations and uncertainty propagation procedures; R codes: Complete and annotated R analysis scripts for data processing, rate calculations, and statistical modeling; Figure S1: Fixed-axis spatiotemporal trajectories of the MDR-to-all-form ratio (MAR) in pediatric tuberculosis globally and in selected regions, 1990–2021; Figure S2: Complementary interaction test of long-term temporal slope differences; Figure S3: Temporal trends in sex disparities (female-to-male ratio) for pediatric all-form TB across 16 high-burden countries; Figure S4: Temporal trends in sex disparities (female-to-male ratio) for pediatric MDR-TB across 16 high-burden countries; Table S1: Detailed estimates of pediatric all-form TB and MDR-TB by SDI stratum and GBD region in 2021; Table S2: Detailed estimates of pediatric all-form TB and MDR-TB for 204 countries and territories in 2021; Table S3: Age-specific burden and mortality-to-incidence ratio of pediatric all-form TB and MDR-TB globally and by region in 2021; Table S4: Incidence MDR-to-all-form ratio (MAR) globally, by SDI, and GBD region; Table S5: Mortality MDR-to-all-form ratio (MAR) globally, by SDI, and GBD region; Table S6: Joinpoint regression analysis of incidence MAR globally, by SDI, and GBD region; Table S7: Joinpoint regression analysis of mortality MAR globally, by SDI, and GBD region; Table S8: Complementary interaction test of long-term temporal slope differences between MDR-TB and all-form TB; Table S9: Temporal trends in female-to-male ratio (FMR) for pediatric all-form TB and MDR-TB globally and by SDI region; Table S10: Joinpoint regression analysis of FMR for pediatric all-form TB globally and by SDI region; Table S11: Joinpoint regression analysis of FMR for pediatric MDR-TB globally and by SDI region; Table S12: Female-to-male ratio point estimates for all-form TB and MDR-TB in 16 high-burden countries; Table S13: Joinpoint regression analysis of FMR for pediatric all-form TB in 16 high-burden countries; Table S14: Joinpoint regression analysis of FMR for pediatric MDR-TB in 16 high-burden countries; Table S15: Sensitivity analysis of temporal trends in the multidrug-resistant tuberculosis to all-form tuberculosis ratio (MAR) among children (0–14 years), globally and by Socio-demographic Index (SDI), restricted to the 2000–2021 period; Table S16: Sensitivity analysis of temporal trends in the multidrug-resistant tuberculosis to all-form tuberculosis ratio (MAR) among children (0–14 years), using count-based absolute cases and deaths as an alternative definition, 1990–2021; Table S17: Sensitivity analysis of the interaction coefficient (β) assessing the temporal divergence between multidrug-resistant tuberculosis (MDR-TB) and all-form tuberculosis incidence and mortality among children (0–14 years), globally and by SDI, 1990–2021, using different arbitrary log-transformation constants.
