# Supplementary material for: Divergent Trajectories of Pediatric All-Form Tuberculosis and Multidrug-Resistant Tuberculosis from 1990 to 2021
Source: Microorganisms. 2026 Jul 3;14(7):1467. doi: 10.3390/microorganisms14071467 (PMC13414464; doi:10.3390/microorganisms14071467)
Supplement: Supplementary file 1 [file microorganisms-14-01467-s001.zip › Supplementary Materials.pdf]

Supplementary Methodology

For each GBD point estimate, the standard error (SE) was approximated from the corresponding 95% uncertainty interval (UI) under a normal approximation on the linear scale:

$$SE_X = \frac{UI_{upper,X} - UI_{lower,X}}{3.92}$$

$$SE_Y = \frac{UI_{upper,Y} - UI_{lower,Y}}{3.92}$$

where X denotes the numerator estimate and Y denotes the denominator estimate.

For a ratio-based indicator, $R = \frac{X}{Y}$, the variance of R was approximated using the first-order delta method:

$$Var(R) \approx R^2 \left(\frac{Var(X)}{X^2} + \frac{Var(Y)}{Y^2} - 2\frac{Cov(X,Y)}{XY} \right)$$

Because posterior draws and covariance matrices were not available in the publicly downloaded GBD 2021 summary data, covariance terms could not be directly incorporated. Therefore, we assumed $Cov(X, Y) = 0$, yielding the following approximation:

$$SE_R \approx R \sqrt{\left(\frac{SE_X}{X}\right)^2 + \left(\frac{SE_Y}{Y}\right)^2}$$

The approximate 95% uncertainty interval for R was then calculated as: $95\%UI_R = R \pm 1.96 \times SE_R$

This procedure was applied to ratio-based indicators, including the MDR-to-all-form ratio (MAR), mortality-to-incidence ratio (MIR), and female-to-male ratio (FMR), where applicable. Because MDR-TB is nested within all-form TB, the true covariance between the numerator and denominator for MAR is likely to be positive. In that case, assuming zero covariance may overestimate the variance of the ratio. However,

because the covariance structure of the GBD-modeled estimates was unavailable, the direction and magnitude of this approximation could not be fully verified. Therefore, all delta-method-derived uncertainty intervals should be interpreted as approximate intervals rather than full posterior credible intervals from GBD draws.

We used a normal approximation on the linear scale because the publicly available GBD outputs provide point estimates and 95% uncertainty bounds, but not posterior draws. This approach allows transparent reconstruction of approximate standard errors from published summary intervals. We acknowledge that log-normal approximations may be preferable for strictly positive and highly skewed quantities, particularly when uncertainty intervals are asymmetric. Therefore, the derived intervals were interpreted as approximate rather than exact posterior intervals.

Supplementary Figures

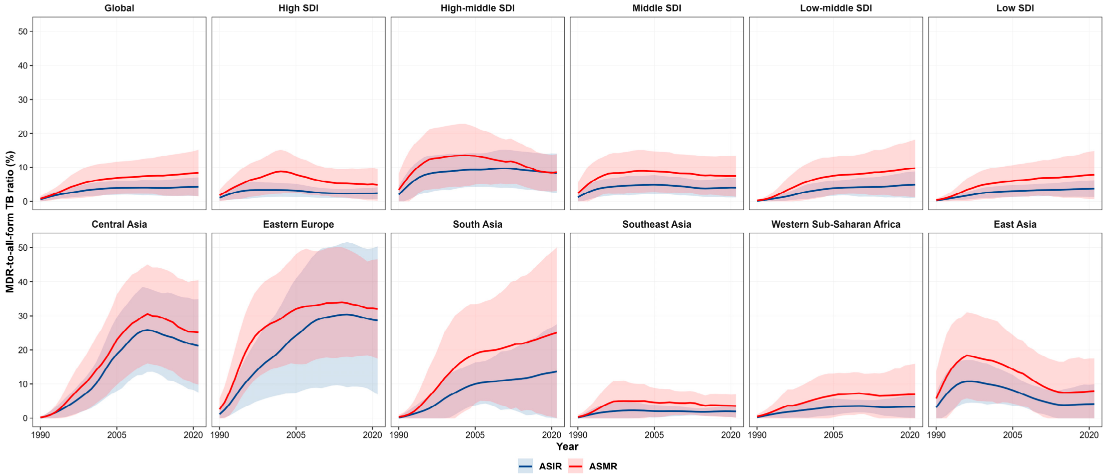

Figure S1. Fixed-axis spatiotemporal trajectories of the MDR-to-all-form ratio (MAR) in pediatric tuberculosis globally and in selected regions, 1990–2021. This figure presents the identical temporal trends in the MDR-to-all-form ratio (MAR, %) as shown in the main text (Figure 2), but utilizes a fixed (harmonized) Y-axis scale across all 12 panels. This harmonized scaling is provided to facilitate direct cross-panel visual comparisons of the absolute magnitudes of MAR between different regions. Blue lines represent the MAR for incidence-based burden, and red lines represent the MAR for mortality-based burden among HIV-negative children aged 0–14 years. Shaded areas indicate approximate 95% uncertainty intervals (UIs) for annual MAR values, derived from published GBD point estimates and interval bounds using delta-method-based uncertainty propagation, rather than full posterior credible intervals from GBD draws.

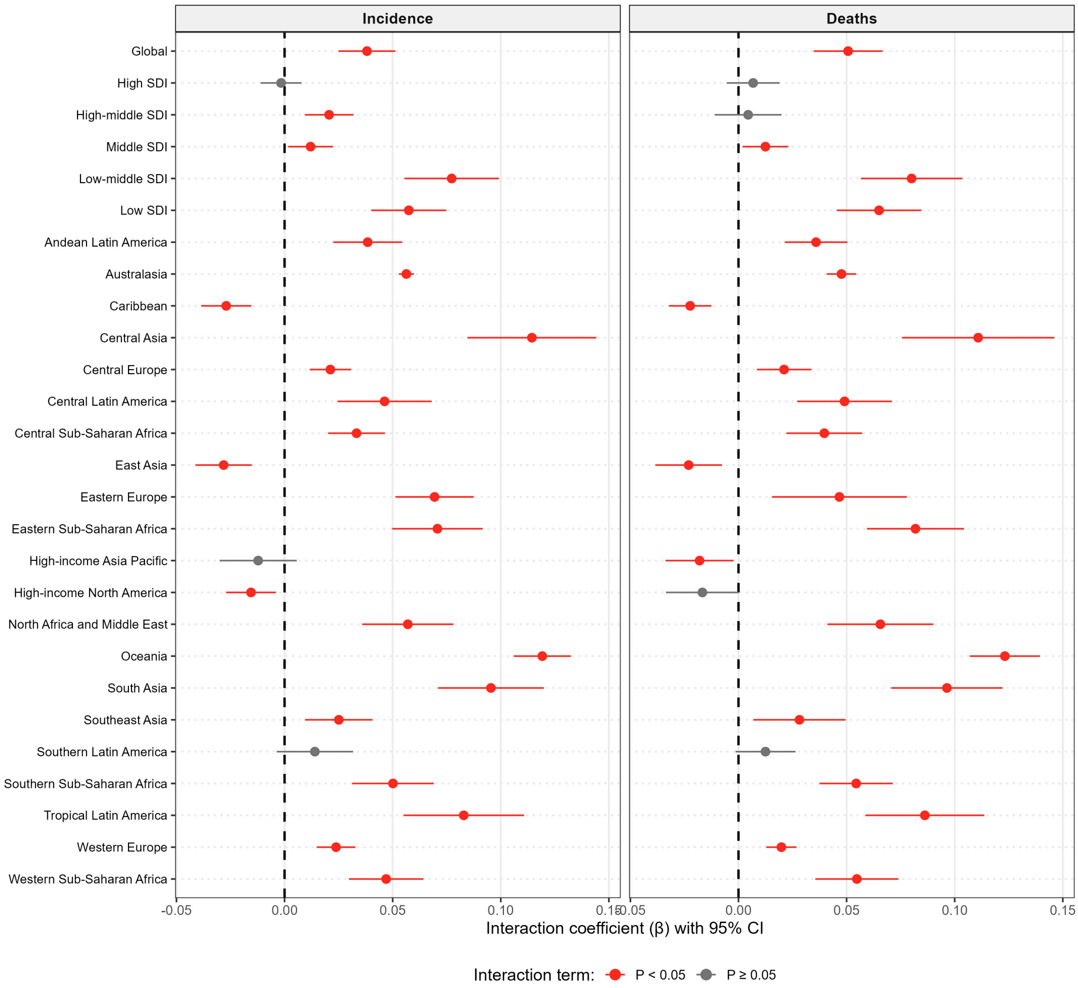

Figure S2. Forest plot of year-by-disease-type interaction coefficients for pediatric tuberculosis incidence and mortality across global and regional settings, 1990–2021. Interaction coefficients (β) were estimated from log-linear regression models including calendar year, disease type, and a year-by-disease-type interaction term, with all-form tuberculosis (TB) as the reference group. The coefficient (β) represents the difference in the log-linear annual slope between multidrug-resistant tuberculosis (MDR-TB) and all-form TB. Positive coefficients indicate a more upward, or less downward, temporal trend for MDR-TB relative to all-form TB, whereas negative coefficients indicate a more downward temporal trend for MDR-TB relative to all-form TB. Red symbols indicate interaction terms with $P < 0.05$; grey symbols

indicate P ≥ 0.05. Error bars represent 95% confidence intervals (CIs).

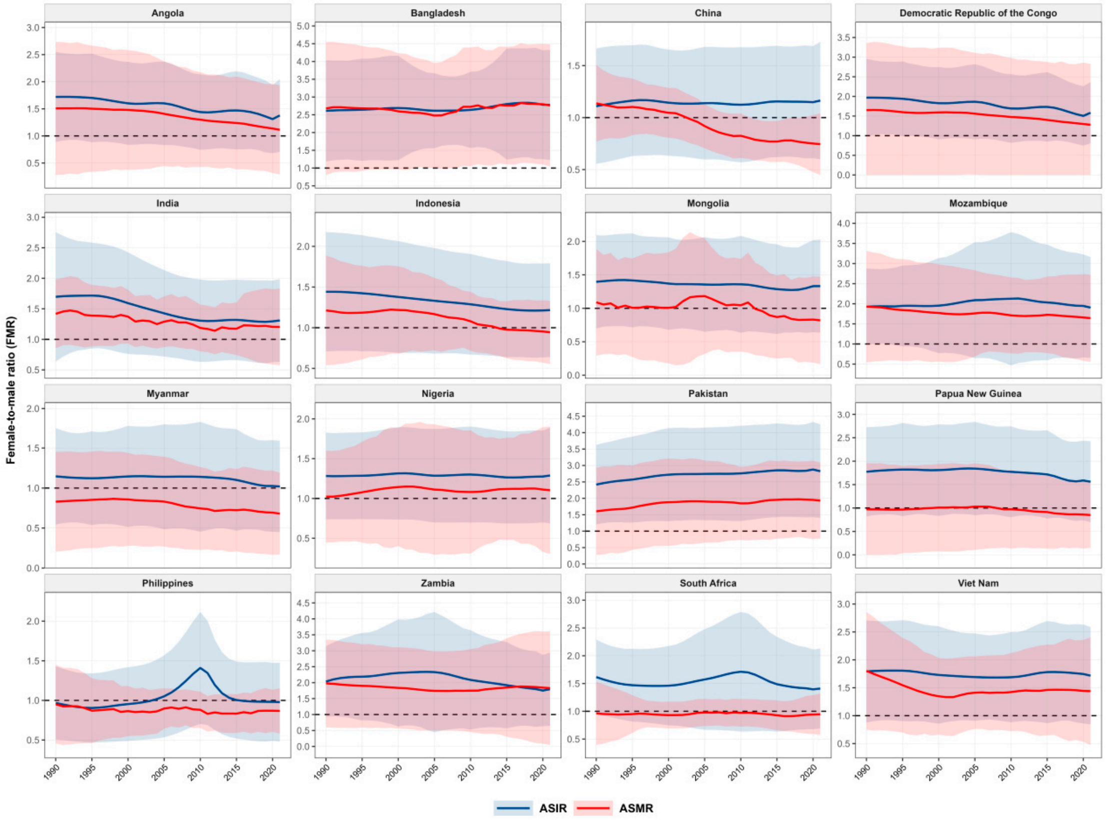

Figure S3. Temporal trends in sex disparities (female-to-male ratio) for pediatric all-form TB across 16 high-burden countries. This figure presents the temporal trends in sex disparities for pediatric all-form TB, quantified using the female-to-male ratio (FMR) of age-standardized rates among HIV-negative children aged 0 – 14 years. Each panel presents data for one of the 16 selected high-burden countries. Within each panel, blue lines represent the FMR of the age-standardized incidence rate (ASIR), and red lines represent the FMR of the age-standardized mortality rate (ASMR). Solid lines indicate annual point estimates, while shaded areas represent approximate 95% uncertainty intervals (UIs) derived via delta-method-based uncertainty propagation. The horizontal dashed line at 1.0 denotes sex parity; an FMR > 1 indicates a higher

burden in females, whereas an FMR < 1 indicates a higher burden in males. Abbreviations: FMR, female-to-male ratio; TB, tuberculosis; ASIR, age-standardized incidence rate; ASMR, age-standardized mortality rate; UI, uncertainty interval.

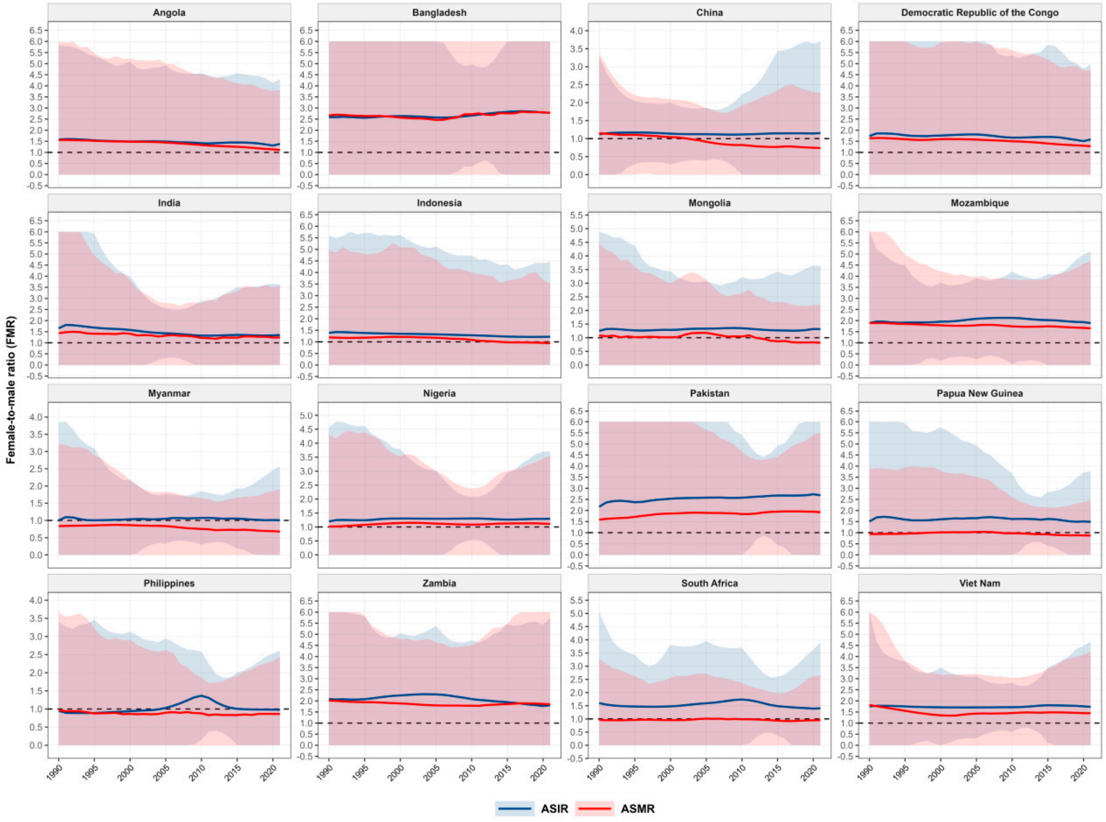

Figure S4. Temporal trends in sex disparities (female-to-male ratio) for pediatric MDR-TB across 16 high-burden countries. This figure presents the temporal trends in sex disparities for pediatric multidrug-resistant tuberculosis (MDR-TB), quantified using the female-to-male ratio (FMR) of age-standardized rates among HIV-negative children aged 0–14 years. Each panel presents data for one of the 16 selected high-burden countries. Within each panel, blue lines represent the FMR of the age-standardized incidence rate (ASIR), and red lines represent the FMR of the age-standardized mortality rate (ASMR). Solid lines indicate annual point estimates, while shaded areas represent approximate 95% uncertainty intervals (UIs) derived via

delta-method-based uncertainty propagation. The horizontal dashed line at 1.0 denotes sex parity; an FMR > 1 indicates a higher burden in females, whereas an FMR < 1 indicates a higher burden in males. Abbreviations: FMR, female-to-male ratio; MDR-TB, multidrug-resistant tuberculosis; ASIR, age-standardized incidence rate; ASMR, age-standardized mortality rate; UI, uncertainty interval.

Supplementary Tables

Table S1: Detailed estimates of pediatric all-form TB and MDR-TB by SDI stratum and GBD region in 2021

Location	All-form TB						MDR-TB					
	ASIR	ASMR	ASDR	Incident cases	Deaths	DALYs	ASIR	ASMR	ASDR	Incident cases	Deaths	DALYs
Global	38.13 (26.69,52.6)	3.68 (2.75,4.67)	346.01 (264.75,436.03)	759300 (596055,949256)	70659 (53652,89521)	6646694 (5198363,8305381)	1.63 (0.94,2.73)	0.31 (0.13,0.61)	27.6 (11.87,54.2)	32515 (20968,51288)	5887 (2467,11332)	530364 (235225,1002206)
High SDI	2.15 (1.43,3.13)	0.01 (0.01,0.02)	2.25 (1.77,2.88)	3725 (2767,4915)	23 (20,27)	3690 (2980,4556)	0.05 (0.03,0.09)	0 (0,0)	0.08 (0.04,0.17)	89 (55,148)	1 (0,3)	136 (65,280)
High-middle SDI	8.31 (5.77,11.79)	0.17 (0.14,0.21)	19.04 (15.68,22.69)	19079 (14353,24843)	368 (311,436)	40826 (34410,47322)	0.69 (0.37,1.21)	0.01 (0.01,0.03)	1.52 (0.83,2.58)	1609 (931,2583)	31 (15,54)	3255 (1773,5437)
Middle SDI	28.68 (19.97,39.45)	1.09 (0.91,1.33)	111.82 (93.9,133.36)	161888 (125273,205207)	5865 (4922,7022)	598722 (516868,694162)	1.15 (0.57,2.03)	0.08 (0.03,0.16)	7.67 (3.24,14.85)	6500 (3464,11030)	437 (177,855)	41055 (17840,77869)
Low-middle SDI	43.3 (29.78,60.14)	3.71 (2.91,4.56)	348.75 (281.7,424.17)	250778 (194694,317548)	20873 (16792,25038)	1957056 (1610770,2327188)	2.11 (1,3.99)	0.36 (0.13,0.74)	32.43 (11.69,65.53)	12217 (6110,22486)	2033 (698,4153)	182112 (65508,367132)
Low SDI	69.74 (49.16,96.19)	9.25 (6.48,12.31)	860.27 (618.55,1123.69)	323363 (255117,404199)	43484 (30513,58126)	4042306 (2925842,5274590)	2.61 (1.47,4.42)	0.72 (0.28,1.52)	64.59 (26.46,135.08)	12087 (7218,18796)	3383 (1340,7079)	303575 (125140,631806)
Andean Latin America	17.41 (11.83,24.68)	0.85 (0.64,1.11)	79.99 (61.47,103.32)	3165 (2396,4159)	152 (122,192)	14305 (11588,17617)	1.18 (0.5,2.52)	0.1 (0.04,0.23)	9.27 (3.3,20.4)	215 (95,430)	18 (6,40)	1659 (596,3561)
Australasia	1.6 (1.04,2.34)	0 (0,0)	1.49 (0.99,2.19)	90 (68,118)	0 (0,0)	81 (56,115)	0.04 (0.02,0.1)	0 (0,0)	0.05 (0.02,0.12)	2 (1,5)	0 (0,0)	3 (1,6)
Caribbean	20.92 (14.45,29.33)	2.47 (1.61,4.14)	218.34 (144.23,360.51)	2402 (1843,3038)	277 (189,452)	24460 (16912,39570)	0.09 (0.02,0.25)	0.02 (0,0.08)	1.76 (0.25,6.94)	10 (3,29)	2 (0,9)	198 (27,768)
Central Asia	14.83 (10.3,20.48	1.21 (0.95,1.	112.27 (89.11,140.	4087 (3216,5226)	343 (275,430)	31855 (25719,39318	3.14 (1.78,5.	0.3 (0.15,0.	27.88 (14.37,44.	866 (544,1388)	86 (44,140)	7909 (4092,12749

Location	All-form TB						MDR-TB					
	ASIR	ASMR	ASDR	Incident cases	Deaths	DALYs	ASIR	ASMR	ASDR	Incident cases	Deaths	DALYs
	)	53)	71)			)	21)	49)	84)			)
Central Europe	3.78 (2.53,5.37)	0.08 (0.06,0.09)	8.36 (6.84,10.14)	678 (515,893)	13 (10,15)	1358 (1144,1582)	0.08 (0.03,0.16)	0 (0,0.01)	0.35 (0.1,0.84)	14 (6,27)	1 (0,1)	57 (17,136)
Central Latin America	4.17 (2.86,5.96)	0.18 (0.14,0.22)	16.93 (13.59,21.01)	2666 (1998,3422)	110 (89,136)	10382 (8433,12586)	0.12 (0.05,0.23)	0.01 (0,0.02)	0.89 (0.3,1.95)	75 (33,149)	6 (2,13)	549 (189,1206)
Central Sub-Saharan Africa	144.27 (102.38,197.51)	14.82 (9.16,23.32)	1399.77 (907.07,2167.87)	85205 (66777,105554)	8850 (5582,13922)	836340 (547576,1278083)	3.24 (0.98,8.38)	0.75 (0.18,2.34)	67.15 (16.7,210.35)	1913 (603,4866)	445 (110,1437)	40123 (10185,128079)
East Asia	10.62 (7.56,14.67)	0.16 (0.13,0.2)	19.56 (15.98,24.11)	27575 (21333,34933)	399 (327,504)	47858 (39637,58118)	0.44 (0.11,1.29)	0.01 (0,0.03)	1.35 (0.32,3.54)	1147 (290,3126)	32 (8,81)	3290 (782,8601)
Eastern Europe	9.31 (5.79,13.82)	0.1 (0.09,0.11)	11.84 (10.2,13.69)	3430 (2405,4841)	32 (29,35)	3716 (3254,4225)	2.66 (1.33,4.66)	0.03 (0.02,0.05)	3.58 (2.03,5.09)	982 (528,1656)	10 (6,15)	1119 (632,1582)
Eastern Sub-Saharan Africa	76.31 (51.57,107.02)	8.84 (6.32,11.94)	835.84 (615.65,1109.52)	136955 (106318,174344)	16043 (11538,21549)	1516737 (1127159,1999744)	2.94 (1.57,5.12)	0.78 (0.27,1.72)	70.84 (25.02,153.51)	5290 (3110,8557)	1422 (489,3123)	128637 (45698,279409)
High-income Asia Pacific	0.84 (0.54,1.25)	0.02 (0.01,0.02)	1.95 (1.63,2.37)	191 (136,263)	4 (3,4)	410 (352,479)	0.01 (0,0.03)	0 (0,0)	0.03 (0.01,0.11)	2 (1,6)	0 (0,0)	7 (2,24)
High-income North America	0.8 (0.52,1.18)	0.01 (0.01,0.01)	1.18 (0.9,1.57)	518 (384,683)	4 (3,4)	729 (571,935)	0.01 (0,0.03)	0 (0,0)	0.02 (0.01,0.07)	8 (3,17)	0 (0,0)	15 (5,40)
North Africa and Middle East	12.01 (8.17,17.08)	0.97 (0.71,1.32)	86.82 (65.14,116.63)	22027 (16895,28087)	1721 (1311,2309)	154498 (119467,204086)	0.74 (0.31,1.79)	0.14 (0.03,0.43)	12.26 (2.8,37.46)	681 (307,1564)	124 (27,377)	10904 (2494,32841)

Location	All-form TB						MDR-TB					
	ASIR	ASMR	ASDR	Incident cases	Deaths	DALYs	ASIR	ASMR	ASDR	Incident cases	Deaths	DALYs
Oceania	43.53 (28.9,60.65)	5.45 (3.15,8.17)	483.34 (283.85,719.62)	2201 (1650,2852)	289 (174,422)	25755 (15869,37301)	1.51 (0.42,3.64)	0.37 (0.08,1.03)	32.72 (7.03,89.67)	77 (22,173)	20 (4,55)	1747 (369,4832)
South Asia	38.01 (25.88,53.12)	3.25 (2.59,3.99)	304.6 (247.03,372.85)	194113 (147300,254329)	15635 (12731,18528)	1461670 (1217873,1722256)	5.19 (1.76,11.63)	0.81 (0.24,1.83)	73.28 (22.36,163.52)	13226 (4816,28148)	1959 (589,4327)	175553 (56903,379967)
Southeast Asia	56.09 (39.1,77.54)	2.65 (2.12,3.24)	259.88 (212.18,313.94)	96962 (75163,122721)	4420 (3634,5312)	433107 (362333,510531)	1.11 (0.48,2.24)	0.09 (0.03,0.21)	8.69 (3.16,18.6)	1922 (862,3791)	157 (53,348)	14483 (5310,31187)
Southern Latin America	4.99 (3.22,7.19)	0.1 (0.09,0.12)	10.76 (8.97,12.9)	740 (544,998)	14 (13,16)	1474 (1267,1704)	0.06 (0.01,0.2)	0 (0,0.01)	0.24 (0.05,0.76)	9 (2,31)	0 (0,1)	32 (7,103)
Southern Sub-Saharan Africa	164.93 (108.82,230.78)	11.11 (8.7,14.18)	1080.92 (869.36,1341.47)	39541 (29922,50817)	2601 (2064,3248)	252989 (208357,307226)	6.32 (2.66,14.67)	0.96 (0.33,2.23)	87.63 (30.89,199.57)	1513 (688,3274)	225 (77,520)	20484 (7307,46220)
Tropical Latin America	5.98 (3.94,8.62)	0.22 (0.17,0.27)	22.13 (17.5,27.58)	3018 (2230,3913)	108 (85,134)	10972 (8980,13333)	0.19 (0.04,0.59)	0.01 (0,0.04)	1.23 (0.25,3.48)	98 (21,296)	6 (1,18)	610 (126,1702)
Western Europe	2.51 (1.58,3.8)	0.01 (0,0.01)	1.33 (0.95,1.86)	1741 (1248,2417)	3 (3,4)	857 (631,1146)	0.05 (0.03,0.1)	0 (0,0)	0.04 (0.02,0.07)	37 (22,60)	0 (0,0)	25 (13,44)
Western Sub-Saharan Africa	60.29 (42.87,81.92)	8.71 (5.63,13.32)	805.46 (536.76,1208.95)	131997 (105932,163521)	19641 (12785,30127)	1817142 (1209776,2716545)	2.02 (0.85,4.5)	0.61 (0.18,1.65)	54.46 (16.33,146.1)	4428 (1986,9775)	1373 (407,3701)	122961 (37060,327219)

Note: Data are presented as estimates with 95% uncertainty intervals (UIs) in parentheses. The categories of all-form TB include both drug-susceptible and drug-resistant forms of tuberculosis. Abbreviations: TB, tuberculosis; MDR-TB, multidrug-resistant tuberculosis; ASIR, age-standardized incidence rate (per 100,000 population); ASMR, age-standardized mortality rate (per 100,000 population); ASDR, age-standardized DALY rate (per 100,000 population); DALYs, disability-adjusted life years; SDI, socio-demographic index.

Table S2: Detailed estimates of pediatric all-form TB and MDR-TB for 204 countries and territories in 2021

Country and territory	All-form TB						MDR-TB					
	ASIR	ASMR	ASDR	Incident cases	Deaths	DALYs	ASIR	ASMR	ASDR	Incident cases	Deaths	DALYs
Afghanistan	53.36 (35.04,76.14)	8.01 (5.49,11.74)	702.93 (486.99,1021.87)	7489 (5725,9694)	1203 (869,1716)	105917 (76955,150464)	2.2983 (0.301,8.4882)	0.649 (0.0809,2.3079)	56.5094 (7.0694,200.5949)	326 (45,1124)	97 (12,353)	8517 (1068,30738)
Albania	3.46 (2.3,4.99)	0.13 (0.09,0.19)	12.07 (8.72,17.39)	15 (11,20)	1 (0,1)	50 (37,70)	0.024 (0.0037,0.0795)	0.0017 (0.0002,0.0068)	0.1594 (0.0205,0.6101)	0 (0,0)	0 (0,0)	1 (0,3)
Algeria	15.96 (10.28,23.13)	0.45 (0.31,0.68)	40.46 (28.28,60.64)	2107 (1556,2856)	60 (44,86)	5411 (4034,7522)	0.2928 (0.0337,1.104)	0.0187 (0.0017,0.0736)	1.6412 (0.152,6.4265)	39 (5,144)	3 (0,10)	219 (21,861)
American Samoa	3.49 (2.29,5.13)	0.12 (0.09,0.17)	12.18 (8.74,16.69)	0 (0,1)	0 (0,0)	2 (1,2)	0.0098 (0.001,0.0398)	0.0008 (0.0001,0.0031)	0.0677 (0.0061,0.2751)	0 (0,0)	0 (0,0)	0 (0,0)
Andorra	1.96 (1.26,2.91)	0 (0,0)	0.21 (0.14,0.29)	0 (0,0)	0 (0,0)	0 (0,0)	0.0043 (0.0005,0.016)	0 (0,0)	0.0007 (0,0.0026)	0 (0,0)	0 (0,0)	0 (0,0)
Angola	119.89 (84.29,165.17)	12.82 (7.84,18.25)	1212.47 (781.35,1697.39)	18541 (14594,23164)	2034 (1280,2860)	192553 (128077,262082)	3.0191 (0.4298,9.2634)	0.7225 (0.0897,2.4602)	65.1783 (8.2277,221.7673)	467 (68,1414)	115 (14,397)	10353 (1305,35490)
Antigua and Barbuda	7.58 (5.01,11.05)	0.02 (0.02,0.02)	5.23 (3.13,8.41)	1 (1,2)	0 (0,0)	1 (1,1)	0.05 (0.0064,0.2102)	0.0003 (0,0.001)	0.0419 (0.0052,0.1695)	0 (0,0)	0 (0,0)	0 (0,0)
Argentina	5.72 (3.69,8.32)	0.11 (0.09,0.13)	11.12 (8.99,13.7)	598 (437,815)	10 (9,12)	1073 (906,1258)	0.0776 (0.0106,0.2774)	0.0027 (0.0003,0.0101)	0.2541 (0.032,0.96)	8 (1,30)	0 (0,1)	25 (3,92)
Armenia	4.76 (3.17,6.88)	0.15 (0.12,0.18)	15.82 (12.36,20.04)	28 (21,37)	1 (1,1)	87 (70,106)	0.7156 (0.1586,1.8002)	0.0319 (0.0073,0.0708)	3.2391 (0.7399,7.153)	4 (1,10)	0 (0,0)	18 (4,39)
Australia	1.33 (0.88,1.94)	0 (0,0.01)	1.25 (0.86,1.76)	62 (47,79)	0 (0,0)	56 (39,77)	0.0426 (0.0126,0.1062)	0.0002(0,0.0007)	0.0517 (0.0157,0.1262)	2 (1,5)	0 (0,0)	2 (1,6)
Austria	2.33	0 (0,0)	1.33	30 (22,41)	0 (0,0)	17 (12,24)	0.0689	0.0002	0.0488	1 (0,2)	0 (0,0)	1 (0,1)

Country and territory	All-form TB						MDR-TB					
	ASIR	ASMR	ASDR	Incident cases	Deaths	DALYs	ASIR	ASMR	ASDR	Incident cases	Deaths	DALYs
	(1.48,3.55)		(0.89,1.95)				(0.0194,0.1674)	(0,0.0006)	(0.0138,0.1133)			
Azerbaijan	13.11 (8.83,18.36)	1.22 (0.83,1.75)	113.35 (79.22,159.59)	310 (234,401)	26 (19,36)	2435 (1812,3315)	2.8474 (0.8241,6.4503)	0.3128 (0.0963,0.647)	28.5509 (8.9381,58.7048)	67 (21,143)	7 (2,14)	613 (196,1235)
Bahamas	4.98 (3.29,7.21)	0.08 (0.06,0.11)	9.24 (6.82,12.25)	4 (3,6)	0 (0,0)	7 (5,8)	0.0683 (0.0086,0.2574)	0.0023 (0.0002,0.0091)	0.2323 (0.0256,0.8595)	0 (0,0)	0 (0,0)	0 (0,1)
Bahrain	5 (3.28,7.28)	0.07 (0.05,0.1)	7.89 (5.96,10.77)	15 (11,20)	0 (0,0)	22 (18,29)	0.1334 (0.0197,0.5254)	0.004 (0.0005,0.0157)	0.3927 (0.0535,1.4878)	0 (0,1)	0 (0,0)	1 (0,4)
Bangladesh	40.93 (27.16,58.4)	2.44 (1.63,3.4)	229.03 (158.4,312.4)	19098 (14211,25977)	1095 (821,1403)	102421 (78637,128670)	1.5376 (0.3972,4.1701)	0.1997 (0.0434,0.5281)	17.7108 (4.0099,46.4734)	718 (193,1914)	90 (21,231)	7915 (1896,20125)
Barbados	2.4 (1.54,3.55)	0.02 (0.01,0.03)	2.87 (1.95,4.11)	1 (1,2)	0 (0,0)	1 (1,2)	0.0078 (0.0006,0.036)	0.0001 (0,0.0006)	0.0148 (0.0012,0.0681)	0 (0,0)	0 (0,0)	0 (0,0)
Belarus	2.89 (1.89,4.24)	0.06 (0.04,0.08)	6.95 (5.18,9.23)	45 (33,60)	1 (1,1)	99 (78,124)	1.0938 (0.5603,1.8702)	0.0257 (0.0136,0.04)	2.8523 (1.6065,4.3621)	17 (10,27)	0 (0,1)	40 (24,60)
Belgium	2.77 (1.76,4.15)	0 (0,0)	1.36 (0.89,2.01)	53 (39,73)	0 (0,0)	25 (17,35)	0.0527 (0.0123,0.1474)	0.0001 (0,0.0004)	0.0299 (0.0071,0.0807)	1 (0,3)	0 (0,0)	1 (0,1)
Belize	8.3 (5.33,12.02)	0.25 (0.2,0.32)	24.99 (19.93,30.51)	10 (8,14)	0 (0,0)	29 (24,34)	0.0403 (0.0045,0.1641)	0.0025 (0.0002,0.0105)	0.2313 (0.0217,0.9767)	0 (0,0)	0 (0,0)	0 (0,1)
Benin	41.84 (29.18,58.25)	4.38 (2.26,10.05)	418.37 (231.77,911.28)	2625 (2097,3291)	287 (151,651)	27444 (15587,59574)	0.8434 (0.1491,2.5593)	0.2107 (0.0253,0.7397)	19.1135 (2.4408,65.9447)	53 (10,154)	14 (2,48)	1256 (162,4247)
Bermuda	6.1 (4,8.98)	0 (0,0)	3.02 (1.5,5.26)	1 (0,1)	0 (0,0)	0 (0,0)	0.0315 (0.0022,0.1581)	0 (0,0.0001)	0.0162 (0.0012,0.0832)	0 (0,0)	0 (0,0)	0 (0,0)

Country and territory	All-form TB						MDR-TB					
	ASIR	ASMR	ASDR	Incident cases	Deaths	DALYs	ASIR	ASMR	ASDR	Incident cases	Deaths	DALYs
Bhutan	20.25 (13.33,29.42)	1.33 (0.68,2.43)	127.52 (68.12,222.58)	38 (28,52)	2 (1,4)	229 (134,393)	0.8089 (0.1295,2.5995)	0.1139 (0.014,0.3791)	10.3278 (1.3236,33.9741)	2 (0,5)	0 (0,1)	19 (2,60)
Bolivia (Plurinational State of)	20.8 (14.36,29.15)	1.95 (1.34,2.73)	178.23 (124,246.58)	726 (558,930)	67 (48,90)	6146 (4476,8100)	0.9921 (0.1625,3.3059)	0.1843 (0.0272,0.6029)	16.3433 (2.4384,53.5622)	35 (6,113)	6 (1,21)	564 (85,1854)
Bosnia and Herzegovina	4.02 (2.64,5.89)	0.08 (0.05,0.1)	8.44 (6.12,11.11)	20 (15,27)	0 (0,0)	39 (30,49)	0.0172 (0.0036,0.0543)	0.0006 (0.0001,0.0021)	0.0632 (0.0126,0.2033)	0 (0,0)	0 (0,0)	0 (0,1)
Botswana	129.74 (89.3,180.12)	9.25 (5.57,14.18)	896.37 (575.62,1323.71)	902 (710,1138)	63 (41,94)	6107 (4130,8701)	6.352 (1.249,19.3206)	0.9781 (0.1625,3.0398)	89.235 (15.2203,272.1403)	44 (9,132)	7 (1,21)	608 (103,1845)
Brazil	5.97 (3.91,8.6)	0.21 (0.16,0.26)	21.11 (16.78,26.34)	2888 (2126,3750)	98 (78,121)	10064 (8246,12172)	0.1931 (0.0367,0.6008)	0.0123 (0.0021,0.038)	1.1656 (0.205,3.5542)	93 (17,290)	6 (1,18)	555 (100,1682)
Brunei Darussalam	4.15 (2.63,6.23)	0.19 (0.14,0.25)	17.85 (13.35,23.44)	4 (3,5)	0 (0,0)	16 (13,20)	0.0114 (0.0013,0.0439)	0.0011 (0.0001,0.004)	0.0979 (0.0096,0.3568)	0 (0,0)	0 (0,0)	0 (0,0)
Bulgaria	5.98 (3.91,8.61)	0.03 (0.02,0.04)	4.7 (3.41,6.59)	60 (43,78)	0 (0,0)	43 (33,56)	0.123 (0.0264,0.3655)	0.0011 (0.0002,0.0037)	0.1419 (0.0288,0.427)	1 (0,4)	0 (0,0)	1 (0,4)
Burkina Faso	58.74 (41.79,80.06)	8.93 (5.39,15.01)	836.06 (530.87,1362.13)	6445 (5086,8020)	1025 (636,1675)	95903 (62075,153204)	1.6622 (0.2767,4.8584)	0.5569 (0.0741,1.8137)	50.2541 (6.8909,163.6297)	183 (31,534)	64 (9,204)	5774 (798,18353)
Burundi	90.75 (62.63,127.03)	9.96 (5.45,15.61)	950.43 (557.36,1442.96)	5374 (4180,6899)	605 (341,926)	57759 (34872,86306)	2.9788 (0.4643,9.7602)	0.7456 (0.0928,2.4923)	67.5911 (8.584,227.4558)	176 (29,555)	45 (5,151)	4111 (522,13785)
Cabo Verde	25.59 (17.99,35.41)	0.45 (0.25,1.06)	60.89 (40.13,115.79)	36 (28,45)	1 (0,1)	82 (57,144)	0.8096 (0.125,2.3537)	0.0321 (0.004,0.1221)	3.4468 (0.4596,11.5377)	1 (0,3)	0 (0,0)	5 (1,16)
Cambodia	66.27	4.05	381.53	3395	205	19293	0.4567	0.0615	5.5109	23 (4,83)	3	279

Country and territory	All-form TB						MDR-TB					
	ASIR	ASMR	ASDR	Incident cases	Deaths	DALYs	ASIR	ASMR	ASDR	Incident cases	Deaths	DALYs
	(45.44,94.67)	(2.37,6.55)	(236.57,595.02)	(2575,4349)	(121,324)	(12027,29234)	(0.0704,1.6064)	(0.007,0.2385)	(0.641,21.0558)		(0,12)	(34,1072)
Cameroon	58.21 (39.9,81.24)	4.61 (2.61,8.89)	445.09 (271.08,816.48)	7901 (6081,9962)	635 (366,1217)	61385 (38057,112313)	1.3691 (0.2108,4.2485)	0.2461 (0.0317,0.8085)	22.467 (2.9901,72.6574)	186 (30,563)	34 (4,109)	3100 (416,9685)
Canada	1.4 (0.92,2.04)	0 (0,0.01)	1.11 (0.79,1.55)	86 (63,112)	0 (0,0)	64 (47,88)	0.0183 (0.005,0.049)	0.0001(0,0.0004)	0.0199 (0.0052,0.052)	1 (0,3)	0 (0,0)	1 (0,3)
Central African Republic	260.5 (177.7,364.4)	56.21 (36.58,81.05)	5085 (3373.35,7229.22)	6040 (4688,7512)	1332 (897,1888)	120570 (81708,168199)	4.0385 (0.6356,13.9011)	1.8802 (0.2127,6.7387)	166.4314 (19.5786,593.1391)	94 (16,306)	45 (5,157)	3949 (463,13867)
Chad	96.17 (67.6,132.4)	15.74 (10.53,22.79)	1439.96 (972.96,2056.12)	9021 (7197,11323)	1579 (1069,2252)	144702 (99458,203451)	2.4985 (0.4041,7.4539)	0.9008 (0.1365,2.8246)	80.1787 (12.3649,249.3032)	235 (38,648)	90 (14,290)	8069 (1228,25536)
Chile	2.68 (1.71,3.93)	0.11 (0.09,0.12)	10.45 (8.83,12.22)	99 (72,135)	4 (3,4)	360 (314,407)	0.0294 (0.0077,0.0785)	0.0024 (0.0005,0.0071)	0.2229 (0.0478,0.6348)	1 (0,3)	0 (0,0)	8 (2,21)
China	9.47 (6.67,13.15)	0.13 (0.1,0.17)	16.69 (13.46,20.78)	23906 (18490,30217)	311 (252,387)	39682 (32374,48484)	0.3903 (0.0608,1.2739)	0.01 (0.0014,0.0301)	1.0904 (0.1712,3.2758)	984 (160,3021)	24 (3,70)	2588 (405,7710)
Colombia	3.33 (2.31,4.68)	0.13 (0.1,0.19)	13.16 (9.64,17.63)	351 (275,443)	14 (10,19)	1352 (1034,1769)	0.1455 (0.0251,0.4434)	0.0114 (0.0017,0.0332)	1.0407 (0.1682,2.9752)	15 (3,47)	1 (0,3)	107 (17,304)
Comoros	45.43 (31.89,61.82)	5.83 (3.57,8.68)	552.43 (352.08,805.51)	108 (84,136)	14 (9,20)	1294 (842,1832)	4.245 (0.5721,13.8842)	0.8317 (0.0906,2.6004)	75.5277 (8.2294,237.8562)	10 (1,33)	2 (0,6)	177 (19,551)
Congo	91.49 (60.42,130.26)	5.33 (3.01,8.06)	532.15 (326.61,772.92)	1736 (1279,2251)	99 (57,146)	9866 (6127,13999)	1.7512 (0.2499,6.0953)	0.2374 (0.0264,0.889)	21.8806 (2.5131,81.2313)	33 (5,115)	4 (0,17)	405 (48,1490)
Cook Islands	3.19 (2.12,4.74)	0.02 (0.02,0.03)	3.39 (2.38,4.87)	0 (0,0)	0 (0,0)	0 (0,0)	0.0084 (0.0009,0.0324)	0.0001(0,0.0005)	0.0147 (0.0016,0.0588)	0 (0,0)	0 (0,0)	0 (0,0)

Country and territory	All-form TB						MDR-TB					
	ASIR	ASMR	ASDR	Incident cases	Deaths	DALYs	ASIR	ASMR	ASDR	Incident cases	Deaths	DALYs
Costa Rica	1.57 (1.03,2.31)	0.04 (0.03,0.04)	3.99 (3.29,4.82)	16 (12,21)	0 (0,0)	39 (34,45)	0.0304 (0.0043,0.1107)	0.0015 (0.0002,0.0055)	0.1462 (0.0185,0.5191)	0 (0,1)	0 (0,0)	1 (0,5)
Côte d'Ivoire	2.04 (1.34,3.01)	0.03 (0.02,0.03)	3.34 (2.59,4.21)	12 (9,17)	0 (0,0)	18 (15,22)	0.0063 (0.0014,0.0192)	0.0001 (0,0.0005)	0.0175 (0.0034,0.0538)	0 (0,0)	0 (0,0)	0 (0,0)
Croatia	1.42 (0.97,2.05)	0.01 (0.01,0.01)	1.82 (1.35,2.45)	24 (19,31)	0 (0,0)	30 (23,39)	0.0329 (0.0057,0.1042)	0.0004 (0.0001,0.0014)	0.0596 (0.0106,0.1955)	1 (0,2)	0 (0,0)	1 (0,3)
Cuba	1.35 (0.86,2)	0 (0,0.01)	0.81 (0.57,1.13)	3 (2,4)	0 (0,0)	2 (1,2)	0.0201 (0.0035,0.0619)	0.0001(0,0.0004)	0.017 (0.0029,0.0544)	0 (0,0)	0 (0,0)	0 (0,0)
Cyprus	1.21 (0.77,1.82)	0 (0,0)	0.98 (0.69,1.37)	21 (15,28)	0 (0,0)	16 (12,22)	0.0211 (0.0056,0.055)	0.0001(0,0.0003)	0.022 (0.0057,0.057)	0 (0,1)	0 (0,0)	0 (0,1)
Czechia	63.3 (44.28,87.42)	4.69 (2.73,8.59)	455.96 (285.23,795.99)	7444 (5781,9322)	572 (344,1040)	55641 (35686,95817)	2.0234 (0.3558,5.964)	0.3464 (0.0487,1.1408)	31.6925 (4.4624,104.8215)	238 (42,703)	42 (6,138)	3871 (556,12852)
Democratic People's Republic of Korea	70.41 (49.54,99.02)	1.9 (1.17,2.96)	173.01 (110.26,264.43)	3336 (2540,4186)	86 (56,128)	7830 (5193,11427)	3.2948 (0.9119,8.2182)	0.1726 (0.0357,0.4949)	15.2847 (3.2534,43.634)	156 (45,381)	8 (2,22)	692 (147,1968)
Democratic Republic of the Congo	151.82 (107.23,208.04)	13.9 (7.91,25.5)	1323.11 (799.36,2350.54)	57984 (45483,72972)	5347 (3201,9907)	509372 (319729,897620)	3.3873 (0.5433,10.7928)	0.7266 (0.0947,2.8061)	65.4491 (8.7123,251.3647)	1294 (216,4026)	280 (36,1071)	25200 (3379,95857)
Denmark	2 (1.25,3)	0 (0,0)	1.05 (0.66,1.59)	19 (14,26)	0 (0,0)	9 (6,14)	0.0328 (0.0073,0.0958)	0 (0,0.0002)	0.0179 (0.0039,0.0488)	0 (0,1)	0 (0,0)	0 (0,0)
Djibouti	48.98 (32.92,69.39)	4.97 (2.79,7.71)	477.45 (283.65,711.85)	203 (155,261)	21 (12,32)	1984 (1202,2931)	3.377 (1.1321,8.4876)	0.7307 (0.189,1.8607)	66.8336 (17.7507,167.3413)	14 (5,34)	3 (1,8)	278 (75,689)

Country and territory	All-form TB						MDR-TB					
	ASIR	ASMR	ASDR	Incident cases	Deaths	DALYs	ASIR	ASMR	ASDR	Incident cases	Deaths	DALYs
Dominica	6.26 (4.26,9)	0.34 (0.23,0.5)	32.66 (22.29,45.79)	1 (1,1)	0 (0,0)	4 (3,5)	0.0379 (0.0047,0.133)	0.0041 (0.0004,0.0157)	0.3627 (0.0409,1.4003)	0 (0,0)	0 (0,0)	0 (0,0)
Dominican Republic	15.55 (10.65,22.22)	0.64 (0.42,0.97)	59.25 (39.93,87.73)	458 (347,592)	19 (13,27)	1743 (1280,2404)	0.0748 (0.0094,0.2947)	0.0066 (0.0007,0.0284)	0.5785 (0.0633,2.4418)	2 (0,9)	0 (0,1)	17 (2,67)
Ecuador	10.18 (6.78,14.55)	0.29 (0.22,0.39)	29.38 (21.92,39)	520 (392,673)	15 (11,19)	1455 (1144,1835)	0.5004 (0.0759,1.7445)	0.0283 (0.0042,0.0884)	2.6203 (0.3905,8.1368)	26 (4,88)	1 (0,4)	130 (20,409)
Egypt	3.48 (2.37,4.94)	0.11 (0.08,0.14)	11.23 (8.36,14.82)	1276 (980,1611)	40 (31,51)	4160 (3235,5216)	0.1536 (0.0332,0.4842)	0.01 (0.0017,0.0318)	0.9418 (0.1676,2.9638)	56 (12,168)	4 (1,12)	349 (61,1094)
El Salvador	7.92 (5.24,11.56)	0.2 (0.13,0.28)	18.1 (12.65,24.95)	144 (108,185)	4 (3,5)	323 (239,420)	0.1445 (0.0203,0.4992)	0.0077 (0.0008,0.0265)	0.6778 (0.078,2.3235)	3 (0,9)	0 (0,0)	12 (1,41)
Equatorial Guinea	74.44 (50.4,104.21)	3.61 (1.49,7.45)	375.52 (191.34,707.04)	427 (324,552)	20 (8,39)	2076 (1111,3741)	1.9051 (0.3058,6.3133)	0.2075 (0.0226,0.7877)	19.5356 (2.2413,71.3217)	11 (2,36)	1 (0,4)	108 (13,400)
Eritrea	123.7 (87.71,167.55)	13.5 (7.69,23.77)	1310.4 (797.06,2210.93)	3169 (2485,3920)	351 (209,613)	34084 (21317,57585)	4.7334 (0.83,14.4441)	1.1356 (0.1542,3.9156)	103.9385 (14.4817,353.8929)	121 (21,356)	30 (4,102)	2705 (390,9176)
Estonia	3.58 (2.28,5.38)	0.03 (0.02,0.04)	4.14 (3.26,5.31)	8 (6,11)	0 (0,0)	8 (7,10)	0.6794 (0.2643,1.4057)	0.0078 (0.0032,0.0135)	0.9704 (0.4101,1.7308)	1 (1,3)	0 (0,0)	2 (1,3)
Eswatini	188.68 (112.49,283.6)	12.09 (7.92,18.02)	1169.46 (797.08,1679.51)	778 (545,1090)	49 (34,70)	4745 (3349,6583)	20.036 (4.1563,55.0725)	2.4742 (0.5426,6.2068)	226.798 (50.5594,563.9339)	83 (20,233)	10 (2,25)	920 (206,2269)
Ethiopia	62.79 (43.63,86.89)	5.7 (4.1,7.57)	549.67 (404.34,717.69)	28129 (21785,35155)	2583 (1863,3388)	248834 (185632,318140)	2.0324 (0.2624,6.9511)	0.3989 (0.0561,1.2977)	36.4847 (5.3688,118.3348)	911 (121,3105)	181 (25,599)	16529 (2450,54886)
Fiji	15.79 (10.61,22.	0.67 (0.46,0.9	61.46 (42.88,84.0	43 (33,56)	2 (1,2)	166 (125,213)	0.0267 (0.0025,0.125)	0.0025 (0.0002,0.0119)	0.2186 (0.0183,1.04	0 (0,0)	0 (0,0)	1 (0,3)

Country and territory	All-form TB						MDR-TB					
	ASIR	ASMR	ASDR	Incident cases	Deaths	DALYs	ASIR	ASMR	ASDR	Incident cases	Deaths	DALYs
	77)	4)	7)					)	64)			
Finland	1.82 (1.13,2.78)	0 (0,0.01)	1.17 (0.82,1.67)	15 (11,22)	0 (0,0)	9 (7,12)	0.0549 (0.0127,0.1538)	0.0003 (0.0001,0.0008)	0.0464 (0.0108,0.1231)	0 (0,1)	0 (0,0)	0 (0,1)
France	2.11 (1.34,3.16)	0.01 (0.01,0.01)	1.61 (1.23,2.1)	248 (181,340)	1 (1,1)	174 (138,221)	0.0394 (0.0096,0.108)	0.0004 (0.0001,0.001)	0.0443 (0.0103,0.1174)	5 (1,13)	0 (0,0)	5 (1,13)
Gabon	75.32 (50.69,106.14)	2.85 (1.3,4.88)	306.1 (171.34,485.69)	477 (368,610)	18 (8,29)	1903 (1081,2970)	2.253 (0.3664,7.2939)	0.1832 (0.023,0.6859)	17.4407 (2.4218,63.4921)	14 (2,46)	1 (0,4)	108 (16,392)
Gambia	82.17 (57.8,112.89)	4.02 (2.51,7.15)	422.5 (283.33,697.97)	826 (651,1066)	41 (26,67)	4267 (2968,6688)	1.7429 (0.2781,5.848)	0.195 (0.0247,0.7023)	18.3678 (2.4317,64.7019)	18 (3,56)	2 (0,7)	186 (26,673)
Georgia	11.22 (7.45,15.97)	0.18 (0.14,0.23)	20.61 (15.67,26.84)	83 (61,109)	1 (1,2)	147 (117,180)	2.1776 (0.9427,4.0889)	0.0462 (0.0182,0.084)	4.9263 (2.0664,8.8154)	16 (7,29)	0 (0,1)	35 (15,60)
Germany	1.96 (1.28,2.89)	0 (0,0)	1.2 (0.79,1.75)	234 (176,310)	0 (0,0)	141 (98,199)	0.0728 (0.0224,0.177)	0.0002 (0,0.0006)	0.0539 (0.0162,0.1286)	9 (3,20)	0 (0,0)	6 (2,15)
Ghana	56.39 (37.89,78.95)	2.83 (1.6,6.77)	284.08 (173.72,630.89)	7292 (5681,9233)	371 (223,861)	37283 (24382,80480)	1.4439 (0.2106,4.9237)	0.169 (0.0175,0.762)	15.6047 (1.7072,69.1753)	187 (28,645)	22 (2,100)	2049 (218,9259)
Greece	1.41 (0.87,2.16)	0.01 (0,0.01)	1.14 (0.84,1.55)	20 (14,27)	0 (0,0)	15 (11,19)	0.0386 (0.0066,0.1203)	0.0003 (0,0.001)	0.0424 (0.0073,0.1404)	1 (0,2)	0 (0,0)	1 (0,2)
Greenland	14.16 (8.85,21.24)	0.11 (0.07,0.16)	12.15 (7.98,16.99)	2 (1,2)	0 (0,0)	1 (1,2)	0.272 (0.0346,1.0365)	0.0041 (0.0005,0.0145)	0.4007 (0.0483,1.4122)	0 (0,0)	0 (0,0)	0 (0,0)
Grenada	4.77 (3.12,6.98)	0.02 (0.02,0.03)	4.52 (3.03,6.36)	1 (1,1)	0 (0,0)	1 (1,1)	0.0337 (0.0039,0.1444)	0.0004 (0,0.0016)	0.0464 (0.0058,0.214)	0 (0,0)	0 (0,0)	0 (0,0)
Guam	13.18	0.33	30.45	5 (4,7)	0 (0,0)	11 (9,14)	0.0471	0.0025	0.2244	0 (0,0)	0 (0,0)	0 (0,0)

Country and territory	All-form TB						MDR-TB					
	ASIR	ASMR	ASDR	Incident cases	Deaths	DALYs	ASIR	ASMR	ASDR	Incident cases	Deaths	DALYs
	(8.73,19.07)	(0.23,0.45)	(22.21,41.01)				(0.0052,0.1848)	(0.0002,0.0101)	(0.0198,0.8792)			
Guatemala	4.91 (3.38,6.84)	0.24 (0.19,0.3)	22.32 (17.43,28.18)	241 (186,306)	11 (9,14)	1072 (883,1317)	0.1321 (0.0211,0.3979)	0.0135 (0.002,0.0412)	1.1974 (0.182,3.6773)	6 (1,19)	1 (0,2)	58 (9,178)
Guinea	56.03 (38.8,77.29)	6.4 (3.7,11.66)	605.16 (370.35,1060.83)	3456 (2697,4429)	405 (238,741)	38307 (24032,67226)	1.1711 (0.1949,3.723)	0.3072 (0.0408,1.0676)	27.7355 (3.8453,95.7634)	72 (11,226)	19 (3,68)	1756 (245,6151)
Guinea-Bissau	77.82 (49.95,115.04)	7.34 (4.37,14.91)	692.56 (434.57,1342.13)	707 (526,940)	68 (42,131)	6447 (4171,12003)	1.4136 (0.2043,4.8653)	0.3081 (0.0339,1.2288)	27.6249 (3.1786,108.9559)	13 (2,44)	3 (0,11)	257 (30,976)
Guyana	12.08 (8.23,17)	0.43 (0.31,0.56)	41.02 (30.55,53.68)	26 (19,34)	1 (1,1)	87 (67,111)	0.077 (0.0084,0.348)	0.0052 (0.0005,0.0219)	0.4728 (0.0478,1.976)	0 (0,1)	0 (0,0)	1 (0,4)
Haiti	40.2 (27.08,56.85)	5.55 (3.51,9.6)	487.81 (312.3,834.06)	1757 (1343,2240)	246 (165,413)	21657 (14585,36015)	0.1441 (0.0199,0.524)	0.0441 (0.0046,0.1874)	3.8405 (0.4032,16.3552)	6 (1,23)	2 (0,8)	171 (18,710)
Honduras	7.61 (5.08,10.84)	0.29 (0.16,0.47)	27.57 (15.7,42.92)	251 (191,327)	9 (6,14)	893 (553,1317)	0.1866 (0.0322,0.5903)	0.0147 (0.0018,0.0486)	1.3247 (0.1714,4.3213)	6 (1,19)	0 (0,2)	43 (6,136)
Hungary	1.38 (0.9,2.02)	0.02 (0.01,0.02)	2.27 (1.77,2.84)	19 (14,26)	0 (0,0)	30 (24,36)	0.0158 (0.0029,0.052)	0.0004 (0,0.0012)	0.0401 (0.0069,0.137)	0 (0,1)	0 (0,0)	1 (0,2)
Iceland	1.99 (1.21,3.15)	0 (0,0.01)	1.08 (0.77,1.5)	1 (1,2)	0 (0,0)	1 (1,1)	0.0146 (0.0021,0.0542)	0 (0,0.0003)	0.0104 (0.0015,0.0355)	0 (0,0)	0 (0,0)	0 (0,0)
India	32.53 (21.84,45.53)	2.26 (1.7,3.04)	215.81 (166.35,284.41)	120670 (90424,157618)	7793 (6098,10088)	740996 (589540,944517)	2.486 (0.5045,6.465)	0.3133 (0.0567,0.8547)	28.4365 (5.2186,76.8791)	9210 (2038,23978)	1079 (204,2831)	97461 (18844,253494)
Indonesia	48.86 (34.25,67.03)	3.39 (2.58,4.35)	321.1 (249.61,405.06)	32830 (25546,41294)	2199 (1733,2766)	207828 (167347,253433)	0.1801 (0.0246,0.7015)	0.0277 (0.0028,0.1085)	2.4885 (0.2666,9.641)	121 (16,458)	18 (2,70)	1610 (172,6264)

Country and territory	All-form TB						MDR-TB					
	ASIR	ASMR	ASDR	Incident cases	Deaths	DALYs	ASIR	ASMR	ASDR	Incident cases	Deaths	DALYs
Iran (Islamic Republic of)	5.51 (3.7,7.88)	0.08 (0.07,0.11)	8.91 (7.25,10.85)	1126 (852,1455)	16 (14,20)	1730 (1467,2036)	0.1032 (0.0278,0.2738)	0.0033 (0.0007,0.0091)	0.3069 (0.0701,0.8248)	21 (6,57)	1 (0,2)	60 (14,158)
Iraq	8.09 (5.36,11.43)	0.2 (0.14,0.28)	20.4 (14.69,27.71)	1096 (814,1426)	26 (20,34)	2649 (2121,3338)	0.2368 (0.0613,0.613)	0.0128 (0.0026,0.0348)	1.1975 (0.2592,3.2091)	32 (9,83)	2 (0,4)	155 (35,418)
Ireland	2.03 (1.25,3.1)	0 (0,0)	1.12 (0.75,1.61)	20 (14,29)	0 (0,0)	10 (7,14)	0.0293 (0.0054,0.091)	0.0001 (0,0.0003)	0.0194 (0.0035,0.0574)	0 (0,1)	0 (0,0)	0 (0,1)
Israel	1.23 (0.77,1.86)	0 (0,0)	0.88 (0.61,1.24)	32 (23,44)	0 (0,0)	23 (16,31)	0.0652 (0.0154,0.1847)	0.0003 (0,0.0008)	0.059 (0.0144,0.1493)	2 (0,5)	0 (0,0)	2 (0,4)
Italy	2.52 (1.56,3.84)	0.01 (0,0.01)	1.49 (1.02,2.1)	194 (134,276)	0 (0,0)	102 (73,138)	0.068 (0.017,0.1772)	0.0003(0,0.0007)	0.0485 (0.0125,0.122)	5 (1,14)	0 (0,0)	3 (1,9)
Jamaica	1.78 (1.17,2.59)	0.03 (0.02,0.04)	3.33 (2.54,4.26)	10 (8,13)	0 (0,0)	18 (14,23)	0.0275 (0.0039,0.1087)	0.0009(0.0001,0.0034)	0.0897 (0.0121,0.3425)	0 (0,1)	0 (0,0)	0 (0,2)
Japan	0.46 (0.27,0.72)	0.01 (0.01,0.01)	0.96 (0.78,1.22)	70 (48,100)	1 (1,1)	137 (113,166)	0.0034 (0.0004,0.0129)	0.0001(0,0.0004)	0.012 (0.0013,0.0468)	1 (0,2)	0 (0,0)	2 (0,6)
Jordan	2.13 (1.26,3.29)	0.05 (0.03,0.06)	4.74 (3.6,6.2)	80 (54,113)	2 (1,2)	163 (131,204)	0.0828 (0.0127,0.3052)	0.0038 (0.0005,0.0144)	0.3493 (0.0494,1.3403)	3 (0,11)	0 (0,1)	12 (2,47)
Kazakhstan	8.92 (6.05,12.63)	0.36 (0.29,0.46)	36.53 (29.19,45.9)	483 (369,635)	20 (16,25)	2017 (1664,2465)	1.8569 (0.7893,3.5424)	0.1038 (0.0427,0.1777)	10.0571 (4.3068,17.0102)	101 (45,182)	6 (2,10)	556 (252,926)
Kenya	46.78 (29.75,68.35)	5.15 (2.88,7.56)	480.15 (281.25,689.92)	8705 (6450,11565)	903 (500,1326)	84004 (48081,119547)	0.8235 (0.1961,2.1115)	0.1908 (0.0306,0.5815)	17.1235 (2.7914,51.7331)	152 (39,363)	33 (5,103)	2993 (488,9167)
Kiribati	129.03	9.99	867.18	54 (41,72)	4 (3,6)	361	0.3597	0.0589	5.0424	0 (0,1)	0 (0,0)	2 (0,9)

Country and territory	All-form TB						MDR-TB					
	ASIR	ASMR	ASDR	Incident cases	Deaths	DALYs	ASIR	ASMR	ASDR	Incident cases	Deaths	DALYs
	(85.44,179.96)	(6.48,14.98)	(566.29,1294.7)			(259,515)	(0.0362,1.4828)	(0.0047,0.251)	(0.4025,21.5848)			
Kuwait	4.47 (2.89,6.54)	0.08 (0.07,0.1)	8.81 (7.11,10.9)	38 (28,52)	1 (1,1)	73 (61,87)	0.0683 (0.0139,0.202)	0.0029 (0.0005,0.009)	0.2658 (0.0494,0.8179)	1 (0,2)	0 (0,0)	2 (0,7)
Kyrgyzstan	17.48 (11.77,24.64)	0.78 (0.65,0.94)	75.91 (62.47,90.75)	395 (301,525)	18 (15,21)	1725 (1465,2024)	4.3171 (1.3329,9.2527)	0.2415 (0.0832,0.4341)	22.8224 (8.0277,40.6458)	98 (30,203)	5 (2,10)	518 (184,927)
Lao People's Democratic Republic	32.66 (22.01,46.75)	4.45 (2.65,6.83)	399.72 (244.48,607.25)	752 (583,973)	104 (64,156)	9344 (5869,13871)	0.1568 (0.0207,0.6579)	0.0421 (0.0038,0.1727)	3.7022 (0.3369,15.0993)	4 (0,16)	1 (0,4)	87 (8,354)
Latvia	5.4 (3.48,8.02)	0.04 (0.04,0.05)	6.35 (4.66,8.39)	16 (12,22)	0 (0,0)	18 (14,22)	0.563 (0.2057,1.2416)	0.0075 (0.0026,0.0149)	0.9019 (0.3428,1.7772)	2 (1,4)	0 (0,0)	3 (1,5)
Lebanon	3.34 (2.18,4.81)	0.03 (0.02,0.05)	4.43 (3.14,6.13)	43 (32,57)	0 (0,1)	56 (43,71)	0.0648 (0.0074,0.2584)	0.0015 (0.0002,0.006)	0.1525 (0.017,0.6129)	1 (0,3)	0 (0,0)	2 (0,7)
Lesotho	270.5 (158.81,406.36)	22.99 (14.2,34.1)	2176.31 (1373.35,3150.19)	1706 (1213,2377)	139 (88,199)	13170 (8650,18100)	13.5069 (3.307,36.4884)	2.3926 (0.5165,7.0696)	215.1922 (46.6492,624.8916)	85 (21,232)	15 (3,43)	1302 (290,3757)
Liberia	46.9 (32.77,64.67)	3.37 (1.8,6.8)	331.51 (196.33,635.28)	1027 (797,1305)	74 (42,146)	7251 (4515,13682)	0.8258 (0.1185,2.8914)	0.1369 (0.0155,0.5025)	12.5761 (1.468,46.2571)	18 (3,64)	3 (0,11)	275 (32,986)
Libya	10.96 (7.19,15.8)	0.44 (0.26,0.68)	40.4 (24.58,60.88)	165 (122,216)	6 (3,9)	520 (329,766)	0.2001 (0.0208,0.7435)	0.0172 (0.0016,0.0671)	1.5301 (0.1402,5.9612)	3 (0,11)	0 (0,1)	20 (2,77)
Lithuania	7.85 (5.12,11.46)	0.06 (0.05,0.07)	8.03 (6.19,10.66)	32 (24,45)	0 (0,0)	31 (25,39)	1.4272 (0.6029,2.7918)	0.0149 (0.0062,0.0261)	1.8057 (0.8149,3.1857)	6 (2,11)	0 (0,0)	7 (3,12)
Luxembourg	4.21 (2.55,6.55	0 (0,0)	1.06 (0.66,1.61)	4 (3,6)	0 (0,0)	1 (1,1)	0.0916 (0.0109,0.3254)	0.0001 (0,0.0003)	0.0258 (0.0034,0.08	0 (0,0)	0 (0,0)	0 (0,0)

Country and territory	All-form TB						MDR-TB					
	ASIR	ASMR	ASDR	Incident cases	Deaths	DALYs	ASIR	ASMR	ASDR	Incident cases	Deaths	DALYs
	)								75)			
Madagascar	62.87 (45.65,85.93)	7.89 (4.88,12.48)	754.39 (491.85,1156.87)	7358 (5841,9171)	922 (593,1422)	88253 (60306,131917)	1.626 (0.2609,5.5276)	0.4635 (0.0597,1.6647)	42.1257 (5.5901,150.6287)	190 (31,617)	54 (7,193)	4927 (669,17532)
Malawi	111.64 (70.11,163.95)	6.83 (4.18,10.93)	681.31 (437.23,1050.33)	9033 (6625,12152)	537 (341,836)	53547 (35436,79333)	2.5477 (0.481,7.7993)	0.3462 (0.0556,1.1761)	32.0725 (5.452,108.8592)	206 (43,630)	27 (4,91)	2519 (431,8431)
Malaysia	11.33 (7.55,16.43)	0.19 (0.15,0.25)	22.2 (16.39,29.14)	870 (656,1127)	15 (12,17)	1664 (1358,2029)	0.0966 (0.021,0.3017)	0.0036 (0.0006,0.0112)	0.3481 (0.0682,1.1282)	7 (2,23)	0 (0,1)	26 (5,85)
Maldives	13.27 (8.94,18.92)	0.4 (0.28,0.57)	40.92 (29.36,56.44)	13 (10,17)	0 (0,1)	39 (31,50)	0.0514 (0.0059,0.1992)	0.0034 (0.0003,0.0145)	0.3149 (0.0316,1.3344)	0 (0,0)	0 (0,0)	0 (0,1)
Mali	52.42 (36.33,73.34)	4.63 (2.76,8.54)	446.3 (281.52,785.94)	6343 (5028,8008)	588 (355,1092)	56658 (36898,100125)	1.36 (0.2032,4.2356)	0.2703 (0.0313,0.899)	24.6352 (2.9998,80.29)	165 (26,481)	34 (4,112)	3133 (383,9945)
Malta	10.89 (6.76,16.95)	0 (0,0)	1.28 (0.8,1.94)	7 (5,10)	0 (0,0)	1 (1,1)	0.0718 (0.0083,0.2931)	0 (0,0.0001)	0.0094 (0.0011,0.0374)	0 (0,0)	0 (0,0)	0 (0,0)
Marshall Islands	127.54 (84.34,181.32)	5.2 (3.14,7.43)	444.49 (273.41,632.26)	23 (17,30)	1 (1,1)	77 (48,105)	1.2054 (0.1497,4.8951)	0.1055 (0.0103,0.4956)	8.9121 (0.8706,41.8246)	0 (0,1)	0 (0,0)	2 (0,7)
Mauritania	26.01 (18.65,35.38)	1.68 (1.09,2.92)	171.06 (117.24,277.55)	484 (385,609)	31 (22,53)	3199 (2282,5077)	0.5967 (0.0912,1.8849)	0.0898 (0.0115,0.3291)	8.3546 (1.1149,29.9876)	11 (2,34)	2 (0,6)	156 (22,550)
Mauritius	4.32 (2.83,6.33)	0.04 (0.03,0.04)	6.22 (4.03,9.46)	9 (7,12)	0 (0,0)	12 (9,17)	0.0164 (0.003,0.0519)	0.0003 (0,0.001)	0.0374 (0.0061,0.1275)	0 (0,0)	0 (0,0)	0 (0,0)
Mexico	3.36 (2.14,5.05)	0.15 (0.11,0.18)	13.71 (10.85,17.24)	1105 (775,1499)	45 (36,55)	4229 (3435,5246)	0.1006 (0.0191,0.317)	0.0085 (0.0015,0.0268)	0.7597 (0.132,2.4041)	33 (7,105)	3 (0,8)	234 (42,738)
Micronesia	26.32	0.75	70.63	8 (6,11)	0 (0,0)	21 (16,28)	0.0733	0.0046	0.4064	0 (0,0)	0 (0,0)	0 (0,1)

Country and territory	All-form TB						MDR-TB					
	ASIR	ASMR	ASDR	Incident cases	Deaths	DALYs	ASIR	ASMR	ASDR	Incident cases	Deaths	DALYs
(Federated States of)	(17.69,37.55)	(0.49,1.09)	(47.77,99.02)				(0.0066,0.3374)	(0.0004,0.0213)	(0.0312,1.8485)			
Monaco	1.12 (0.72,1.67)	0.01 (0,0.01)	1.13 (0.82,1.54)	0 (0,0)	0 (0,0)	0 (0,0)	0.0137 (0.0018,0.0529)	0.0002(0,0.0006)	0.0202 (0.0025,0.0743)	0 (0,0)	0 (0,0)	0 (0,0)
Mongolia	21.47 (14.59,29.99)	1.63 (1.07,2.49)	148.43 (98.17,222.98)	231 (181,292)	18 (12,27)	1641 (1146,2425)	2.0542 (0.4674,5.5428)	0.2449 (0.0496,0.5939)	21.9204 (4.4888,52.7755)	22 (5,57)	3 (1,6)	242 (50,580)
Montenegro	1.9 (1.23,2.78)	0.02 (0.01,0.03)	2.1 (1.51,2.87)	2 (2,3)	0 (0,0)	2 (2,3)	0.0106 (0.0015,0.0384)	0.0002(0,0.0009)	0.0202 (0.0026,0.0863)	0 (0,0)	0 (0,0)	0 (0,0)
Morocco	24.37 (16.1,35.32)	0.69 (0.44,1.11)	62.46 (40.95,98.82)	2407 (1782,3277)	66 (45,103)	6005 (4143,9048)	0.5185 (0.126,1.3885)	0.0325 (0.006,0.0933)	2.8447 (0.5277,8.0441)	51 (13,135)	3 (1,9)	274 (50,771)
Mozambique	125.48 (76.34,184.69)	14.21 (9.43,19.93)	1337.45 (915.42,1843.65)	17945 (12953,23704)	2088 (1416,2878)	196617 (138905,266114)	7.1971 (1.8609,18.9412)	1.7242 (0.3785,4.6671)	156.0821 (35.0475,417.5505)	1031 (298,2627)	253 (56,685)	22952 (5194,61156)
Myanmar	47.19 (31.51,67.21)	4.33 (2.67,6.43)	395.77 (252.64,576.05)	7386 (5618,9400)	659 (427,935)	60143 (39908,83828)	2.5393 (0.7381,6.2474)	0.4593 (0.1051,1.274)	40.8506 (9.5456,112.5165)	397 (125,1000)	70 (17,192)	6206 (1553,17151)
Namibia	141.12 (92.59,204.59)	7.31 (4.66,11.27)	736.66 (499.42,1075.39)	1162 (885,1506)	59 (40,90)	5952 (4126,8552)	9.0426 (2.7606,20.6943)	0.9788 (0.2616,2.6117)	90.8982 (26.4146,233.6877)	74 (25,159)	8 (2,20)	734 (214,1823)
Nauru	56.89 (38.06,80.95)	2.71 (1.68,3.91)	237.95 (150.07,338.89)	2 (2,3)	0 (0,0)	9 (6,13)	0.1601 (0.0148,0.756)	0.0172 (0.0012,0.0851)	1.4761 (0.1026,7.3106)	0 (0,0)	0 (0,0)	0 (0,0)
Nepal	27.9 (19.08,39.1)	1.29 (0.82,1.86)	132.22 (88.43,184.18)	2587 (1978,3332)	118 (82,156)	12045 (8848,15673)	1.3704 (0.3138,3.654)	0.1344 (0.0249,0.3865)	12.5598 (2.4881,35.1636)	127 (29,332)	12 (2,33)	1144 (231,3031)
Netherlands	1.59 (1,2.39)	0 (0,0)	0.92 (0.64,1.31)	43 (31,59)	0 (0,0)	24 (17,32)	0.0307 (0.0086,0.0763)	0.0001 (0,0.0004)	0.0231 (0.0062,0.056)	1 (0,2)	0 (0,0)	1 (0,1)

Country and territory	All-form TB						MDR-TB					
	ASIR	ASMR	ASDR	Incident cases	Deaths	DALYs	ASIR	ASMR	ASDR	Incident cases	Deaths	DALYs
New Zealand	2.87 (1.82,4.36)	0 (0,0)	2.68 (1.47,4.39)	28 (20,39)	0 (0,0)	25 (15,39)	0.0468 (0.0088,0.1537)	0.0001(0,0.0005)	0.0489 (0.0087,0.1683)	0 (0,2)	0 (0,0)	0 (0,2)
Nicaragua	6.46 (4.34,9.16)	0.35 (0.24,0.49)	32.1 (22.69,44.02)	128 (98,161)	7 (5,9)	617 (466,800)	0.1217 (0.0195,0.4225)	0.0138 (0.0018,0.0491)	1.2179 (0.1586,4.2927)	2 (0,8)	0 (0,1)	23 (3,82)
Niger	74.01 (52.36,100.62)	11.56 (6.61,22.85)	1062.93 (636.31,2050.16)	9860 (7861,12287)	1636 (956,3162)	150574 (92688,286860)	1.731 (0.2877,5.1342)	0.6061 (0.0753,2.0634)	54.0883 (6.7519,182.1933)	231 (40,642)	86 (11,288)	7668 (952,25617)
Nigeria	58.88 (41.65,80.28)	11.01 (6.4,16.7)	1004.43 (600.32,1497.02)	60664 (48428,75519)	11585 (6755,17448)	1056982 (632245,1564775)	2.547 (0.6061,7.3804)	0.8726 (0.1582,2.8567)	77.7958 (14.2304,252.2259)	2631 (669,7649)	918 (167,2992)	81896 (15153,264655)
Niue	12.72 (8.55,18.24)	0.54 (0.38,0.73)	50.8 (36.71,67)	0 (0,0)	0 (0,0)	0 (0,0)	0.0371 (0.0037,0.1583)	0.0034 (0.0003,0.0163)	0.2995 (0.0262,1.4312)	0 (0,0)	0 (0,0)	0 (0,0)
North Macedonia	2.39 (1.6,3.46)	0.04 (0.03,0.05)	4.44 (3.23,5.96)	8 (6,11)	0 (0,0)	14 (11,17)	0.0269 (0.0064,0.0743)	0.0008(0.0002,0.0024)	0.0851 (0.0167,0.2416)	0 (0,0)	0 (0,0)	0 (0,1)
Northern Mariana Islands	26.06 (17.23,37.5)	0.27 (0.19,0.37)	25.65 (18.69,34.47)	3 (2,4)	0 (0,0)	3 (2,3)	0.1972 (0.0244,0.7542)	0.0042 (0.0004,0.0167)	0.3789 (0.0382,1.505)	0 (0,0)	0 (0,0)	0 (0,0)
Norway	1.68 (1,2.69)	0 (0,0)	0.7 (0.45,1.04)	16 (11,23)	0 (0,0)	6 (4,9)	0.0385 (0.0083,0.113)	0.0001 (0,0.0002)	0.0173 (0.0039,0.0463)	0 (0,1)	0 (0,0)	0 (0,0)
Oman	3.22 (2.08,4.69)	0.05 (0.03,0.07)	5.14 (3.85,7.08)	39 (28,52)	1 (0,1)	63 (49,81)	0.06 (0.0101,0.1966)	0.0019 (0.0003,0.0069)	0.1886 (0.0266,0.6474)	1 (0,2)	0 (0,0)	2 (0,8)
Pakistan	60.57 (41.42,84.83)	7.78 (5.56,10.37)	711.4 (519.85,940.61)	51721 (39501,67873)	6628 (4942,8489)	605979 (460452,772206)	3.7137 (1.0076,9.6201)	0.913 (0.2034,2.5302)	81.0287 (18.3926,222.4974)	3170 (880,8084)	778 (176,2111)	69014 (16030,188064)
Palau	11.28 (7.67,16)	0.38 (0.27,0.5	37.06 (27.4,49.31)	0 (0,0)	0 (0,0)	1 (1,1)	0.032 (0.0028,0.1439)	0.0023 (0.0002,0.0108	0.2087 (0.0173,0.96	0 (0,0)	0 (0,0)	0 (0,0)

Country and territory	All-form TB						MDR-TB					
	ASIR	ASMR	ASDR	Incident cases	Deaths	DALYs	ASIR	ASMR	ASDR	Incident cases	Deaths	DALYs
		2)						)	35)			
Palestine	3.43 (2.26,4.99)	0.08 (0.05,0.13)	8.43 (5.91,12.66)	64 (47,85)	1 (1,2)	153 (113,224)	0.0685 (0.0082,0.2748)	0.0035 (0.0004,0.0141)	0.3363 (0.0367,1.3257)	1 (0,5)	0 (0,0)	6 (1,24)
Panama	8.24 (5.59,11.74)	0.36 (0.27,0.46)	33.98 (26.28,43.69)	95 (72,121)	4 (3,5)	377 (303,467)	0.1741 (0.026,0.5676)	0.0157 (0.0021,0.0537)	1.4108 (0.1953,4.8894)	2 (0,6)	0 (0,1)	16 (2,54)
Papua New Guinea	47.52 (31.45,66.34)	6.36 (3.58,9.66)	563.67 (320.1,850)	1851 (1386,2389)	264 (154,389)	23445 (14007,34396)	1.852 (0.502,4.4937)	0.4566 (0.0956,1.2592)	39.926 (8.4882,109.6318)	73 (20,165)	19 (4,53)	1661 (347,4609)
Paraguay	6.43 (4.36,9.09)	0.5 (0.33,0.72)	47.57 (32.56,67.16)	130 (98,168)	10 (7,13)	908 (645,1254)	0.2133 (0.0277,0.7216)	0.0317 (0.0036,0.1072)	2.8792 (0.338,9.6599)	4 (1,14)	1 (0,2)	55 (7,177)
Peru	20.06 (13.29,28.7)	0.74 (0.48,1.08)	70.55 (48.44,100.18)	1919 (1431,2591)	70 (50,95)	6704 (4936,8830)	1.6145 (0.5942,3.759)	0.1126 (0.0328,0.2647)	10.1587 (3.0852,23.6267)	154 (59,339)	11 (3,25)	965 (308,2182)
Philippines	119.82 (81.61,167.74)	2.85 (2.28,3.49)	315.51 (253.66,386.49)	40913 (31119,53045)	949 (779,1133)	104997 (87796,123799)	3.1344 (0.7918,8.1555)	0.1483 (0.0326,0.4148)	14.4726 (3.3743,39.2185)	1070 (269,2772)	49 (11,137)	4816 (1127,12978)
Poland	2.03 (1.3,3.05)	0 (0,0)	1.29 (0.83,1.9)	120 (86,161)	0 (0,0)	72 (49,104)	0.0167 (0.0043,0.0463)	0 (0,0.0001)	0.0121 (0.003,0.0328)	1 (0,3)	0 (0,0)	1 (0,2)
Portugal	3.16 (1.99,4.76)	0.01 (0.01,0.01)	2.06 (1.55,2.74)	44 (31,61)	0 (0,0)	26 (20,33)	0.0467 (0.0078,0.1492)	0.0004 (0,0.0011)	0.0459 (0.0072,0.1365)	1 (0,2)	0 (0,0)	1 (0,2)
Puerto Rico	1.17 (0.73,1.77)	0.01 (0.01,0.01)	1.46 (1.14,1.89)	5 (4,7)	0 (0,0)	5 (4,7)	0.0113 (0.002,0.0364)	0.0002 (0,0.0007)	0.0228 (0.0037,0.0748)	0 (0,0)	0 (0,0)	0 (0,0)
Qatar	9.11 (5.86,13.56)	0.08 (0.06,0.12)	9.02 (6.57,12.31)	44 (33,60)	0 (0,1)	46 (35,59)	0.1813 (0.0329,0.5653)	0.0038 (0.0005,0.0132)	0.3579 (0.055,1.224)	1 (0,3)	0 (0,0)	2 (0,6)
Republic	1.69	0.04	4.17	105 (76,141)	2 (2,3)	233	0.0209	0.0009	0.0901	1 (0,5)	0 (0,0)	5 (1,20)

Country and territory	All-form TB						MDR-TB					
	ASIR	ASMR	ASDR	Incident cases	Deaths	DALYs	ASIR	ASMR	ASDR	Incident cases	Deaths	DALYs
of Korea	(1.09,2.49)	(0.03,0.05)	(3.24,5.3)			(196,277)	(0.0028,0.0774)	(0.0001,0.0037)	(0.0111,0.3546)			
Republic of Moldova	12.06 (7.94,17.46)	0.16 (0.12,0.21)	17.72 (13.46,23.17)	65 (47,88)	1 (1,1)	82 (65,102)	4.8434 (2.5283,8.1724)	0.0688 (0.0391,0.1053)	7.5465 (4.4352,11.562)	26 (14,43)	0 (0,0)	35 (21,52)
Romania	11.15 (7.45,15.83)	0.38 (0.31,0.45)	37.15 (29.87,45.85)	342 (258,453)	10 (8,12)	1008 (835,1199)	0.3411 (0.0965,0.8056)	0.0201 (0.0049,0.0508)	1.9002 (0.4883,4.76)	10 (3,23)	1 (0,1)	51 (13,129)
Russian Federation	10 (6.24,14.89)	0.1 (0.09,0.11)	11.71 (9.98,13.66)	2702 (1872,3792)	23 (21,25)	2745 (2398,3168)	2.7964 (1.2837,5.1258)	0.0308 (0.0155,0.0476)	3.3536 (1.6876,5.1541)	756 (371,1363)	7 (4,11)	783 (412,1191)
Rwanda	43.68 (29.38,61.15)	4.21 (2.71,6.25)	402.13 (265.79,580.7)	2176 (1667,2755)	210 (137,307)	20058 (13578,28387)	1.4046 (0.4381,3.3103)	0.3077 (0.0674,0.7822)	27.946 (6.3766,69.853)	70 (23,161)	15 (3,38)	1394 (322,3385)
Saint Kitts and Nevis	3.14 (2.13,4.41)	0.08 (0.06,0.11)	8.89 (6.61,11.62)	0 (0,0)	0 (0,0)	1 (1,1)	0.0138 (0.0019,0.053)	0.0001(0.0001,0.0032)	0.0736 (0.0088,0.2945)	0 (0,0)	0 (0,0)	0 (0,0)
Saint Lucia	3.86 (2.54,5.73)	0.11 (0.08,0.15)	11.17 (8.26,14.79)	1 (1,2)	0 (0,0)	3 (3,4)	0.0161 (0.0023,0.0623)	0.001 (0.0001,0.0042)	0.0891 (0.013,0.3758)	0 (0,0)	0 (0,0)	0 (0,0)
Saint Vincent and the Grenadines	5.92 (3.83,8.64)	0.08 (0.06,0.1)	9.5 (7.22,12.36)	1 (1,2)	0 (0,0)	2 (2,3)	0.0316 (0.0041,0.1178)	0.0008 (0.0001,0.003)	0.0817 (0.0102,0.3144)	0 (0,0)	0 (0,0)	0 (0,0)
Samoa	14.35 (9.58,20.65)	0.47 (0.29,0.73)	44.98 (29.28,66.77)	11 (9,15)	0 (0,1)	36 (25,52)	0.0088 (0.001,0.0334)	0.0006 (0.0001,0.0031)	0.0566 (0.005,0.2766)	0 (0,0)	0 (0,0)	0 (0,0)
San Marino	0.6 (0.39,0.89)	0 (0,0.01)	0.67 (0.45,0.94)	0 (0,0)	0 (0,0)	0 (0,0)	0.0073 (0.0011,0.0262)	0.0001(0,0.0003)	0.0116 (0.0018,0.045)	0 (0,0)	0 (0,0)	0 (0,0)
Sao Tome and	19.13 (13.88,25.	0.41 (0.24,0.9	53.92 (34.69,99.5	14 (11,18)	0 (0,1)	40 (28,70)	0.6661 (0.094,2.0696)	0.0306 (0.0033,0.1044	3.237 (0.3932,10.4	1 (0,2)	0 (0,0)	2 (0,8)

Country and territory	All-form TB						MDR-TB					
	ASIR	ASMR	ASDR	Incident cases	Deaths	DALYs	ASIR	ASMR	ASDR	Incident cases	Deaths	DALYs
Principe	96)	4)	2)					)	572)			
Saudi Arabia	8.55 (5.42,12.56)	0.08 (0.06,0.13)	10.2 (6.96,14.83)	657 (473,895)	6 (4,9)	754 (553,1008)	0.2235 (0.0428,0.7092)	0.0048 (0.0008,0.0156)	0.4855 (0.0855,1.6172)	17 (3,55)	0 (0,1)	36 (6,120)
Senegal	38.58 (27.03,53.52)	2.37 (1.53,4.6)	238.15 (163.74,431.09)	2471 (1925,3131)	152 (102,280)	15327 (10799,26548)	0.8948 (0.22,2.2645)	0.1262 (0.0249,0.4031)	11.6805 (2.4294,37.2381)	57 (14,146)	8 (2,26)	752 (159,2311)
Serbia	2.7 (1.76,3.94)	0.01 (0.01,0.02)	2.12 (1.55,2.85)	37 (27,51)	0 (0,0)	25 (19,32)	0.0237 (0.0052,0.0649)	0.0002 (0,0.0006)	0.0276 (0.0056,0.0806)	0 (0,1)	0 (0,0)	0 (0,1)
Seychelles	6.04 (4.09,8.68)	0.16 (0.12,0.2)	17.54 (12.86,23.77)	1 (1,2)	0 (0,0)	4 (3,5)	0.0114 (0.0012,0.0465)	0.0006 (0,0.0028)	0.0624 (0.0055,0.2726)	0 (0,0)	0 (0,0)	0 (0,0)
Sierra Leone	86.73 (61.71,117.28)	11.57 (7.64,18.49)	1075.06 (733.69,1694.58)	3187 (2565,4001)	437 (297,688)	40650 (28294,62681)	1.9272 (0.3242,6.1636)	0.5685 (0.0793,1.8542)	51.1082 (7.1745,165.6081)	71 (13,225)	21 (3,70)	1934 (271,6222)
Singapore	1.5 (0.92,2.29)	0.03 (0.02,0.03)	2.97 (2.41,3.68)	12 (9,16)	0 (0,0)	24 (20,29)	0.0172 (0.0051,0.0495)	0.0006 (0.0001,0.0018)	0.0619 (0.0167,0.1704)	0 (0,0)	0 (0,0)	1 (0,1)
Slovakia	0.82 (0.55,1.16)	0.02 (0.01,0.02)	2.23 (1.73,2.83)	7 (5,9)	0 (0,0)	18 (15,23)	0.0057 (0.001,0.0179)	0.0003 (0,0.0009)	0.0262 (0.0036,0.0915)	0 (0,0)	0 (0,0)	0 (0,1)
Slovenia	1.5 (0.95,2.26)	0 (0,0)	1.08 (0.76,1.5)	5 (3,6)	0 (0,0)	3 (2,4)	0.0014 (0.0002,0.005)	0 (0,0)	0.0013 (1e-04,0.0047)	0 (0,0)	0 (0,0)	0 (0,0)
Solomon Islands	25.89 (16.95,36.56)	1.33 (0.88,1.99)	121.72 (83.37,176.06)	67 (51,87)	3 (2,5)	320 (236,438)	0.073 (0.0064,0.3087)	0.0082 (0.0007,0.0365)	0.7122 (0.0586,3.1856)	0 (0,1)	0 (0,0)	2 (0,8)
Somalia	110.15 (75.76,153.13)	24.91 (13.05,46.68)	2267.89 (1233.77,4197.18)	11811 (9298,14882)	2877 (1546,5318)	261976 (145420,479605)	8.5557 (1.9552,24.0231)	3.5394 (0.6223,11.1933)	315.9595 (55.9502,993.4033)	930 (220,2584)	409 (74,1276)	36535 (6686,113535)
South	169.33	8.33	845.7	25631	1220	123780	5.1788	0.5529	51.4274	784	81	7519

Country and territory	All-form TB						MDR-TB					
	ASIR	ASMR	ASDR	Incident cases	Deaths	DALYs	ASIR	ASMR	ASDR	Incident cases	Deaths	DALYs
Africa	(112.65,233.12)	(6.47,10.52)	(677.65,1053.51)	(19292,33382)	(977,1508)	(102171,150902)	(1.2916,14.3106)	(0.1173,1.4969)	(11.7113,138.2042)	(193,2126)	(17,216)	(1698,19941)
South Sudan	111.1 (76.98,153.77)	29.19 (19.06,45.74)	2651.95 (1760.67,4112.74)	4842 (3819,6057)	1298 (856,2019)	117807 (79392,181856)	5.5343 (0.9675,17.1207)	2.6787 (0.3924,8.198)	239.0643 (35.5863,731.0185)	242 (46,739)	119 (18,362)	10626 (1613,32253)
Spain	2.08 (1.2,3.27)	0 (0,0.01)	0.96 (0.68,1.35)	141 (93,203)	0 (0,0)	57 (42,77)	0.0277 (0.0035,0.1121)	0.0001 (0,0.0005)	0.0173 (0.0023,0.0688)	2 (0,8)	0 (0,0)	1 (0,4)
Sri Lanka	8.93 (6.02,12.83)	0.15 (0.1,0.21)	17.97 (12.54,25.12)	456 (348,595)	7 (5,10)	887 (675,1132)	0.0306 (0.0035,0.1233)	0.0011 (0.0002,0.0045)	0.1101 (0.0133,0.4499)	2 (0,6)	0 (0,0)	5 (1,21)
Sudan	18.64 (12.48,26.76)	1 (0.61,1.52)	90.06 (56.21,134.14)	3101 (2352,4085)	163 (104,239)	14670 (9661,21212)	0.395 (0.0482,1.5725)	0.0446 (0.005,0.173)	3.9257 (0.4459,15.2898)	66 (9,269)	7 (1,28)	639 (74,2497)
Suriname	6.15 (4.16,8.83)	0.18 (0.12,0.26)	17.8 (12.33,24.7)	9 (7,11)	0 (0,0)	24 (18,31)	0.0232 (0.0033,0.0896)	0.0016 (0.0002,0.0066)	0.1395 (0.0139,0.5879)	0 (0,0)	0 (0,0)	0 (0,1)
Sweden	3.33 (2.07,5.17)	0 (0,0)	1.26 (0.77,1.95)	61 (43,87)	0 (0,0)	22 (14,32)	0.1059 (0.0278,0.2958)	0.0001 (0,0.0002)	0.0417 (0.0118,0.1107)	2 (1,5)	0 (0,0)	1 (0,2)
Switzerland	2.16 (1.36,3.24)	0 (0,0)	1.1 (0.71,1.61)	29 (21,39)	0 (0,0)	14 (10,20)	0.0836 (0.0234,0.2191)	0.0002 (0,0.0005)	0.0482 (0.0143,0.1167)	1 (0,3)	0 (0,0)	1 (0,1)
Syrian Arab Republic	3.94 (2.55,5.75)	0.09 (0.06,0.14)	9.49 (6.43,13.73)	155 (111,215)	3 (2,5)	313 (228,432)	0.0971 (0.0116,0.3736)	0.0051 (0.0004,0.0204)	0.4757 (0.044,1.8502)	4 (0,15)	0 (0,1)	16 (1,59)
Taiwan (Province of China)	11.57 (7.89,16.69)	0.07 (0.06,0.09)	12.6 (8.78,17.7)	333 (247,448)	2 (2,2)	345 (259,457)	0.2294 (0.0318,0.8456)	0.003 (0.0004,0.0113)	0.3822 (0.0518,1.3838)	7 (1,26)	0 (0,0)	10 (1,38)
Tajikistan	17.12 (12.01,23.97)	3.52 (2.19,5.28)	315.92 (198.78,473.21)	621 (479,780)	133 (86,197)	11986 (7733,17709)	2.6776 (0.9842,5.3151)	0.7549 (0.2466,1.5043)	67.401 (22.0484,134.0228)	97 (36,182)	29 (9,56)	2558 (838,4986)

Country and territory	All-form TB						MDR-TB					
	ASIR	ASMR	ASDR	Incident cases	Deaths	DALYs	ASIR	ASMR	ASDR	Incident cases	Deaths	DALYs
Thailand	32.69 (21.55,47.01)	0.51 (0.39,0.65)	52.82 (40.46,67.3)	3348 (2392,4461)	48 (39,58)	4926 (3992,5962)	0.7884 (0.1761,2.1567)	0.0247 (0.0043,0.0707)	2.3145 (0.4215,6.611)	81 (18,221)	2 (0,6)	217 (39,598)
Timor-Leste	95.99 (65.43,133.64)	8.89 (5.88,12.73)	793.03 (530.81,1127.49)	502 (389,639)	47 (33,65)	4164 (2930,5755)	0.4368 (0.0537,1.7302)	0.0878 (0.0072,0.3653)	7.698 (0.6388,31.9298)	2 (0,9)	0 (0,2)	40 (3,165)
Togo	66.1 (46.32,92.38)	4.59 (2.65,8.7)	448.66 (279.66,801.78)	2191 (1726,2742)	153 (97,279)	14979 (10034,26023)	1.7606 (0.246,5.9744)	0.269 (0.0312,0.9381)	24.6495 (3.0427,87.4686)	58 (8,180)	9 (1,32)	823 (105,2908)
Tokelau	9.95 (6.58,14.4)	0.28 (0.2,0.39)	27.56 (19.57,36.63)	0 (0,0)	0 (0,0)	0 (0,0)	0.0259 (0.0027,0.0995)	0.0016 (0.0001,0.0076)	0.1509 (0.0118,0.6724)	0 (0,0)	0 (0,0)	0 (0,0)
Tonga	10.77 (7.12,15.56)	0.35 (0.24,0.5)	33.69 (23.62,46.17)	4 (3,5)	0 (0,0)	13 (10,17)	0.0307 (0.0031,0.1402)	0.0021 (0.0002,0.0107)	0.1913 (0.0181,0.9743)	0 (0,0)	0 (0,0)	0 (0,0)
Trinidad and Tobago	3.31 (2.22,4.8)	0.06 (0.04,0.08)	6.67 (4.77,8.85)	9 (7,12)	0 (0,0)	17 (13,22)	0.0161 (0.002,0.0593)	0.0005 (0.0001,0.0024)	0.0551 (0.0066,0.2259)	0 (0,0)	0 (0,0)	0 (0,1)
Tunisia	7.99 (5.31,11.62)	0.11 (0.07,0.18)	10.67 (6.96,16.84)	220 (162,287)	3 (2,5)	285 (199,429)	0.1067 (0.0285,0.2878)	0.0034 (0.0007,0.0109)	0.3073 (0.0629,0.9685)	3 (1,8)	0 (0,0)	8 (2,26)
Türkiye	12 (7.83,17.27)	1.44 (1.13,1.81)	133.37 (106.29,167.13)	183 (136,238)	22 (18,28)	2052 (1657,2526)	2.0509 (0.6073,4.6918)	0.3316 (0.1012,0.6539)	30.2439 (9.3495,59.4213)	31 (10,70)	5 (2,10)	465 (145,907)
Turkmenistan	75.18 (50.79,109.64)	2.77 (1.84,3.83)	240.49 (161.24,331.51)	3 (2,4)	0 (0,0)	9 (6,12)	0.2168 (0.0199,0.9953)	0.017 (0.0012,0.08)	1.4534 (0.1015,6.8674)	0 (0,0)	0 (0,0)	0 (0,0)
Tuvalu	3.81 (2.5,5.49)	0.06 (0.05,0.09)	7.15 (5.27,9.61)	712 (530,942)	11 (8,14)	1233 (971,1588)	0.151 (0.0491,0.3494)	0.0054 (0.0014,0.0133)	0.5323 (0.1485,1.2743)	28 (10,63)	1 (0,2)	92 (26,212)
Uganda	78.43 (52.27,11	6.93 (4.01,11.	673.05 (414.66,107	15787 (12156,20454)	1435 (859,234	139247 (88654,218	2.6467 (0.6216,7.2655)	0.5125 (0.097,1.6295)	47.0494 (9.2805,146.	532 (131,1373)	106 (21,33	9739 (1942,301

Country and territory	All-form TB						MDR-TB					
	ASIR	ASMR	ASDR	Incident cases	Deaths	DALYs	ASIR	ASMR	ASDR	Incident cases	Deaths	DALYs
	0.96)	5)	3.7)		5)	372)			7093)		5)	82)
Ukraine	8.41 (5.13,12.84)	0.13 (0.1,0.16)	14.25 (11.25,17.52)	562 (382,801)	7 (5,8)	733 (600,874)	2.6048 (1.04,5.1)	0.0468 (0.0216,0.0752)	4.865 (2.2056,7.8077)	174 (77,324)	2 (1,4)	249 (114,394)
United Arab Emirates	1.83 (1.22,2.59)	0.02 (0.01,0.05)	3.2 (1.85,5.46)	24 (19,30)	0 (0,1)	42 (25,70)	0.0356 (0.0045,0.1441)	0.0009 (0.0001,0.004)	0.1036 (0.0107,0.4529)	0 (0,2)	0 (0,0)	1 (0,6)
United Kingdom	4.28 (2.61,6.7)	0.01 (0,0.01)	1.6 (1.1,2.29)	522 (352,759)	1 (1,1)	178 (127,242)	0.054 (0.0124,0.1531)	0.0002 (0,0.0004)	0.0256 (0.006,0.0677)	7 (2,19)	0 (0,0)	3 (1,8)
United Republic of Tanzania	58.09 (38.89,82.62)	6.9 (4.34,9.9)	655.11 (429.86,927.5)	14307 (11166,18619)	1733 (1108,2453)	164531 (111204,228515)	1.5611 (0.2496,5.4599)	0.4191 (0.0588,1.5927)	38.0864 (5.4429,143.6045)	385 (65,1335)	105 (15,393)	9571 (1366,35792)
United States of America	1.3 (0.88,1.88)	0.02 (0.01,0.03)	2.44 (1.61,3.58)	431 (316,569)	4 (3,4)	664 (520,848)	0.0111 (0.0033,0.0298)	0.0004(0,0.0006)	0.0254 (0.0075,0.0698)	6 (2,16)	0 (0,0)	14 (4,38)
United States Virgin Islands	0.74 (0.48,1.1)	0.01 (0.01,0.01)	1.19 (0.9,1.58)	0 (0,0)	0 (0,0)	0 (0,0)	0.0063 (9e-04,0.0246)	0.0002 (0,0.0008)	0.0205 (0.0024,0.0811)	0 (0,0)	0 (0,0)	0 (0,0)
Uruguay	6.41 (4.04,9.42)	0.06 (0.05,0.07)	6.81 (5.23,8.76)	43 (31,59)	0 (0,0)	42 (34,51)	0.0197 (0.0034,0.0661)	0.0004(0,0.0013)	0.0387 (0.0062,0.1253)	0 (0,0)	0 (0,0)	0 (0,1)
Uzbekistan	17.65 (11.95,24.99)	0.97 (0.78,1.18)	91.3 (74.83,111.97)	1753 (1337,2274)	103 (85,125)	9765 (8149,11645)	4.3201 (1.3605,9.1633)	0.2928 (0.0994,0.5216)	27.1377 (9.1782,47.7992)	429 (143,877)	31 (11,56)	2904 (984,5079)
Vanuatu	22.19 (14.79,31.26)	1.15 (0.7,1.76)	104.31 (65.92,154.95)	26 (19,34)	1 (1,2)	122 (83,177)	0.0395 (0.0044,0.1684)	0.0046 (0.0004,0.0204)	0.3945 (0.0343,1.7668)	0 (0,0)	0 (0,0)	0 (0,2)
Venezuela (Bolivarian	5.06 (3.48,7.15	0.24 (0.17,0.3	22.87 (16.58,31)	336 (261,432)	16 (12,21)	1481 (1111,1921)	0.0943 (0.0142,0.3457)	0.0094 (0.0012,0.0357	0.8411 (0.1085,3.19	6 (1,23)	1 (0,2)	54 (7,204)

Country and territory	All-form TB						MDR-TB					
	ASIR	ASMR	ASDR	Incident cases	Deaths	DALYs	ASIR	ASMR	ASDR	Incident cases	Deaths	DALYs
Republic of)	)	3)						)	2)			
Viet Nam	25.91 (17.93,35.9)	0.76 (0.51,1.08)	79.94 (57.36,108.88)	6352 (4931,8021)	182 (134,247)	19201 (14763,24557)	0.8628 (0.2081,2.2754)	0.0516 (0.0091,0.1453)	4.8983 (0.9617,13.2022)	212 (53,544)	12 (2,34)	1176 (236,3109)
Yemen	8.33 (5.71,11.84)	0.81 (0.49,1.24)	74.45 (46.75,110.99)	1148 (891,1494)	110 (69,166)	10091 (6491,14936)	0.1899 (0.0346,0.6864)	0.0397 (0.0062,0.1438)	3.5426 (0.5573,12.7319)	26 (5,97)	5 (1,19)	480 (77,1687)
Zambia	95.42 (58.86,140.61)	5.42 (3.11,8.83)	544.13 (335.88,860.06)	7889 (5699,10477)	452 (260,726)	45422 (28555,69731)	3.7848 (0.548,12.1715)	0.4448 (0.0603,1.4821)	41.5459 (6.0085,135.59)	313 (49,1014)	37 (5,123)	3468 (511,11312)
Zimbabwe	148.73 (92.18,220.95)	16.96 (10.87,24.22)	1573.15 (1038.67,2190.69)	9362 (6806,12706)	1070 (729,1472)	99235 (69477,135127)	7.0355 (1.0978,23.7544)	1.6662 (0.2498,5.307)	149.0453 (22.8209,475.2362)	443 (72,1444)	105 (16,332)	9402 (1461,29815)

Note: Data are presented as estimates with 95% uncertainty intervals (UIs) in parentheses. Estimates are provided for 204 countries and territories included in the Global Burden of Diseases, Injuries, and Risk Factors Study (GBD) 2021. All-form TB includes all clinical forms of tuberculosis (drug-susceptible and drug-resistant strains). Abbreviations: TB, tuberculosis; MDR-TB, multidrug-resistant tuberculosis; ASIR, age-standardized incidence rate (per 100,000 population); ASMR, age-standardized mortality rate (per 100,000 population); ASDR, age-standardized DALY rate (per 100,000 population); DALYs, disability-adjusted life years.

Table S3. Age-specific burden and mortality-to-incidence ratio of pediatric all-form TB and MDR-TB globally and by region in 2021.

Disease	Location	Age group	Incident cases	Deaths	MIR (%)
MDR-TB	Global	<5 years	13988 (6013, 28693)	4544 (1382, 11177)	32.48 (8.81, 119.79)
MDR-TB	Global	5-9 years	7095 (2726, 15838)	667 (210, 1554)	9.40 (2.48, 35.63)
MDR-TB	Global	10-14 years	11430 (4215, 26206)	674 (200, 1534)	5.90 (1.50, 23.17)
MDR-TB	High SDI	<5 years	29 (17, 49)	1 (0, 1)	2.26 (0.81, 6.27)
MDR-TB	High SDI	5-9 years	24 (13, 41)	0 (0, 0)	0.84 (0.28, 2.49)
MDR-TB	High SDI	10-14 years	36 (18, 66)	0 (0, 1)	0.79 (0.25, 2.50)
MDR-TB	High-middle SDI	<5 years	405 (230, 793)	21 (11, 34)	5.18 (2.22, 12.07)
MDR-TB	High-middle SDI	5-9 years	465 (258, 763)	5 (2, 9)	1.01 (0.43, 2.38)
MDR-TB	High-middle SDI	10-14 years	739 (370, 1264)	6 (2, 11)	0.75 (0.28, 2.02)

Disease	Location	Age group	Incident cases	Deaths	MIR (%)
MDR-TB	Middle SDI	<5 years	2188 (1195, 3504)	277 (114, 554)	12.68 (4.88, 32.94)
MDR-TB	Middle SDI	5-9 years	1644 (806, 3079)	71 (28, 139)	4.30 (1.51, 12.27)
MDR-TB	Middle SDI	10-14 years	2669 (1229, 4986)	89 (33, 176)	3.32 (1.11, 9.93)
MDR-TB	Low-middle SDI	<5 years	4721 (2526, 7959)	1396 (496, 2862)	29.56 (10.37, 84.25)
MDR-TB	Low-middle SDI	5-9 years	2804 (1246, 5430)	301 (100, 604)	10.72 (3.35, 34.28)
MDR-TB	Low-middle SDI	10-14 years	4693 (2024, 9891)	336 (110, 689)	7.16 (2.13, 24.06)
MDR-TB	Low SDI	<5 years	6641 (4107, 10471)	2849 (1116, 6063)	42.90 (16.31, 112.83)
MDR-TB	Low SDI	5-9 years	2154 (1095, 3774)	291 (113, 599)	13.52 (4.79, 38.13)
MDR-TB	Low SDI	10-14 years	3292 (1642, 6218)	243 (96, 482)	7.38 (2.60, 20.99)
MDR-TB	Andean Latin America	<5 years	63 (29, 130)	11 (4, 25)	17.46 (5.34, 57.06)
MDR-TB	Andean Latin America	5-9 years	56 (23, 118)	4 (1, 8)	7.14 (1.90, 26.81)
MDR-TB	Andean Latin America	10-14 years	96 (40, 211)	4 (1, 9)	4.17 (1.05, 16.53)
MDR-TB	Australasia	<5 years	1 (0, 2)	0 (0, 0)	0.00 (0.00, 0.00)
MDR-TB	Australasia	5-9 years	1 (0, 1)	0 (0, 0)	0.00 (0.00, 0.00)
MDR-TB	Australasia	10-14 years	1 (0, 2)	0 (0, 0)	0.00 (0.00, 0.00)
MDR-TB	Caribbean	<5 years	4 (1, 12)	2 (0, 7)	50.00 (0.00, 139362531.41)
MDR-TB	Caribbean	5-9 years	2 (1, 7)	0 (0, 1)	0.00 (0.00, 0.00)
MDR-TB	Caribbean	10-14 years	3 (1, 10)	0 (0, 1)	0.00 (0.00, 0.00)
MDR-TB	Central Asia	<5 years	294 (180, 443)	73 (37, 120)	24.83 (11.84, 52.09)
MDR-TB	Central Asia	5-9 years	250 (138, 423)	7 (4, 11)	2.80 (1.32, 5.96)
MDR-TB	Central Asia	10-14 years	322 (173, 566)	6 (3, 9)	1.86 (0.83, 4.18)
MDR-TB	Central Europe	<5 years	4 (2, 8)	1 (0, 1)	25.00 (0.00, 25438227.71)
MDR-TB	Central Europe	5-9 years	4 (2, 9)	0 (0, 0)	0.00 (0.00, 0.00)
MDR-TB	Central Europe	10-14 years	6 (2, 13)	0 (0, 0)	0.00 (0.00, 0.00)
MDR-TB	Central Latin America	<5 years	24 (11, 43)	3 (1, 7)	12.50 (3.81, 41.01)
MDR-TB	Central Latin America	5-9 years	18 (7, 37)	1 (0, 3)	5.56 (0.00, 9857257.33)
MDR-TB	Central Latin America	10-14 years	33 (13, 71)	2 (1, 4)	6.06 (2.03, 18.13)
MDR-TB	Central Sub-Saharan Africa	<5 years	1128 (346, 2771)	352 (84, 1100)	31.21 (5.97, 163.17)
MDR-TB	Central Sub-Saharan Africa	5-9 years	321 (93, 899)	52 (13, 164)	16.20 (2.96, 88.76)
MDR-TB	Central Sub-Saharan Africa	10-14 years	464 (142, 1276)	41 (10, 134)	8.84 (1.61, 48.36)
MDR-TB	East Asia	<5 years	474 (120, 1370)	21 (5, 56)	4.43 (0.80, 24.62)
MDR-TB	East Asia	5-9 years	246 (56, 729)	4 (1, 12)	1.63 (0.27, 9.70)
MDR-TB	East Asia	10-14 years	427 (101, 1259)	6 (1, 15)	1.41 (0.22, 8.94)
MDR-TB	Eastern Europe	<5 years	171 (97, 280)	8 (4, 11)	4.68 (2.25, 9.73)

Disease	Location	Age group	Incident cases	Deaths	MIR (%)
MDR-TB	Eastern Europe	5-9 years	313 (155, 560)	1 (1, 2)	0.32 (0.15, 0.66)
MDR-TB	Eastern Europe	10-14 years	498 (236, 884)	1 (1, 2)	0.20 (0.10, 0.42)
MDR-TB	Eastern Sub-Saharan Africa	<5 years	3025 (1764, 5069)	1255 (422, 2764)	41.49 (14.12, 121.90)
MDR-TB	Eastern Sub-Saharan Africa	5-9 years	897 (423, 1619)	91 (34, 197)	10.14 (3.36, 30.64)
MDR-TB	Eastern Sub-Saharan Africa	10-14 years	1367 (639, 2512)	76 (28, 160)	5.56 (1.84, 16.84)
MDR-TB	High-income Asia Pacific	<5 years	1 (0, 2)	0 (0, 0)	0.00 (0.00, 0.00)
MDR-TB	High-income Asia Pacific	5-9 years	1 (0, 2)	0 (0, 0)	0.00 (0.00, 0.00)
MDR-TB	High-income Asia Pacific	10-14 years	1 (0, 3)	0 (0, 0)	0.00 (0.00, 0.00)
MDR-TB	High-income North America	<5 years	3 (1, 8)	0 (0, 0)	0.00 (0.00, 0.00)
MDR-TB	High-income North America	5-9 years	2 (1, 5)	0 (0, 0)	0.00 (0.00, 0.00)
MDR-TB	High-income North America	10-14 years	3 (1, 6)	0 (0, 0)	0.00 (0.00, 0.00)
MDR-TB	North Africa and Middle East	<5 years	251 (106, 621)	91 (20, 282)	36.25 (7.38, 178.00)
MDR-TB	North Africa and Middle East	5-9 years	167 (72, 387)	16 (4, 48)	9.58 (2.14, 42.95)
MDR-TB	North Africa and Middle East	10-14 years	263 (106, 630)	17 (4, 50)	6.46 (1.38, 30.32)
MDR-TB	Oceania	<5 years	32 (9, 75)	14 (3, 40)	43.75 (8.21, 233.27)
MDR-TB	Oceania	5-9 years	19 (5, 46)	3 (1, 9)	15.79 (3.31, 75.25)
MDR-TB	Oceania	10-14 years	26 (7, 65)	2 (1, 6)	7.69 (1.84, 32.14)
MDR-TB	South Asia	<5 years	4382 (1504, 8780)	1249 (371, 2846)	28.50 (7.41, 109.69)
MDR-TB	South Asia	5-9 years	3260 (1136, 7620)	320 (97, 688)	9.82 (2.51, 38.46)
MDR-TB	South Asia	10-14 years	5584 (1839, 13484)	389 (107, 849)	6.97 (1.66, 29.31)
MDR-TB	Southeast Asia	<5 years	658 (301, 1262)	101 (34, 222)	15.35 (4.71, 49.98)
MDR-TB	Southeast Asia	5-9 years	520 (210, 1081)	27 (9, 61)	5.19 (1.47, 18.30)
MDR-TB	Southeast Asia	10-14 years	744 (317, 1535)	29 (10, 64)	3.90 (1.15, 13.18)
MDR-TB	Southern Latin America	<5 years	2 (0, 7)	0 (0, 1)	0.00 (0.00, 0.03)
MDR-TB	Southern Latin America	5-9 years	3 (1, 10)	0 (0, 0)	0.00 (0.00, 0.00)
MDR-TB	Southern Latin America	10-14 years	4 (1, 14)	0 (0, 0)	0.00 (0.00, 0.00)
MDR-TB	Southern Sub-Saharan Africa	<5 years	654 (306, 1435)	161 (54, 376)	24.62 (7.12, 85.10)
MDR-TB	Southern Sub-Saharan Africa	5-9 years	337 (135, 751)	35 (12, 81)	10.39 (2.88, 37.49)
MDR-TB	Southern Sub-Saharan Africa	10-14 years	521 (193, 1337)	29 (10, 66)	5.57 (1.44, 21.51)
MDR-TB	Tropical Latin America	<5 years	34 (8, 100)	5 (1, 14)	14.71 (2.37, 91.35)
MDR-TB	Tropical Latin America	5-9 years	22 (5, 68)	1 (0, 2)	4.55 (0.00, 6825759.68)
MDR-TB	Tropical Latin America	10-14 years	42 (9, 127)	1 (0, 3)	2.38 (0.00, 4382618.48)
MDR-TB	Western Europe	<5 years	11 (6, 19)	0 (0, 0)	0.00 (0.00, 0.00)
MDR-TB	Western Europe	5-9 years	11 (6, 20)	0 (0, 0)	0.00 (0.00, 0.00)

Disease	Location	Age group	Incident cases	Deaths	MIR (%)
MDR-TB	Western Europe	10-14 years	15 (7, 28)	0 (0, 0)	0.00 (0.00, 0.00)
MDR-TB	Western Sub-Saharan Africa	<5 years	2772 (1222, 6256)	1197 (342, 3305)	43.18 (10.67, 174.68)
MDR-TB	Western Sub-Saharan Africa	5-9 years	645 (257, 1446)	105 (33, 267)	16.28 (4.19, 63.18)
MDR-TB	Western Sub-Saharan Africa	10-14 years	1010 (388, 2173)	71 (23, 162)	7.03 (1.91, 25.84)
All-form TB	Global	<5 years	685438 (531580, 866750)	110335 (78054, 150272)	16.10 (10.70, 24.22)
All-form TB	Global	5-9 years	323576 (206010, 487652)	15776 (12490, 19706)	4.88 (2.99, 7.94)
All-form TB	Global	10-14 years	509587 (313947, 752618)	15208 (12733, 17945)	2.98 (1.87, 4.77)
All-form TB	High SDI	<5 years	1189 (919, 1548)	13 (11, 16)	1.11 (0.82, 1.51)
All-form TB	High SDI	5-9 years	1001 (639, 1518)	4 (4, 5)	0.42 (0.26, 0.66)
All-form TB	High SDI	10-14 years	1535 (895, 2402)	6 (5, 7)	0.38 (0.23, 0.65)
All-form TB	High-middle SDI	<5 years	6436 (5048, 8164)	240 (193, 296)	3.72 (2.70, 5.14)
All-form TB	High-middle SDI	5-9 years	4832 (3182, 7176)	55 (47, 63)	1.13 (0.73, 1.74)
All-form TB	High-middle SDI	10-14 years	7811 (4867, 11970)	74 (64, 85)	0.95 (0.59, 1.52)
All-form TB	Middle SDI	<5 years	57617 (44810, 72352)	3751 (3017, 4664)	6.51 (4.71, 9.00)
All-form TB	Middle SDI	5-9 years	40533 (26375, 60493)	960 (833, 1111)	2.37 (1.53, 3.68)
All-form TB	Middle SDI	10-14 years	63738 (40618, 91090)	1154 (1036, 1311)	1.81 (1.19, 2.76)
All-form TB	Low-middle SDI	<5 years	99177 (76481, 125633)	14557 (11114, 18277)	14.68 (10.33, 20.86)
All-form TB	Low-middle SDI	5-9 years	57350 (37035, 85183)	3019 (2456, 3554)	5.26 (3.34, 8.30)
All-form TB	Low-middle SDI	10-14 years	94251 (58068, 138704)	3296 (2797, 3742)	3.50 (2.21, 5.54)
All-form TB	Low SDI	<5 years	178106 (139196, 225732)	36573 (24986, 49284)	20.53 (13.53, 31.15)
All-form TB	Low SDI	5-9 years	57963 (36777, 88259)	3843 (2929, 4885)	6.63 (3.99, 11.01)
All-form TB	Low SDI	10-14 years	87293 (52579, 131165)	3068 (2519, 3703)	3.51 (2.14, 5.77)
All-form TB	Andean Latin America	<5 years	954 (735, 1198)	92 (68, 122)	9.64 (6.59, 14.11)
All-form TB	Andean Latin America	5-9 years	813 (526, 1209)	29 (22, 37)	3.57 (2.18, 5.83)
All-form TB	Andean Latin America	10-14 years	1398 (885, 2086)	32 (24, 41)	2.29 (1.38, 3.79)
All-form TB	Australasia	<5 years	38 (28, 51)	0 (0, 0)	0.00 (0.00, 0.00)
All-form TB	Australasia	5-9 years	23 (14, 35)	0 (0, 0)	0.00 (0.00, 0.00)
All-form TB	Australasia	10-14 years	29 (17, 47)	0 (0, 0)	0.00 (0.00, 0.00)
All-form TB	Caribbean	<5 years	1017 (794, 1297)	207 (137, 328)	20.35 (12.34, 33.58)
All-form TB	Caribbean	5-9 years	548 (348, 822)	38 (23, 74)	6.93 (3.36, 14.32)
All-form TB	Caribbean	10-14 years	837 (511, 1257)	32 (20, 63)	3.82 (1.84, 7.93)
All-form TB	Central Asia	<5 years	1431 (1126, 1819)	294 (226, 378)	20.55 (14.45, 29.20)
All-form TB	Central Asia	5-9 years	1158 (790, 1661)	28 (24, 32)	2.42 (1.62, 3.60)
All-form TB	Central Asia	10-14 years	1497 (932, 2153)	21 (19, 24)	1.40 (0.91, 2.17)

Disease	Location	Age group	Incident cases	Deaths	MIR (%)
All-form TB	Central Europe	<5 years	193 (146, 251)	11 (9, 14)	5.70 (4.02, 8.08)
All-form TB	Central Europe	5-9 years	196 (127, 290)	1 (0, 1)	0.51 (0.00, 513360.31)
All-form TB	Central Europe	10-14 years	289 (178, 426)	1 (1, 1)	0.35 (0.22, 0.54)
All-form TB	Central Latin America	<5 years	853 (685, 1085)	59 (44, 79)	6.92 (4.77, 10.04)
All-form TB	Central Latin America	5-9 years	631 (406, 938)	21 (18, 25)	3.33 (2.12, 5.22)
All-form TB	Central Latin America	10-14 years	1183 (719, 1818)	30 (26, 34)	2.54 (1.56, 4.11)
All-form TB	Central Sub-Saharan Africa	<5 years	49725 (38657, 62936)	6993 (4203, 11233)	14.06 (8.13, 24.34)
All-form TB	Central Sub-Saharan Africa	5-9 years	14476 (9132, 21724)	1032 (700, 1494)	7.13 (4.01, 12.68)
All-form TB	Central Sub-Saharan Africa	10-14 years	21004 (12832, 31753)	825 (564, 1206)	3.93 (2.17, 7.09)
All-form TB	East Asia	<5 years	11329 (9054, 14159)	270 (216, 339)	2.38 (1.74, 3.27)
All-form TB	East Asia	5-9 years	5968 (3952, 8824)	56 (46, 71)	0.94 (0.59, 1.48)
All-form TB	East Asia	10-14 years	10278 (6387, 15482)	73 (61, 90)	0.71 (0.44, 1.15)
All-form TB	Eastern Europe	<5 years	601 (436, 815)	24 (21, 26)	3.99 (2.87, 5.56)
All-form TB	Eastern Europe	5-9 years	1102 (667, 1662)	4 (4, 5)	0.36 (0.23, 0.58)
All-form TB	Eastern Europe	10-14 years	1727 (1009, 2644)	4 (3, 4)	0.23 (0.14, 0.38)
All-form TB	Eastern Sub-Saharan Africa	<5 years	74189 (56741, 94223)	13987 (9878, 19029)	18.85 (12.46, 28.54)
All-form TB	Eastern Sub-Saharan Africa	5-9 years	24264 (14470, 37918)	1121 (847, 1468)	4.62 (2.65, 8.04)
All-form TB	Eastern Sub-Saharan Africa	10-14 years	38503 (21574, 59617)	936 (739, 1171)	2.43 (1.39, 4.25)
All-form TB	High-income Asia Pacific	<5 years	54 (40, 72)	2 (2, 2)	3.70 (2.76, 4.97)
All-form TB	High-income Asia Pacific	5-9 years	52 (32, 79)	1 (1, 1)	1.92 (1.22, 3.02)
All-form TB	High-income Asia Pacific	10-14 years	85 (48, 134)	1 (1, 1)	1.18 (0.70, 1.97)
All-form TB	High-income North America	<5 years	217 (161, 288)	3 (2, 3)	1.38 (0.97, 1.97)
All-form TB	High-income North America	5-9 years	129 (76, 205)	1 (1, 1)	0.78 (0.47, 1.27)
All-form TB	High-income North America	10-14 years	172 (97, 278)	1 (1, 1)	0.58 (0.34, 0.98)
All-form TB	North Africa and Middle East	<5 years	7335 (5741, 9404)	1252 (920, 1668)	17.07 (11.60, 25.12)
All-form TB	North Africa and Middle East	5-9 years	5698 (3617, 8519)	232 (172, 344)	4.07 (2.35, 7.06)
All-form TB	North Africa and Middle East	10-14 years	8993 (5559, 13475)	236 (179, 339)	2.62 (1.52, 4.53)
All-form TB	Oceania	<5 years	814 (609, 1044)	206 (108, 314)	25.31 (13.92, 46.01)
All-form TB	Oceania	5-9 years	581 (361, 865)	47 (32, 70)	8.09 (4.50, 14.54)
All-form TB	Oceania	10-14 years	806 (500, 1147)	36 (26, 51)	4.47 (2.62, 7.62)
All-form TB	South Asia	<5 years	62011 (46601, 79568)	9936 (7809, 12478)	16.02 (11.23, 22.87)
All-form TB	South Asia	5-9 years	49619 (32489, 72371)	2585 (2062, 3090)	5.21 (3.33, 8.16)
All-form TB	South Asia	10-14 years	82483 (52134, 120808)	3114 (2630, 3574)	3.78 (2.41, 5.90)
All-form TB	Southeast Asia	<5 years	34258 (27224, 42896)	2861 (2224, 3574)	8.35 (6.01, 11.60)

Disease	Location	Age group	Incident cases	Deaths	MIR (%)
All-form TB	Southeast Asia	5-9 years	25236 (15795, 38210)	712 (599, 837)	2.82 (1.76, 4.52)
All-form TB	Southeast Asia	10-14 years	37467 (24179, 53372)	847 (724, 995)	2.26 (1.48, 3.46)
All-form TB	Southern Latin America	<5 years	162 (117, 216)	7 (6, 9)	4.32 (2.99, 6.24)
All-form TB	Southern Latin America	5-9 years	242 (155, 352)	3 (2, 3)	1.24 (0.78, 1.96)
All-form TB	Southern Latin America	10-14 years	337 (202, 504)	4 (4, 5)	1.19 (0.74, 1.90)
All-form TB	Southern Sub-Saharan Africa	<5 years	16582 (12644, 20950)	1837 (1431, 2377)	11.08 (7.74, 15.85)
All-form TB	Southern Sub-Saharan Africa	5-9 years	8913 (5523, 13571)	410 (312, 514)	4.60 (2.75, 7.69)
All-form TB	Southern Sub-Saharan Africa	10-14 years	14046 (7772, 20985)	354 (297, 427)	2.52 (1.49, 4.28)
All-form TB	Tropical Latin America	<5 years	1042 (797, 1342)	81 (63, 104)	7.77 (5.42, 11.16)
All-form TB	Tropical Latin America	5-9 years	683 (411, 1046)	11 (9, 14)	1.61 (0.96, 2.70)
All-form TB	Tropical Latin America	10-14 years	1292 (774, 1964)	15 (13, 17)	1.16 (0.72, 1.88)
All-form TB	Western Europe	<5 years	452 (325, 613)	2 (2, 2)	0.44 (0.32, 0.61)
All-form TB	Western Europe	5-9 years	519 (320, 786)	1 (1, 1)	0.19 (0.12, 0.30)
All-form TB	Western Europe	10-14 years	770 (442, 1251)	1 (1, 1)	0.13 (0.08, 0.22)
All-form TB	Western Sub-Saharan Africa	<5 years	79461 (62268, 99933)	17044 (10798, 26560)	21.45 (12.90, 35.66)
All-form TB	Western Sub-Saharan Africa	5-9 years	20937 (12987, 31980)	1556 (1081, 2182)	7.43 (4.20, 13.16)
All-form TB	Western Sub-Saharan Africa	10-14 years	31598 (19186, 46699)	1041 (793, 1341)	3.29 (1.97, 5.52)

Note: Data are presented as estimated absolute numbers with 95% uncertainty intervals (UIs) in parentheses. Abbreviations: TB: tuberculosis; MDR-TB: multidrug-resistant tuberculosis; MIR: mortality-to-incidence ratio; SDI: socio-demographic index. Note: MIR (%) is calculated as deaths/incident cases×100%.

Table S4. Incidence MDR-to-all-form ratio (MAR) globally, by SDI, and GBD region

Location	1990	1995	2000	2005	2010	2015	2021
Global	0.57 (0.00–1.15)	2.31 (1.01–3.62)	3.43 (1.50–5.35)	3.93 (1.91–5.95)	4.01 (1.72–6.29)	3.95 (1.51–6.38)	4.27 (1.51–7.03)
High SDI	1.02 (0.16–1.88)	3.16 (0.65–5.66)	3.34 (1.21–5.48)	3.16 (1.22–5.10)	2.47 (1.00–3.95)	2.27 (0.88–3.67)	2.40 (0.67–4.12)
High-middle SDI	1.94 (0.00–4.51)	7.69 (2.33–13.05)	8.76 (3.34–14.17)	9.25 (4.23–14.28)	9.62 (3.98–15.27)	9.06 (3.42–14.70)	8.27 (2.37–14.18)
Middle SDI	1.18 (0.00–2.62)	3.98 (1.46–6.51)	4.60 (1.73–7.48)	4.85 (2.02–7.68)	4.42 (1.72–7.12)	3.77 (1.25–6.29)	4.00 (1.12–6.88)
Low-middle SDI	0.12 (0.01–0.24)	1.20 (0.11–2.30)	2.88 (0.71–5.04)	3.85 (1.60–6.10)	4.12 (1.35–6.89)	4.30 (1.12–7.47)	4.87 (1.01–8.72)
Low SDI	0.23 (0.06–	1.41 (0.43–2.40)	2.57 (0.94–4.19)	3.00 (1.22–4.78)	3.28 (1.43–5.13)	3.44 (1.32–5.56)	3.74 (1.28–6.20)

Location	1990	1995	2000	2005	2010	2015	2021
	0.40)						
Andean Latin America	0.84 (0.00–1.89)	3.23 (0.39–6.07)	7.12 (1.45–12.79)	8.31 (3.13–13.49)	6.66 (2.69–10.63)	5.67 (1.86–9.47)	6.78 (0.47–13.09)
Australasia	0.52 (0.00–1.18)	0.86 (0.00–1.74)	0.85 (0.00–1.74)	1.29 (0.05–2.52)	1.88 (0.35–3.41)	2.74 (0.84–4.63)	2.72 (0.00–5.52)
Caribbean	0.35 (0.00–0.80)	0.91 (0.00–1.92)	0.93 (0.00–2.10)	0.57 (0.00–1.37)	0.31 (0.00–0.74)	0.42 (0.00–0.97)	0.42 (0.00–0.98)
Central Asia	0.16 (0.00–0.36)	3.23 (0.41–6.06)	8.56 (2.93–14.20)	18.76 (7.91–29.61)	25.60 (12.71–38.49)	24.01 (11.11–36.91)	21.19 (7.52–34.86)
Central Europe	0.67 (0.00–1.39)	2.17 (0.81–3.54)	1.97 (0.38–3.56)	2.85 (0.31–5.38)	2.86 (0.55–5.16)	2.75 (1.22–4.27)	2.08 (0.17–4.00)
Central Latin America	0.15 (0.00–0.29)	1.48 (0.30–2.67)	2.85 (0.57–5.13)	3.20 (1.19–5.22)	2.66 (1.02–4.29)	2.63 (0.70–4.57)	2.80 (0.34–5.26)
Central Sub-Saharan Africa	0.32 (0.00–0.83)	1.52 (0.00–3.69)	1.74 (0.00–4.06)	1.88 (0.00–4.31)	1.95 (0.00–4.34)	2.27 (0.00–5.36)	2.24 (0.00–4.91)
East Asia	3.12 (0.00–7.51)	10.47 (4.07–16.87)	10.00 (3.98–16.02)	8.16 (3.62–12.70)	5.35 (1.48–9.21)	3.81 (0.00–8.49)	4.16 (0.00–9.91)
Eastern Europe	1.15 (0.00–2.57)	10.27 (3.28–17.26)	17.40 (6.16–28.63)	24.21 (7.41–41.01)	29.26 (8.80–49.73)	30.41 (9.07–51.74)	28.63 (6.91–50.34)
Eastern Sub-Saharan Africa	0.11 (0.00–0.22)	1.11 (0.18–2.04)	2.45 (0.86–4.04)	2.35 (0.90–3.81)	2.68 (1.04–4.32)	3.34 (1.25–5.43)	3.86 (1.14–6.58)
High-income Asia Pacific	0.54 (0.00–1.30)	1.96 (0.29–3.64)	2.81 (1.20–4.41)	3.33 (1.21–5.45)	1.43 (0.00–2.92)	1.17 (0.00–2.70)	1.03 (0.00–2.57)
High-income North America	2.68 (0.48–4.88)	1.90 (0.64–3.16)	1.22 (0.39–2.04)	1.21 (0.50–1.93)	1.33 (0.57–2.10)	1.33 (0.54–2.12)	1.48 (0.00–3.16)
North Africa and Middle East	0.53 (0.06–1.00)	2.41 (0.61–4.21)	6.03 (2.24–9.82)	6.60 (2.40–10.81)	7.94 (3.30–12.59)	6.45 (1.48–11.42)	6.20 (0.00–12.78)
Oceania	0.05 (0.00–0.13)	0.33 (0.00–0.86)	0.66 (0.00–1.76)	0.88 (0.00–2.04)	1.30 (0.09–2.50)	3.89 (1.44–6.34)	3.47 (0.00–7.38)
South Asia	0.18 (0.00–0.44)	2.24 (0.00–5.53)	6.65 (0.21–13.09)	10.01 (3.68–16.34)	10.96 (2.37–19.54)	11.76 (0.91–22.60)	13.65 (0.00–27.53)
Southeast Asia	0.21 (0.00–0.46)	1.74 (0.00–3.91)	2.29 (0.00–4.68)	2.07 (0.24–3.90)	2.04 (0.67–3.41)	1.85 (0.72–2.97)	1.98 (0.27–3.69)
Southern Latin America	0.34 (0.00–	1.22 (0.00–2.54)	2.17 (0.45–3.88)	2.87 (1.04–4.70)	1.64 (0.02–3.25)	1.30 (0.00–3.25)	1.26 (0.00–3.23)

Location	1990	1995	2000	2005	2010	2015	2021
	0.85)						
Southern Sub-Saharan Africa	0.42 (0.00–1.00)	1.37 (0.11–2.63)	3.12 (0.00–6.41)	3.99 (0.00–8.08)	3.51 (0.55–6.47)	3.23 (0.57–5.88)	3.83 (0.00–7.74)
Tropical Latin America	0.06 (0.00–0.15)	0.67 (0.00–1.75)	2.44 (0.00–5.33)	2.43 (0.52–4.34)	2.31 (0.69–3.93)	2.76 (0.00–6.03)	3.24 (0.00–7.96)
Western Europe	0.74 (0.19–1.29)	1.37 (0.52–2.22)	1.80 (0.74–2.87)	2.28 (0.90–3.67)	2.04 (0.81–3.27)	2.05 (0.79–3.30)	2.16 (0.52–3.81)
Western Sub-Saharan Africa	0.31 (0.07–0.54)	1.66 (0.08–3.24)	2.51 (0.18–4.84)	3.33 (0.89–5.77)	3.55 (1.69–5.40)	3.18 (0.72–5.63)	3.35 (0.13–6.56)

Note: Data are presented as percentages with 95% uncertainty intervals (UIs) in parentheses. The MAR (%) was calculated as the ASIR of MDR-TB divided by the ASIR of all-form TB in children (aged 0–14 years), representing the proportion of MDR-TB within the aggregate all-form TB incidence. Abbreviations: SDI, Socio-demographic Index; ASIR, age-standardized incidence rate; MDR-TB, multidrug-resistant tuberculosis.

Table S5. Mortality MDR-to-all-form ratio (MAR) globally, by SDI, and GBD region

Location	1990	1995	2000	2005	2010	2015	2021
Global	0.77 (0.00–1.55)	3.56 (0.70–6.42)	5.87 (1.27–10.47)	6.86 (2.06–11.66)	7.34 (2.21–12.47)	7.64 (1.62–13.66)	8.32 (1.40–15.24)
High SDI	1.75 (0.12–3.37)	5.57 (0.97–10.17)	8.08 (2.03–14.13)	7.79 (2.40–13.18)	5.93 (1.59–10.26)	5.25 (1.07–9.42)	4.82 (0.18–9.45)
High-middle SDI	3.38 (0.00–8.08)	11.49 (3.29–19.69)	13.30 (4.19–22.41)	13.44 (4.96–21.93)	11.93 (5.18–18.67)	10.14 (4.11–16.16)	8.51 (3.05–13.97)
Middle SDI	2.37 (0.00–5.45)	7.58 (2.10–13.06)	8.56 (2.37–14.75)	8.76 (2.73–14.78)	8.17 (2.37–13.96)	7.56 (1.84–13.28)	7.44 (1.36–13.52)
Low-middle SDI	0.25 (0.00–0.53)	2.61 (0.00–5.28)	5.84 (0.49–11.19)	7.50 (1.87–13.13)	8.00 (2.12–13.88)	8.66 (1.86–15.46)	9.73 (1.20–18.26)
Low SDI	0.39 (0.00–0.78)	2.72 (0.06–5.37)	5.03 (0.67–9.40)	5.97 (1.32–10.63)	6.77 (1.68–11.85)	7.11 (1.03–13.20)	7.78 (0.66–14.90)
Andean Latin America	1.65 (0.00–3.99)	6.34 (0.00–13.00)	12.51 (1.45–23.56)	13.96 (2.88–25.05)	11.13 (1.98–20.28)	10.68 (0.37–20.99)	12.12 (0.26–23.99)
Australasia	1.39 (0.00–3.62)	2.17 (0.00–4.93)	2.23 (0.00–4.83)	3.13 (0.00–6.44)	3.99 (0.00–8.18)	5.76 (1.38–10.13)	5.75 (0.00–12.77)
Caribbean	0.64 (0.00–1.62)	1.72 (0.00–4.29)	1.69 (0.00–4.45)	1.10 (0.00–3.15)	0.62 (0.00–1.82)	0.82 (0.00–2.39)	0.82 (0.00–2.42)
Central Asia	0.27 (0.00–0.59)	4.52 (0.25–8.78)	12.24 (3.27–21.22)	23.00 (9.76–36.24)	29.74 (15.39–44.08)	28.72 (14.20–43.25)	25.15 (9.80–40.50)
Central Europe	1.52 (0.00–3.37)	4.63 (1.21–8.06)	4.26 (0.29–8.23)	5.84 (0.00–11.92)	5.53 (0.36–10.70)	5.90 (1.94–9.86)	4.72 (0.00–9.91)
Central Latin America	0.30 (0.00–0.61)	3.19 (0.17–6.22)	6.38 (1.32–11.44)	6.71 (1.82–11.61)	5.59 (1.24–9.93)	5.35 (0.73–9.97)	5.58 (0.19–10.97)

Location	1990	1995	2000	2005	2010	2015	2021
Central Sub-Saharan Africa	0.62 (0.00–1.81)	3.02 (0.00–7.58)	3.58 (0.00–8.70)	4.06 (0.00–9.79)	4.28 (0.00–10.61)	5.08 (0.00–13.17)	5.03 (0.00–12.72)
East Asia	5.75 (0.00–13.69)	17.48 (5.32–29.65)	17.05 (4.89–29.22)	14.33 (4.23–24.43)	10.14 (1.29–18.98)	7.46 (0.00–16.54)	7.96 (0.00–17.48)
Eastern Europe	2.57 (0.00–5.75)	19.10 (7.75–30.44)	27.72 (14.21–41.24)	32.01 (15.86–48.16)	33.38 (17.54–49.23)	33.78 (17.98–49.58)	32.00 (17.44–46.56)
Eastern Sub-Saharan Africa	0.20 (0.00–0.46)	2.27 (0.00–4.63)	5.16 (1.00–9.32)	5.18 (1.11–9.25)	6.42 (1.38–11.46)	7.72 (1.78–13.67)	8.86 (0.18–17.54)
High-income Asia Pacific	1.25 (0.00–3.44)	4.49 (0.00–9.33)	6.38 (1.15–11.61)	6.98 (1.82–12.14)	2.95 (0.00–6.47)	2.39 (0.00–5.92)	2.22 (0.00–5.47)
High-income North America	6.22 (0.72–11.72)	4.52 (1.31–7.74)	2.74 (0.90–4.57)	2.62 (0.82–4.41)	2.72 (0.46–4.97)	3.28 (1.36–5.20)	3.37 (0.00–7.04)
North Africa and Middle East	0.96 (0.00–1.99)	5.47 (0.00–11.15)	13.34 (0.00–26.94)	15.58 (0.18–30.99)	19.26 (3.85–34.66)	16.06 (0.00–33.88)	14.42 (0.00–35.44)
Oceania	0.10 (0.00–0.27)	0.66 (0.00–1.97)	1.30 (0.00–3.68)	1.68 (0.00–4.44)	2.51 (0.00–5.49)	7.64 (0.28–15.00)	6.87 (0.00–16.14)
South Asia	0.40 (0.00–1.00)	4.78 (0.00–11.48)	13.26 (0.00–27.55)	18.87 (4.31–33.43)	20.36 (3.85–36.87)	22.15 (2.38–41.91)	25.06 (0.07–50.05)
Southeast Asia	0.40 (0.00–0.98)	3.60 (0.00–8.16)	4.99 (0.00–10.43)	4.59 (0.32–8.87)	4.42 (0.96–7.87)	3.65 (0.69–6.61)	3.56 (0.15–6.96)
Southern Latin America	0.72 (0.00–1.72)	2.41 (0.00–5.00)	4.09 (0.73–7.45)	5.09 (1.37–8.81)	3.16 (0.13–6.19)	2.53 (0.00–5.53)	2.44 (0.00–6.09)
Southern Sub-Saharan Africa	0.91 (0.00–2.30)	2.86 (0.00–5.97)	5.35 (0.00–11.01)	7.40 (0.27–14.53)	7.67 (0.79–14.55)	7.73 (0.58–14.88)	8.67 (0.00–17.51)
Tropical Latin America	0.11 (0.00–0.35)	1.40 (0.00–3.98)	4.88 (0.00–11.24)	4.88 (0.24–9.52)	4.64 (0.59–8.69)	5.34 (0.00–11.48)	6.00 (0.00–14.13)
Western Europe	1.62 (0.40–2.84)	3.15 (0.94–5.36)	4.10 (1.34–6.87)	4.69 (1.52–7.87)	4.63 (1.65–7.61)	4.72 (1.66–7.79)	3.97 (0.98–6.97)
Western Sub-Saharan Africa	0.54 (0.00–1.13)	3.10 (0.00–6.97)	4.90 (0.00–10.51)	6.69 (0.32–13.07)	7.29 (1.21–13.37)	6.56 (0.00–13.32)	6.98 (0.00–16.00)

Note: Data are presented as percentages with 95% uncertainty intervals (UIs) in parentheses. The MAR (%) was calculated as the ASMR of MDR-TB divided by the ASMR of all-form TB in children (aged 0–14 years), representing the proportion of MDR-TB within the aggregate all-form TB mortality. Abbreviations: SDI, Socio-demographic Index; ASMR, age-standardized mortality rate; MDR-TB, multidrug-resistant tuberculosis.

Table S6. Joinpoint regression analysis of incidence MAR globally, by SDI, and GBD region

Location	Periods	APC (95% CI) (%)	P value (APC)	AAPC (95% CI) (%)	P value (AAPC)
Global	1990–1992	57.16 (45.72, 69.50)	<0.001	6.48 (5.71, 7.26)	<0.001
	1992–1995	16.98 (11.36, 22.89)	<0.001		

Location	Periods	APC (95% CI) (%)	P value (APC)	AAPC (95% CI) (%)	P value (AAPC)
	1995–1999	8.78 (6.35, 11.27)	<0.001		
	1999–2004	3.45 (2.01, 4.91)	<0.001		
	2004–2021	0.38 (0.22, 0.55)	<0.001		
High SDI	1990–1992	45.26 (39.29, 51.48)	<0.001	2.60 (2.11, 3.09)	<0.001
	1992–1995	14.16 (9.62, 18.90)	<0.001		
	1995–2004	-0.02 (-0.40, 0.35)	0.895		
	2004–2012	-4.48 (-4.87, -4.08)	<0.001		
	2012–2021	0.22 (-0.08, 0.53)	0.145		
High-middle SDI	1990–1992	57.91 (52.81, 63.17)	<0.001	4.61 (4.30, 4.91)	<0.001
	1992–1995	15.87 (13.49, 18.31)	<0.001		
	1995–2000	2.28 (1.71, 2.85)	<0.001		
	2000–2011	0.79 (0.67, 0.92)	<0.001		
	2011–2021	-1.54 (-1.68, -1.41)	<0.001		
Middle SDI	1990–1992	57.19 (52.54, 62.00)	<0.001	9.35 (9.03, 9.66)	<0.001
	1992–1995	10.36 (7.67, 13.11)	<0.001		
	1995–2004	2.05 (1.57, 2.53)	<0.001		
	2004–2014	-2.66 (-3.11, -2.21)	<0.001		
	2014–2021	0.61 (0.06, 1.17)	0.033		
Low-middle SDI	1990–1993	82.52 (74.45, 90.96)	<0.001	12.87 (12.35, 13.40)	<0.001
	1993–1997	21.03 (17.51, 24.64)	<0.001		
	1997–2001	14.16 (11.02, 17.39)	<0.001		
	2001–2014	2.50 (2.05, 2.95)	<0.001		
	2014–2021	1.95 (1.26, 2.65)	<0.001		
Low SDI	1990–1992	40.54 (36.19, 45.03)	<0.001	9.35 (9.04, 9.66)	<0.001
	1992–1995	16.53 (13.79, 19.33)	<0.001		
	1995–1999	13.91 (11.64, 16.23)	<0.001		
	1999–2004	3.73 (2.36, 5.12)	<0.001		
	2004–2021	1.34 (1.19, 1.49)	<0.001		
Andean Latin America	1990–1994	33.72 (26.39, 41.48)	<0.001	6.78 (5.66, 7.91)	<0.001
	1994–1998	19.10 (12.72, 25.84)	<0.001		
	1998–2004	7.02 (4.45, 9.66)	<0.001		
	2004–2015	-3.42 (-4.49, -2.33)	<0.001		
	2015–2021	3.03 (1.00, 5.10)	0.005		
Australasia	1990–2000	5.56 (4.50, 6.64)	<0.001	5.64 (5.26, 6.03)	<0.001

Location	Periods	APC (95% CI) (%)	P value (APC)	AAPC (95% CI) (%)	P value (AAPC)
	2000–2015	8.16 (7.47, 8.86)	<0.001		
	2015–2021	-0.32 (-1.41, 0.77)	0.546		
Caribbean	1990–1996	17.51 (14.28, 20.82)	<0.001	0.44 (-0.34, 1.23)	0.274
	1996–2008	-7.21 (-8.32, -6.08)	<0.001		
	2008–2021	0.71 (-0.08, 1.50)	0.076		
Central Asia	1990–1992	148.97 (135.26, 163.50)	<0.001	16.64 (15.54, 17.75)	<0.001
	1992–1995	45.47 (39.54, 51.64)	<0.001		
	1995–2000	21.05 (18.17, 24.00)	<0.001		
	2000–2005	16.89 (13.78, 20.09)	<0.001		
	2005–2021	0.81 (0.64, 0.98)	<0.001		
Central Europe	1990–1992	48.17 (27.88, 71.68)	<0.001	4.02 (2.52, 5.55)	<0.001
	1992–1995	13.77 (3.43, 25.14)	0.011		
	1995–2000	-2.88 (-6.05, 0.40)	0.081		
	2000–2006	8.09 (4.93, 11.35)	<0.001		
	2006–2021	-1.79 (-2.38, -1.21)	<0.001		
Central Latin America	1990–1992	114.73 (102.34, 127.88)	<0.001	9.94 (9.46, 10.42)	<0.001
	1992–1995	30.65 (25.17, 36.37)	<0.001		
	1995–1999	15.82 (12.43, 19.32)	<0.001		
	1999–2006	2.50 (1.47, 3.53)	<0.001		
	2006–2021	-0.73 (-0.95, -0.52)	<0.001		
Central Sub-Saharan Africa	1990–1992	67.24 (54.43, 81.12)	<0.001	6.30 (5.63, 6.97)	<0.001
	1992–1995	19.64 (13.06, 26.61)	<0.001		
	1995–2007	2.05 (1.11, 2.99)	<0.001		
	2007–2016	2.11 (1.11, 3.12)	<0.001		
	2016–2021	-0.47 (-1.55, 0.62)	0.38		
East Asia	1990–1992	50.04 (44.60, 55.69)	<0.001	0.76 (0.13, 1.39)	0.021
	1992–1995	14.15 (10.96, 17.43)	<0.001		
	1995–2003	-1.78 (-2.38, -1.18)	<0.001		
	2003–2013	-7.42 (-8.02, -6.82)	<0.001		
	2013–2021	0.04 (-0.75, 0.83)	0.923		
Eastern Europe	1990–1992	90.07 (75.54, 105.82)	<0.001	10.80 (9.48, 12.14)	<0.001
	1992–1996	30.13 (24.77, 35.73)	<0.001		
	1996–2000	9.94 (6.34, 13.65)	<0.001		
	2000–2007	6.27 (4.38, 8.20)	<0.001		

Location	Periods	APC (95% CI) (%)	P value (APC)	AAPC (95% CI) (%)	P value (AAPC)
	2007–2021	0.58 (0.33, 0.84)	<0.001		
Eastern Sub-Saharan Africa	1990–1992	101.32 (87.77, 115.86)	<0.001	12.14 (11.58, 12.70)	<0.001
	1992–1996	33.68 (27.87, 39.75)	<0.001		
	1996–2000	14.93 (10.95, 19.06)	<0.001		
	2000–2005	-0.90 (-2.57, 0.81)	0.287		
	2005–2021	3.16 (2.93, 3.38)	<0.001		
High-income Asia Pacific	1990–1995	29.23 (25.10, 33.49)	<0.001	2.12 (1.70, 2.54)	<0.001
	1995–2003	6.16 (5.22, 7.10)	<0.001		
	2003–2008	-6.56 (-8.33, -4.75)	<0.001		
	2008–2012	-13.79 (-17.15, -10.29)	<0.001		
	2012–2021	-2.04 (-2.78, -1.29)	<0.001		
High-income North America	1990–1998	-8.08 (-8.37, -7.79)	<0.001	-1.82 (-2.11, -1.54)	<0.001
	1998–2005	-1.61 (-2.48, -0.73)	0.001		
	2005–2015	1.01 (0.50, 1.52)	0.001		
	2015–2021	1.83 (1.02, 2.64)	<0.001		
North Africa and Middle East	1990–1992	42.15 (36.03, 48.55)	<0.001	8.35 (7.87, 8.83)	<0.001
	1992–1998	28.51 (26.35, 30.70)	<0.001		
	1998–2001	9.09 (4.81, 13.56)	<0.001		
	2001–2010	2.76 (2.23, 3.28)	<0.001		
	2010–2021	-2.25 (-2.48, -2.02)	<0.001		
Oceania	1990–1996	40.54 (36.93, 44.25)	<0.001	14.86 (14.28, 15.43)	<0.001
	1996–2004	9.77 (8.32, 11.23)	<0.001		
	2004–2014	16.03 (15.02, 17.06)	<0.001		
	2014–2021	-0.63 (-1.55, 0.30)	0.174		
South Asia	1990–1992	120.91 (105.77, 137.16)	<0.001	14.17 (12.82, 15.54)	<0.001
	1992–1996	34.33 (27.24, 41.81)	<0.001		
	1996–2001	20.89 (15.44, 26.59)	<0.001		
	2001–2010	4.41 (2.42, 6.44)	<0.001		
	2010–2021	2.05 (1.04, 3.07)	<0.001		
Southeast Asia	1990–1992	106.31 (96.79, 116.30)	<0.001	7.06 (6.33, 7.79)	<0.001
	1992–1995	24.11 (19.46, 28.94)	<0.001		
	1995–2000	5.56 (3.86, 7.30)	<0.001		
	2000–2015	-1.41 (-1.86, -0.96)	<0.001		
	2015–2021	1.15 (0.01, 2.31)	0.048		

Location	Periods	APC (95% CI) (%)	P value (APC)	AAPC (95% CI) (%)	P value (AAPC)
Southern Latin America	1990–1995	28.96 (24.36, 33.72)	<0.001	4.35 (3.73, 4.97)	<0.001
	1995–2004	9.47 (8.22, 10.74)	<0.001		
	2004–2011	-8.10 (-9.53, -6.64)	<0.001		
	2011–2021	-1.74 (-2.48, -0.98)	<0.001		
Southern Sub-Saharan Africa	1990–1994	64.63 (51.98, 78.33)	<0.001	7.42 (6.54, 8.30)	<0.001
	1994–1998	22.42 (14.07, 31.39)	<0.001		
	1998–2005	6.55 (4.27, 8.87)	<0.001		
	2005–2013	-2.62 (-4.18, -1.04)	0.002		
	2013–2021	2.11 (0.83, 3.41)	0.003		
Tropical Latin America	1990–1997	56.40 (49.88, 63.20)	<0.001	14.15 (13.06, 15.26)	<0.001
	1997–2002	14.41 (10.03, 18.96)	<0.001		
	2002–2009	-1.76 (-3.29, -0.20)	0.028		
	2009–2021	2.92 (2.19, 3.65)	<0.001		
Western Europe	1990–1993	23.51 (20.30, 26.80)	<0.001	3.52 (3.17, 3.86)	<0.001
	1993–2002	6.52 (5.83, 7.21)	<0.001		
	2002–2005	4.67 (2.07, 7.33)	0.001		
	2005–2013	-1.45 (-2.06, -0.84)	<0.001		
	2013–2021	0.70 (0.13, 1.28)	0.019		
Western Sub-Saharan Africa	1990–1992	39.81 (31.78, 48.33)	<0.001	8.45 (7.49, 9.43)	<0.001
	1992–1996	20.08 (15.11, 25.26)	<0.001		
	1996–2006	6.43 (5.36, 7.51)	<0.001		
	2006–2015	-0.80 (-2.02, 0.44)	0.19		
	2015–2021	0.82 (-0.66, 2.32)	0.26		

Notes: The MAR was calculated as the ASIR of MDR-TB divided by the ASIR of all-form TB in children (aged 0–14 years). Temporal trends were quantified using Joinpoint regression models. Abbreviations: ASIR, age-standardized incidence rate; APC, annual percentage change; AAPC, average annual percentage change; CI, confidence interval; SDI, Socio-demographic Index. *P* < 0.05 indicates statistical significance.

Table S7. Joinpoint regression analysis of mortality MAR globally, by SDI, and GBD region

Location	Periods	APC (95% CI) (%)	P value (APC)	AAPC (95% CI) (%)	P value (AAPC)
Global	1990–1992	49.73 (45.51, 54.08)	<0.001	7.90 (7.51, 8.29)	<0.001
	1992–1995	27.60 (24.77, 30.50)	<0.001		
	1995–1998	13.01 (10.35, 15.73)	<0.001		

Location	Periods	APC (95% CI) (%)	P value (APC)	AAPC (95% CI) (%)	P value (AAPC)
	1998–2003	4.90 (4.15, 5.65)	<0.001		
	2003–2021	1.19 (1.12, 1.26)	<0.001		
High SDI	1990–1992	41.25 (37.47, 45.14)	<0.001	3.31 (2.99, 3.62)	<0.001
	1992–1995	17.63 (14.71, 20.61)	<0.001		
	1995–2002	6.73 (6.32, 7.13)	<0.001		
	2002–2012	-5.07 (-5.25, -4.89)	<0.001		
	2012–2021	-1.16 (-1.39, -0.93)	<0.001		
High-middle SDI	1990–1992	47.57 (27.56, 70.72)	<0.001	2.72 (1.43, 4.02)	<0.001
	1992–1995	16.63 (6.44, 27.80)	0.002		
	1995–2003	1.84 (0.78, 2.92)	0.002		
	2003–2012	-2.03 (-2.75, -1.30)	<0.001		
	2012–2021	-4.04 (-4.63, -3.44)	<0.001		
Middle SDI	1990–1992	46.13 (37.63, 55.15)	<0.001	3.58 (3.02, 4.14)	<0.001
	1992–1995	14.41 (9.75, 19.27)	<0.001		
	1995–2002	2.05 (1.44, 2.66)	<0.001		
	2002–2021	-1.12 (-1.23, -1.02)	<0.001		
Low-middle SDI	1990–1993	71.51 (68.46, 74.62)	<0.001	12.47 (11.93, 13.02)	<0.001
	1993–1996	40.16 (35.54, 44.94)	<0.001		
	1996–1999	15.68 (12.00, 19.49)	<0.001		
	1999–2003	7.29 (5.75, 8.86)	<0.001		
	2003–2021	1.64 (1.56, 1.73)	<0.001		
Low SDI	1990–1993	57.07 (53.92, 60.29)	<0.001	10.12 (9.54, 10.70)	<0.001
	1993–1996	31.47 (26.49, 36.65)	<0.001		
	1996–1999	11.89 (7.92, 16.01)	<0.001		
	1999–2006	3.62 (3.05, 4.19)	<0.001		
	2006–2021	1.52 (1.38, 1.66)	<0.001		
Andean Latin America	1990–1992	45.46 (33.06, 59.02)	<0.001	6.59 (5.84, 7.34)	<0.001
	1992–1998	20.68 (18.83, 22.55)	<0.001		
	1998–2003	6.20 (4.26, 8.18)	<0.001		
	2003–2013	-3.57 (-4.05, -3.08)	<0.001		
	2013–2021	2.06 (1.35, 2.77)	<0.001		
Australasia	1990–1994	10.58 (3.82, 17.77)	0.003	4.61 (3.48, 5.77)	<0.001
	1994–2001	0.30 (-2.50, 3.18)	0.829		
	2001–2015	7.17 (6.45, 7.89)	<0.001		

Location	Periods	APC (95% CI) (%)	P value (APC)	AAPC (95% CI) (%)	P value (AAPC)
	2015–2021	0.10 (-2.10, 2.36)	0.923		
Caribbean	1990–1994	24.88 (21.04, 28.84)	<0.001	0.46 (-0.92, 1.86)	0.518
	1994–2002	-0.54 (-1.95, 0.88)	0.433		
	2002–2010	-11.06 (-12.59, -9.51)	<0.001		
	2010–2013	9.94 (-3.43, 25.15)	0.142		
	2013–2021	-0.62 (-1.98, 0.75)	0.351		
Central Asia	1990–1994	84.21 (79.29, 89.26)	<0.001	15.57 (14.72, 16.43)	<0.001
	1994–1997	36.06 (27.11, 45.64)	<0.001		
	1997–2005	14.39 (13.61, 15.17)	<0.001		
	2005–2011	4.71 (3.82, 5.61)	<0.001		
	2011–2021	-2.30 (-2.60, -2.00)	<0.001		
Central Europe	1990–1994	28.81 (21.09, 37.02)	<0.001	3.79 (2.21, 5.39)	<0.001
	1994–2000	-1.08 (-4.49, 2.46)	0.528		
	2000–2004	8.08 (-1.45, 18.52)	0.094		
	2004–2021	-0.62 (-1.27, 0.04)	0.065		
Central Latin America	1990–1994	67.46 (64.78, 70.19)	<0.001	9.71 (9.13, 10.29)	<0.001
	1994–1997	27.81 (21.96, 33.94)	<0.001		
	1997–2002	7.01 (5.61, 8.42)	<0.001		
	2002–2012	-3.08 (-3.42, -2.75)	<0.001		
	2012–2021	0.55 (0.14, 0.96)	0.012		
Central Sub-Saharan Africa	1990–1993	50.32 (44.71, 56.16)	<0.001	6.82 (5.83, 7.82)	<0.001
	1993–1996	15.62 (8.44, 23.27)	<0.001		
	1996–2012	1.69 (1.42, 1.96)	<0.001		
	2012–2015	4.24 (-2.46, 11.40)	0.205		
	2015–2021	-0.10 (-1.18, 0.98)	0.845		
East Asia	1990–1992	48.74 (39.78, 58.27)	<0.001	0.95 (0.45, 1.45)	<0.001
	1992–1996	9.85 (7.77, 11.97)	<0.001		
	1996–2005	-2.66 (-3.02, -2.30)	<0.001		
	2005–2014	-7.28 (-7.71, -6.84)	<0.001		
	2014–2021	0.66 (-0.14, 1.46)	0.101		
Eastern Europe	1990–1993	64.57 (60.21, 69.05)	<0.001	8.25 (7.82, 8.68)	<0.001
	1993–1996	24.06 (20.18, 28.06)	<0.001		
	1996–2004	3.98 (3.63, 4.34)	<0.001		
	2004–2013	0.82 (0.54, 1.10)	<0.001		

Location	Periods	APC (95% CI) (%)	P value (APC)	AAPC (95% CI) (%)	P value (AAPC)
	2013–2021	-0.87 (-1.13, -0.62)	<0.001		
Eastern Sub-Saharan Africa	1990–1994	69.92 (67.59, 72.28)	<0.001	13.01 (12.64, 13.39)	<0.001
	1994–1998	30.89 (28.67, 33.15)	<0.001		
	1998–2007	0.75 (0.45, 1.06)	<0.001		
	2007–2011	6.60 (5.16, 8.06)	<0.001		
	2011–2021	2.75 (2.52, 2.99)	<0.001		
High-income Asia Pacific	1990–1993	39.30 (31.11, 48.00)	<0.001	1.48 (0.63, 2.34)	<0.001
	1993–1997	11.16 (6.85, 15.64)	<0.001		
	1997–2004	5.87 (4.78, 6.97)	<0.001		
	2004–2010	-16.02 (-17.30, -14.71)	<0.001		
	2010–2021	-2.81 (-3.50, -2.12)	<0.001		
High-income North America	1990–1993	-1.51 (-6.72, 3.99)	0.564	-2.21 (-3.12, -1.30)	<0.001
	1993–1998	-14.44 (-17.03, -11.76)	<0.001		
	1998–2003	-2.98 (-6.07, 0.21)	0.065		
	2003–2015	2.46 (1.83, 3.09)	<0.001		
	2015–2021	-0.14 (-2.02, 1.78)	0.878		
North Africa and Middle East	1990–1996	42.50 (40.00, 45.05)	<0.001	9.29 (8.20, 10.40)	<0.001
	1996–1999	19.74 (8.74, 31.85)	<0.001		
	1999–2009	3.83 (2.93, 4.74)	<0.001		
	2009–2021	-2.36 (-3.00, -1.71)	<0.001		
Oceania	1990–1997	42.58 (39.58, 45.66)	<0.001	14.62 (13.11, 16.16)	<0.001
	1997–2008	3.38 (2.24, 4.53)	<0.001		
	2008–2011	21.54 (9.88, 34.43)	<0.001		
	2011–2014	36.28 (25.63, 47.84)	<0.001		
	2014–2021	-1.87 (-3.06, -0.66)	0.005		
South Asia	1990–1995	66.14 (64.47, 67.82)	<0.001	14.17 (13.65, 14.70)	<0.001
	1995–1999	23.50 (21.18, 25.86)	<0.001		
	1999–2002	10.85 (7.42, 14.39)	<0.001		
	2002–2005	4.92 (2.24, 7.67)	0.001		
	2005–2021	1.72 (1.62, 1.82)	<0.001		
Southeast Asia	1990–1994	62.19 (57.69, 66.83)	<0.001	7.06 (6.04, 8.08)	<0.001
	1994–1997	18.82 (9.57, 28.84)	<0.001		
	1997–2012	-0.89 (-1.18, -0.61)	<0.001		
	2012–2015	-5.22 (-9.94, -0.26)	0.04		

Location	Periods	APC (95% CI) (%)	P value (APC)	AAPC (95% CI) (%)	P value (AAPC)
	2015–2021	-0.69 (-1.66, 0.28)	0.152		
Southern Latin America	1990–1992	48.67 (33.32, 65.78)	<0.001	3.93 (3.06, 4.81)	<0.001
	1992–1999	12.80 (11.23, 14.40)	<0.001		
	1999–2005	5.62 (4.17, 7.09)	<0.001		
	2005–2012	-9.05 (-10.11, -7.97)	<0.001		
	2012–2021	-1.15 (-2.08, -0.22)	0.018		
Southern Sub-Saharan Africa	1990–1992	42.99 (31.00, 56.07)	<0.001	7.47 (6.63, 8.31)	<0.001
	1992–2002	13.58 (12.87, 14.29)	<0.001		
	2002–2009	2.51 (1.51, 3.53)	<0.001		
	2009–2012	-3.66 (-8.75, 1.72)	0.166		
	2012–2021	2.04 (1.50, 2.58)	<0.001		
Tropical Latin America	1990–1995	67.88 (65.16, 70.64)	<0.001	13.88 (12.99, 14.78)	<0.001
	1995–1998	35.55 (27.10, 44.56)	<0.001		
	1998–2001	14.34 (8.90, 20.06)	<0.001		
	2001–2009	-2.11 (-2.59, -1.62)	<0.001		
	2009–2021	2.49 (2.24, 2.74)	<0.001		
Western Europe	1990–1992	26.45 (11.40, 43.53)	0.001	2.77 (1.53, 4.02)	<0.001
	1992–2003	5.90 (4.95, 6.86)	<0.001		
	2003–2013	-1.15 (-2.17, -0.11)	0.032		
	2013–2017	2.93 (-2.94, 9.15)	0.315		
	2017–2021	-6.12 (-9.56, -2.56)	0.002		
Western Sub-Saharan Africa	1990–1992	58.27 (44.14, 73.78)	<0.001	8.45 (7.27, 9.64)	<0.001
	1992–1995	33.70 (21.40, 47.25)	<0.001		
	1995–2005	8.04 (7.07, 9.02)	<0.001		
	2005–2021	-0.30 (-0.64, 0.03)	0.076		

Notes: The MAR was calculated as the ASMR of MDR-TB divided by the ASMR of all-form TB in children (aged 0–14 years). Temporal trends were quantified using Joinpoint regression models. Abbreviations: ASMR, age-standardized mortality rate; APC, annual percentage change; AAPC, average annual percentage change; CI, confidence interval; SDI, Socio-demographic Index. $P < 0.05$ indicates statistical significance.

Table S8. Complementary interaction test of long-term temporal slope differences between MDR-TB and all-form TB

Location	Measure	Interaction Coefficient (β) (95% CI)	Interaction P-value
Global	Incidence	0.038 (0.025, 0.051)	<0.001
High SDI	Incidence	-0.002 (-0.011, 0.008)	0.737
High-middle SDI	Incidence	0.021 (0.009, 0.032)	<0.001
Middle SDI	Incidence	0.012 (0.002, 0.022)	0.024
Low-middle SDI	Incidence	0.077 (0.055, 0.099)	<0.001
Low SDI	Incidence	0.057 (0.040, 0.075)	<0.001
Andean Latin America	Incidence	0.038 (0.022, 0.054)	<0.001
Australasia	Incidence	0.056 (0.053, 0.060)	<0.001
Caribbean	Incidence	-0.027 (-0.039, -0.015)	<0.001
Central Asia	Incidence	0.114 (0.085, 0.144)	<0.001
Central Europe	Incidence	0.021 (0.012, 0.031)	<0.001
Central Latin America	Incidence	0.046 (0.024, 0.068)	<0.001
Central Sub-Saharan Africa	Incidence	0.033 (0.020, 0.046)	<0.001
East Asia	Incidence	-0.028 (-0.041, -0.015)	<0.001
Eastern Europe	Incidence	0.069 (0.051, 0.088)	<0.001
Eastern Sub-Saharan Africa	Incidence	0.071 (0.050, 0.092)	<0.001
High-income Asia Pacific	Incidence	-0.012 (-0.030, 0.006)	0.176
High-income North America	Incidence	-0.016 (-0.027, -0.004)	0.009
North Africa and Middle East	Incidence	0.057 (0.036, 0.078)	<0.001
Oceania	Incidence	0.119 (0.106, 0.132)	<0.001
South Asia	Incidence	0.095 (0.071, 0.120)	<0.001
Southeast Asia	Incidence	0.025 (0.009, 0.041)	0.002
Southern Latin America	Incidence	0.014 (-0.004, 0.032)	0.118
Southern Sub-Saharan Africa	Incidence	0.050 (0.031, 0.069)	<0.001
Tropical Latin America	Incidence	0.083 (0.055, 0.111)	<0.001
Western Europe	Incidence	0.024 (0.015, 0.033)	<0.001
Western Sub-Saharan Africa	Incidence	0.047 (0.030, 0.064)	<0.001
Global	Deaths	0.051 (0.035, 0.067)	<0.001
High SDI	Deaths	0.007 (-0.005, 0.019)	0.271
High-middle SDI	Deaths	0.004 (-0.011, 0.020)	0.563
Middle SDI	Deaths	0.012 (0.002, 0.023)	0.022
Low-middle SDI	Deaths	0.080 (0.057, 0.104)	<0.001

Location	Measure	Interaction Coefficient (β) (95% CI)	Interaction P-value
Low SDI	Deaths	0.065 (0.045, 0.085)	<0.001
Andean Latin America	Deaths	0.036 (0.021, 0.050)	<0.001
Australasia	Deaths	0.048 (0.041, 0.054)	<0.001
Caribbean	Deaths	-0.022 (-0.032, -0.013)	<0.001
Central Asia	Deaths	0.111 (0.076, 0.146)	<0.001
Central Europe	Deaths	0.021 (0.008, 0.034)	0.001
Central Latin America	Deaths	0.049 (0.027, 0.071)	<0.001
Central Sub-Saharan Africa	Deaths	0.040 (0.022, 0.057)	<0.001
East Asia	Deaths	-0.023 (-0.039, -0.008)	0.004
Eastern Europe	Deaths	0.047 (0.015, 0.078)	0.004
Eastern Sub-Saharan Africa	Deaths	0.082 (0.059, 0.104)	<0.001
High-income Asia Pacific	Deaths	-0.018 (-0.034, -0.002)	0.025
High-income North America	Deaths	-0.017 (-0.034, 0.000)	0.052
North Africa and Middle East	Deaths	0.066 (0.041, 0.090)	<0.001
Oceania	Deaths	0.123 (0.107, 0.139)	<0.001
South Asia	Deaths	0.096 (0.071, 0.122)	<0.001
Southeast Asia	Deaths	0.028 (0.007, 0.050)	0.01
Southern Latin America	Deaths	0.012 (-0.001, 0.026)	0.077
Southern Sub-Saharan Africa	Deaths	0.054 (0.037, 0.071)	<0.001
Tropical Latin America	Deaths	0.086 (0.059, 0.114)	<0.001
Western Europe	Deaths	0.020 (0.013, 0.027)	<0.001
Western Sub-Saharan Africa	Deaths	0.055 (0.036, 0.074)	<0.001

Note: The interaction coefficient (β) was derived from log-linear regression models (ln(Rate + 1×10^{-9}) ~ Year × Disease Type), representing the difference in the log-linear annual slope between multidrug-resistant tuberculosis (MDR-TB) and all-form TB. A positive coefficient indicates a more upward, or less downward, temporal trend for MDR-TB relative to all-form TB. Statistical significance of the interaction term ($P<0.05$) combined with a positive coefficient was used as an exploratory operational label for non-parallel temporal trends in which MDR-TB showed a more upward, or less downward, modeled trajectory than all-form TB. These results should be interpreted descriptively and not as definitive evidence of worsening MDR-TB epidemiology.. Abbreviations: CI, confidence interval; SDI, Socio-demographic Index.

Table S9. Temporal trends in female-to-male ratio (FMR) for pediatric all-form TB and MDR-TB globally and by SDI region

Location	Disease	Metric	1990	1995	2000	2005	2010	2015	2021
Global	All-form	ASIR	1.58 (0.83–	1.62 (0.90–	1.63 (0.87–	1.61 (0.85–	1.59 (0.81–	1.55 (0.81–	1.50 (0.78–

Location	Disease	Metric	1990	1995	2000	2005	2010	2015	2021
	TB		2.33)	2.35)	2.38)	2.37)	2.37)	2.29)	2.21)
	MDR-TB	ASIR	1.19 (0.00–2.85)	1.34 (0.45–2.23)	1.48 (0.52–2.43)	1.49 (0.66–2.32)	1.48 (0.53–2.42)	1.51 (0.41–2.61)	1.49 (0.34–2.65)
	All-form TB	ASMR	1.44 (0.93–1.95)	1.42 (0.95–1.89)	1.41 (0.97–1.85)	1.37 (0.98–1.77)	1.33 (0.94–1.72)	1.34 (0.96–1.73)	1.31 (0.77–1.84)
	MDR-TB	ASMR	1.20 (0.00–2.91)	1.29 (0.00–2.74)	1.37 (0.00–2.90)	1.35 (0.02–2.67)	1.32 (0.02–2.63)	1.38 (0.00–2.88)	1.36 (0.00–2.87)
High SDI	All-form TB	ASIR	1.50 (0.75–2.25)	1.47 (0.75–2.18)	1.50 (0.75–2.26)	1.39 (0.66–2.12)	1.26 (0.59–1.94)	1.22 (0.57–1.87)	1.23 (0.55–1.92)
	MDR-TB	ASIR	1.23 (0.00–2.58)	1.22 (0.00–2.48)	1.40 (0.31–2.48)	1.32 (0.40–2.23)	1.22 (0.41–2.04)	1.19 (0.36–2.03)	1.20 (0.18–2.22)
	All-form TB	ASMR	1.17 (0.76–1.58)	1.16 (0.82–1.50)	1.16 (0.87–1.44)	1.18 (0.94–1.42)	1.18 (0.94–1.43)	1.17 (0.88–1.45)	1.17 (0.84–1.50)
	MDR-TB	ASMR	0.96 (0.00–2.18)	0.96 (0.00–2.07)	1.05 (0.00–2.16)	1.08 (0.03–2.14)	1.07 (0.00–2.16)	1.11 (0.00–2.41)	1.09 (0.00–2.53)
High-middle SDI	All-form TB	ASIR	1.10 (0.55–1.65)	1.14 (0.62–1.67)	1.14 (0.61–1.67)	1.14 (0.60–1.69)	1.10 (0.56–1.65)	1.13 (0.57–1.68)	1.12 (0.55–1.69)
	MDR-TB	ASIR	0.98 (0.00–2.77)	1.06 (0.12–2.00)	1.08 (0.28–1.87)	1.14 (0.45–1.84)	1.12 (0.37–1.88)	1.17 (0.32–2.03)	1.18 (0.14–2.21)
	All-form TB	ASMR	1.00 (0.72–1.29)	0.95 (0.73–1.17)	0.91 (0.72–1.10)	0.90 (0.72–1.08)	0.89 (0.71–1.06)	0.86 (0.67–1.05)	0.82 (0.57–1.07)
	MDR-TB	ASMR	0.95 (0.00–2.80)	0.88 (0.00–1.77)	0.84 (0.03–1.65)	0.87 (0.10–1.63)	0.92 (0.18–1.65)	1.00 (0.17–1.83)	0.95 (0.11–1.79)
Middle SDI	All-form TB	ASIR	1.43 (0.75–2.11)	1.46 (0.81–2.11)	1.44 (0.78–2.10)	1.44 (0.74–2.13)	1.44 (0.72–2.16)	1.34 (0.69–1.98)	1.29 (0.67–1.90)
	MDR-TB	ASIR	1.20 (0.00–3.18)	1.28 (0.26–2.29)	1.35 (0.33–2.37)	1.40 (0.47–2.32)	1.39 (0.42–2.37)	1.35 (0.26–2.44)	1.34 (0.14–2.54)
	All-form TB	ASMR	1.20 (0.89–1.51)	1.18 (0.92–1.43)	1.17 (0.95–1.39)	1.14 (0.94–1.35)	1.10 (0.89–1.30)	1.06 (0.82–1.30)	1.05 (0.73–1.37)
	MDR-TB	ASMR	1.16 (0.00–3.27)	1.13 (0.00–2.30)	1.14 (0.00–2.32)	1.14 (0.01–2.27)	1.12 (0.00–2.23)	1.13 (0.00–2.31)	1.12 (0.00–2.38)
Low-middle SDI	All-form TB	ASIR	1.74 (0.84–2.64)	1.76 (0.92–2.60)	1.74 (0.88–2.60)	1.68 (0.88–2.49)	1.64 (0.83–2.46)	1.64 (0.82–2.45)	1.58 (0.79–2.38)
	MDR-TB	ASIR	1.49 (0.00–	1.59 (0.00–	1.65 (0.09–	1.58 (0.50–	1.54 (0.25–	1.58 (0.10–	1.56 (0.00–

Location	Disease	Metric	1990	1995	2000	2005	2010	2015	2021
			3.36)	3.49)	3.22)	2.67)	2.82)	3.05)	3.18)
	All-form TB	ASMR	1.47 (0.87–2.07)	1.44 (0.92–1.96)	1.41 (0.99–1.83)	1.35 (1.00–1.70)	1.30 (0.98–1.63)	1.33 (0.97–1.70)	1.30 (0.83–1.77)
	MDR-TB	ASMR	1.34 (0.00–3.50)	1.38 (0.00–3.41)	1.42 (0.00–3.27)	1.37 (0.00–2.83)	1.32 (0.00–2.70)	1.40 (0.00–2.94)	1.39 (0.00–3.12)
Low SDI	All-form TB	ASIR	1.64 (0.90–2.38)	1.67 (0.94–2.40)	1.68 (0.90–2.46)	1.67 (0.86–2.47)	1.63 (0.83–2.43)	1.59 (0.84–2.34)	1.54 (0.81–2.28)
	MDR-TB	ASIR	1.50 (0.07–2.92)	1.56 (0.15–2.97)	1.61 (0.32–2.89)	1.55 (0.47–2.63)	1.55 (0.56–2.53)	1.55 (0.43–2.68)	1.54 (0.29–2.78)
	All-form TB	ASMR	1.53 (0.87–2.19)	1.48 (0.85–2.11)	1.45 (0.83–2.07)	1.42 (0.81–2.02)	1.36 (0.78–1.95)	1.37 (0.84–1.90)	1.32 (0.66–1.98)
	MDR-TB	ASMR	1.43 (0.00–3.53)	1.46 (0.00–3.45)	1.45 (0.00–3.24)	1.38 (0.00–2.93)	1.35 (0.00–2.76)	1.38 (0.00–2.96)	1.34 (0.00–2.99)

Notes: The female-to-male ratio (FMR) was calculated as the rate in females divided by the corresponding rate in males. Values represent the point estimate followed by the 95% uncertainty interval in parentheses. Abbreviations: ASIR, age-standardized incidence rate; ASMR, age-standardized mortality rate; FMR, female-to-male ratio; MDR-TB, multidrug-resistant tuberculosis; SDI, Socio-demographic Index.

Table S10. Joinpoint regression analysis of FMR for pediatric all-form TB globally and by SDI region

Location	Metric	Periods	APC (95% CI) (%)	P value (APC)	AAPC (95% CI) (%)	P value (AAPC)
Global	ASIR	1990–1996	0.53 (0.48, 0.58)	<0.001	-0.18 (-0.21, -0.15)	<0.001
		1996–2003	-0.10 (-0.15, -0.05)	<0.001		
		2003–2013	-0.30 (-0.33, -0.27)	<0.001		
		2013–2019	-0.85 (-0.92, -0.78)	<0.001		
		2019–2021	0.03 (-0.29, 0.34)	0.863		
	ASMR	1990–2006	-0.35 (-0.40, -0.30)	<0.001	-0.32 (-0.41, -0.23)	<0.001
		2006–2011	-0.74 (-1.08, -0.39)	<0.001		
		2011–2016	0.51 (0.17, 0.86)	0.006		
		2016–2021	-0.65 (-0.95, -0.34)	<0.001		
High SDI	ASIR	1990–1994	-0.52 (-0.67, -0.37)	<0.001	-0.63 (-0.67, -0.59)	<0.001
		1994–2001	0.39 (0.30, 0.47)	<0.001		
		2001–2010	-1.97 (-2.03, -1.92)	<0.001		
		2010–2016	-0.61 (-0.73, -0.49)	<0.001		

Location	Metric	Periods	APC (95% CI) (%)	P value (APC)	AAPC (95% CI) (%)	P value (AAPC)
		2016–2021	0.31 (0.19, 0.43)	<0.001		
	ASMR	1990–2021	-0.02 (-0.06, 0.02)	0.280	-0.02 (-0.06, 0.02)	0.280
High-middle SDI	ASIR	1990–1995	0.81 (0.58, 1.05)	<0.001	0.02 (-0.08, 0.12)	0.711
		1995–2006	-0.05 (-0.13, 0.03)	0.198		
		2006–2010	-0.86 (-1.37, -0.34)	0.003		
		2010–2014	0.50 (-0.03, 1.04)	0.063		
		2014–2021	-0.21 (-0.35, -0.06)	0.007		
	ASMR	1990–1997	-1.29 (-1.62, -0.95)	<0.001	-0.70 (-0.81, -0.60)	<0.001
		1997–2012	-0.23 (-0.33, -0.14)	<0.001		
		2012–2021	-1.02 (-1.25, -0.80)	<0.001		
Middle SDI	ASIR	1990–1996	0.39 (0.32, 0.47)	<0.001	-0.35 (-0.39, -0.32)	<0.001
		1996–2001	-0.40 (-0.54, -0.25)	<0.001		
		2001–2011	0.03 (-0.01, 0.07)	0.185		
		2011–2017	-1.75 (-1.86, -1.65)	<0.001		
		2017–2021	-0.26 (-0.41, -0.12)	0.002		
	ASMR	1990–2008	-0.31 (-0.37, -0.26)	<0.001	-0.42 (-0.56, -0.28)	<0.001
		2008–2011	-1.58 (-2.99, -0.15)	0.032		
		2011–2021	-0.27 (-0.42, -0.12)	0.001		
Low-middle SDI	ASIR	1990–1993	0.35 (0.12, 0.58)	0.005	-0.31 (-0.35, -0.28)	<0.001
		1993–1998	0.04 (-0.10, 0.17)	0.595		
		1998–2009	-0.63 (-0.67, -0.60)	<0.001		
		2009–2015	-0.05 (-0.15, 0.05)	0.323		
		2015–2021	-0.61 (-0.69, -0.54)	<0.001		
	ASMR	1990–2012	-0.67 (-0.73, -0.62)	<0.001	-0.45 (-0.59, -0.31)	<0.001
		2012–2017	1.24 (0.61, 1.87)	<0.001		
		2017–2021	-1.30 (-2.05, -0.54)	0.002		
Low SDI	ASIR	1990–1998	0.27 (0.24, 0.31)	<0.001	-0.22 (-0.25, -0.18)	<0.001
		1998–2005	-0.11 (-0.17, -0.05)	0.002		
		2005–2015	-0.49 (-0.52, -0.46)	<0.001		
		2015–2019	-0.95 (-1.12, -0.77)	<0.001		
		2019–2021	0.26 (-0.10, 0.63)	0.151		
	ASMR	1990–1997	-0.75 (-0.87, -0.62)	<0.001	-0.49 (-0.56, -0.41)	<0.001
		1997–2003	-0.29 (-0.50, -0.08)	0.010		
		2003–2010	-0.74 (-0.90, -0.58)	<0.001		

Location	Metric	Periods	APC (95% CI) (%)	P value (APC)	AAPC (95% CI) (%)	P value (AAPC)
		2010–2016	0.07 (-0.12, 0.26)	0.461		
		2016–2021	-0.66 (-0.88, -0.45)	<0.001		

Notes: The female-to-male ratio (FMR) represents the rate in females divided by the corresponding rate in males. Temporal trends were quantified using Joinpoint regression models. Values represent the point estimate followed by the 95% CI in parentheses. Abbreviations: APC, annual percentage change; AAPC, average annual percentage change; ASIR, age-standardized incidence rate; ASMR, age-standardized mortality rate; CI, confidence interval; FMR, female-to-male ratio; TB, tuberculosis; SDI, Socio-demographic Index. $P < 0.05$ indicates statistical significance.

Table S11. Joinpoint regression analysis of FMR for pediatric MDR-TB globally and by SDI region

Location	Metric	Periods	APC (95% CI)(%)	P value(APC)	AAPC (95% CI)(%)	P value(AAPC)
Global	ASIR	1990–2000	1.95 (1.80, 2.10)	<0.001	0.65 (0.60, 0.70)	<0.001
		2000–2021	0.04 (0.00, 0.08)	0.032		
	ASMR	1990–1992	0.61 (-0.84, 2.09)	0.392	0.42 (0.29, 0.55)	<0.001
		1992–1998	1.99 (1.71, 2.26)	<0.001		
		1998–2012	-0.19 (-0.25, -0.13)	<0.001		
		2012–2016	1.41 (0.83, 1.99)	<0.001		
		2016–2021	-0.61 (-0.87, -0.34)	<0.001		
High SDI	ASIR	1990–1995	-0.23 (-0.60, 0.15)	0.220	-0.04 (-0.15, 0.07)	0.473
		1995–2000	3.43 (2.98, 3.89)	<0.001		
		2000–2009	-1.56 (-1.67, -1.44)	<0.001		
		2009–2017	-0.52 (-0.66, -0.37)	<0.001		
		2017–2021	0.32 (-0.10, 0.74)	0.125		
	ASMR	1990–1993	-0.98 (-2.87, 0.95)	0.299	0.39 (0.08, 0.70)	0.015
		1993–2002	1.83 (1.45, 2.21)	<0.001		
		2002–2009	-0.50 (-1.01, 0.02)	0.060		
		2009–2013	1.31 (-0.28, 2.92)	0.100		
		2013–2021	-0.38 (-0.79, 0.03)	0.067		
High-middle SDI	ASIR	1990–2001	0.52 (0.39, 0.65)	<0.001	0.44 (0.31, 0.56)	<0.001
		2001–2006	1.27 (0.89, 1.64)	<0.001		
		2006–2010	-0.85 (-1.43, -0.27)	0.006		
		2010–2015	1.01 (0.60, 1.42)	<0.001		
		2015–2021	-0.00 (-0.26, 0.25)	0.968		
	ASMR	1990–1998	-1.40 (-1.92, -0.88)	<0.001	0.06 (-0.34, 0.46)	0.786

Location	Metric	Periods	APC (95% CI)(%)	P value(APC)	AAPC (95% CI)(%)	P value(AAPC)
		1998–2006	0.55 (0.10, 1.01)	0.020		
		2006–2016	1.63 (1.36, 1.91)	<0.001		
		2016–2019	-3.73 (-6.79, -0.56)	0.024		
		2019–2021	1.92 (-1.37, 5.32)	0.240		
Middle SDI	ASIR	1990–2005	0.94 (0.89, 0.99)	<0.001	0.29 (0.21, 0.37)	<0.001
		2005–2012	-0.20 (-0.35, -0.05)	0.013		
		2012–2018	-0.79 (-1.02, -0.56)	<0.001		
		2018–2021	0.33 (-0.27, 0.93)	0.266		
	ASMR	1990–2003	-0.04 (-0.18, 0.11)	0.603	-0.03 (-0.29, 0.22)	0.799
		2003–2008	0.68 (0.01, 1.36)	0.046		
		2008–2011	-1.67 (-3.75, 0.44)	0.113		
		2011–2017	0.70 (0.21, 1.20)	0.008		
		2017–2021	-0.78 (-1.52, -0.03)	0.043		
Low-middle SDI	ASIR	1990–1992	2.54 (0.84, 4.27)	0.006	0.10 (-0.03, 0.24)	0.129
		1992–1999	0.66 (0.41, 0.90)	<0.001		
		1999–2010	-0.70 (-0.78, -0.63)	<0.001		
		2010–2015	0.56 (0.22, 0.91)	0.003		
		2015–2021	-0.24 (-0.45, -0.03)	0.030		
	ASMR	1990–1999	0.71 (0.50, 0.92)	<0.001	0.14 (-0.00, 0.28)	0.053
		1999–2012	-0.60 (-0.71, -0.50)	<0.001		
		2012–2016	2.50 (1.64, 3.36)	<0.001		
		2016–2021	-0.81 (-1.22, -0.41)	<0.001		
Low SDI	ASIR	1990–1992	1.61 (0.66, 2.57)	0.002	0.06 (-0.02, 0.14)	0.129
		1992–2000	0.45 (0.34, 0.57)	<0.001		
		2000–2005	-0.81 (-1.04, -0.58)	<0.001		
		2005–2015	0.06 (-0.00, 0.12)	0.062		
		2015–2021	-0.24 (-0.38, -0.11)	0.001		
	ASMR	1990–1998	0.29 (0.16, 0.42)	<0.001	-0.22 (-0.30, -0.13)	<0.001

Notes: The female-to-male ratio (FMR) represents the rate in females divided by the corresponding rate in males. Temporal trends were quantified using Joinpoint regression models. Abbreviations: APC, annual percentage change; AAPC, average annual percentage change; ASIR, age-standardized incidence rate; ASMR, age-standardized mortality rate; CI, confidence interval; FMR, female-to-male ratio; MDR-TB, multidrug-resistant tuberculosis; SDI, Socio-demographic Index. $P < 0.05$ indicates statistical significance.

Table S12. Female-to-male ratio point estimates for all-form TB and MDR-TB in 16 high-burden countries

Location	Disease	Metric	1990	1995	2000	2005	2010	2015	2021
Angola	All-form TB	ASIR	1.72 (0.89–2.55)	1.70 (0.88–2.52)	1.60 (0.84–2.37)	1.60 (0.83–2.37)	1.44 (0.74–2.14)	1.47 (0.75–2.19)	1.38 (0.71–2.05)
	MDR-TB		1.58 (0.00–5.83)	1.54 (0.00–5.33)	1.49 (0.00–5.09)	1.49 (0.00–4.92)	1.41 (0.00–4.56)	1.45 (0.00–4.57)	1.38 (0.00–4.30)
	All-form TB	ASMR	1.51 (0.28–2.73)	1.50 (0.34–2.67)	1.48 (0.38–2.58)	1.41 (0.44–2.37)	1.30 (0.42–2.18)	1.24 (0.38–2.10)	1.11 (0.29–1.93)
	MDR-TB		1.55 (0.00–6.04)	1.52 (0.00–5.47)	1.48 (0.00–5.22)	1.44 (0.00–5.00)	1.33 (0.00–4.53)	1.25 (0.00–4.11)	1.11 (0.00–3.80)
Bangladesh	All-form TB	ASIR	2.61 (1.19–4.04)	2.64 (1.20–4.08)	2.69 (1.22–4.16)	2.61 (1.56–3.67)	2.64 (1.56–3.71)	2.80 (1.24–4.37)	2.78 (1.23–4.33)
	MDR-TB		2.59 (0.00–10.12)	2.56 (0.00–9.50)	2.64 (0.00–8.16)	2.57 (0.00–6.44)	2.66 (0.36–4.96)	2.83 (0.00–6.20)	2.79 (0.00–7.64)
	All-form TB	ASMR	2.68 (0.81–4.55)	2.68 (0.92–4.44)	2.60 (0.96–4.23)	2.48 (1.00–3.96)	2.72 (1.05–4.40)	2.76 (1.10–4.41)	2.76 (1.05–4.48)
	MDR-TB		2.67 (0.00–11.35)	2.64 (0.00–10.42)	2.57 (0.00–8.53)	2.46 (0.00–7.00)	2.72 (0.00–6.34)	2.76 (0.00–6.82)	2.78 (0.00–7.76)
China	All-form TB	ASIR	1.11 (0.56–1.66)	1.16 (0.63–1.70)	1.14 (0.63–1.65)	1.14 (0.60–1.68)	1.12 (0.57–1.68)	1.16 (0.62–1.69)	1.16 (0.60–1.73)
	MDR-TB		1.12 (0.00–3.28)	1.17 (0.29–2.05)	1.14 (0.28–2.00)	1.12 (0.39–1.86)	1.12 (0.00–2.24)	1.15 (0.00–3.43)	1.15 (0.00–3.70)
	All-form TB	ASMR	1.14 (0.77–1.51)	1.10 (0.83–1.37)	1.05 (0.81–1.29)	0.92 (0.71–1.13)	0.83 (0.61–1.04)	0.77 (0.57–0.97)	0.75 (0.45–1.04)
	MDR-TB		1.15 (0.00–3.38)	1.11 (0.01–2.21)	1.04 (0.00–2.09)	0.91 (0.00–1.84)	0.82 (0.00–1.93)	0.77 (0.00–2.35)	0.74 (0.00–2.28)
Democratic Republic of the Congo	All-form TB	ASIR	1.97 (0.98–2.96)	1.94 (0.99–2.89)	1.83 (0.91–2.75)	1.86 (0.91–2.82)	1.69 (0.82–2.57)	1.73 (0.85–2.62)	1.58 (0.81–2.36)
	MDR-TB		1.74 (0.00–7.12)	1.77 (0.00–6.53)	1.76 (0.00–6.09)	1.81 (0.00–6.34)	1.66 (0.00–5.36)	1.70 (0.00–5.84)	1.58 (0.00–4.98)
	All-form TB	ASMR	1.65 (0.00–3.36)	1.60 (0.00–3.27)	1.59 (0.00–3.24)	1.56 (0.01–3.11)	1.48 (0.00–2.98)	1.40 (0.00–2.87)	1.28 (0.00–2.83)
	MDR-TB		1.64 (0.00–7.34)	1.59 (0.00–6.18)	1.60 (0.00–5.96)	1.58 (0.00–6.00)	1.50 (0.00–5.60)	1.40 (0.00–5.20)	1.28 (0.00–4.70)
India	All-form	ASIR	1.70 (0.64–	1.72 (0.85–	1.59 (0.76–	1.43 (0.72–	1.31 (0.63–	1.32 (0.66–	1.31 (0.63–

Location	Disease	Metric	1990	1995	2000	2005	2010	2015	2021
	TB		2.76)	2.58)	2.42)	2.13)	1.98)	1.98)	1.99)
	MDR-TB		1.65 (0.00–6.71)	1.68 (0.00–5.91)	1.58 (0.00–3.99)	1.43 (0.30–2.56)	1.32 (0.00–2.90)	1.35 (0.00–3.51)	1.35 (0.00–3.63)
	All-form TB	ASMR	1.42 (0.85–1.98)	1.39 (0.88–1.89)	1.37 (0.93–1.81)	1.29 (0.88–1.71)	1.19 (0.81–1.57)	1.17 (0.75–1.60)	1.20 (0.58–1.83)
	MDR-TB		1.43 (0.00–6.00)	1.41 (0.00–4.94)	1.41 (0.00–3.82)	1.32 (0.00–2.80)	1.22 (0.00–2.79)	1.23 (0.00–3.31)	1.24 (0.00–3.59)
Indonesia	All-form TB	ASIR	1.44 (0.71–2.17)	1.42 (0.71–2.14)	1.38 (0.69–2.06)	1.33 (0.68–1.98)	1.29 (0.67–1.91)	1.23 (0.64–1.82)	1.22 (0.64–1.79)
	MDR-TB		1.39 (0.00–5.59)	1.38 (0.00–5.71)	1.35 (0.00–5.63)	1.32 (0.00–5.12)	1.28 (0.00–4.73)	1.23 (0.00–4.35)	1.22 (0.00–4.46)
	All-form TB	ASMR	1.21 (0.54–1.89)	1.19 (0.61–1.76)	1.22 (0.68–1.76)	1.17 (0.72–1.61)	1.08 (0.72–1.44)	0.98 (0.63–1.33)	0.94 (0.56–1.33)
	MDR-TB		1.19 (0.00–4.96)	1.18 (0.00–4.80)	1.21 (0.00–5.08)	1.18 (0.00–4.57)	1.09 (0.00–4.00)	0.98 (0.00–3.65)	0.94 (0.00–3.54)
Mongolia	All-form TB	ASIR	1.40 (0.70–2.09)	1.42 (0.72–2.12)	1.37 (0.68–2.06)	1.36 (0.65–2.06)	1.36 (0.63–2.08)	1.29 (0.62–1.96)	1.33 (0.64–2.03)
	MDR-TB		1.25 (0.00–4.88)	1.27 (0.00–4.37)	1.29 (0.00–3.44)	1.33 (0.00–3.07)	1.35 (0.00–3.22)	1.27 (0.00–3.42)	1.31 (0.00–3.62)
	All-form TB	ASMR	1.09 (0.29–1.89)	1.01 (0.27–1.74)	1.01 (0.18–1.84)	1.18 (0.32–2.05)	1.05 (0.35–1.74)	0.87 (0.25–1.49)	0.82 (0.17–1.47)
	MDR-TB		1.08 (0.00–4.45)	1.02 (0.00–3.60)	1.01 (0.00–3.00)	1.18 (0.00–3.12)	1.05 (0.00–2.63)	0.87 (0.00–2.31)	0.82 (0.00–2.21)
Mozambique	All-form TB	ASIR	1.93 (0.98–2.88)	1.95 (0.94–2.95)	1.95 (0.77–3.14)	2.09 (0.65–3.53)	2.13 (0.47–3.78)	2.03 (0.59–3.47)	1.90 (0.65–3.16)
	MDR-TB		1.89 (0.00–6.31)	1.91 (0.00–4.50)	1.95 (0.19–3.72)	2.08 (0.26–3.91)	2.12 (0.03–4.21)	2.02 (0.00–4.10)	1.89 (0.00–5.10)
	All-form TB	ASMR	1.93 (0.54–3.32)	1.84 (0.58–3.11)	1.77 (0.56–2.98)	1.76 (0.69–2.83)	1.71 (0.77–2.65)	1.73 (0.77–2.68)	1.64 (0.56–2.73)
	MDR-TB		1.89 (0.00–7.12)	1.85 (0.00–4.97)	1.79 (0.00–4.22)	1.79 (0.00–3.97)	1.74 (0.00–3.85)	1.74 (0.00–3.89)	1.65 (0.00–4.68)
Myanmar	All-form TB	ASIR	1.15 (0.54–1.75)	1.12 (0.54–1.71)	1.15 (0.51–1.78)	1.14 (0.49–1.80)	1.14 (0.45–1.83)	1.10 (0.47–1.74)	1.02 (0.45–1.59)
	MDR-T		1.00 (0.00–	1.01 (0.00–	1.03 (0.00–	1.05 (0.32–	1.07 (0.30–	1.05 (0.18–	1.01 (0.00–

Location	Disease	Metric	1990	1995	2000	2005	2010	2015	2021
	B		3.85)	3.09)	2.18)	1.77)	1.84)	1.93)	2.57)
	All-form TB	ASMR	0.83 (0.21–1.45)	0.85 (0.25–1.46)	0.86 (0.28–1.44)	0.83 (0.27–1.39)	0.74 (0.23–1.26)	0.72 (0.21–1.24)	0.68 (0.17–1.19)
	MDR-TB		0.83 (0.00–3.22)	0.86 (0.00–2.89)	0.86 (0.00–2.16)	0.84 (0.00–1.83)	0.75 (0.00–1.64)	0.72 (0.00–1.66)	0.68 (0.00–1.90)
Nigeria	All-form TB	ASIR	1.28 (0.74–1.83)	1.29 (0.74–1.83)	1.32 (0.73–1.90)	1.29 (0.70–1.87)	1.30 (0.70–1.90)	1.26 (0.69–1.84)	1.29 (0.69–1.89)
	MDR-TB		1.20 (0.00–4.56)	1.24 (0.00–4.62)	1.30 (0.00–3.78)	1.30 (0.00–3.01)	1.30 (0.54–2.07)	1.26 (0.00–2.93)	1.29 (0.00–3.71)
	All-form TB	ASMR	1.02 (0.45–1.60)	1.08 (0.44–1.71)	1.14 (0.40–1.89)	1.11 (0.29–1.93)	1.08 (0.31–1.86)	1.12 (0.48–1.76)	1.10 (0.30–1.90)
	MDR-TB		1.01 (0.00–4.32)	1.07 (0.00–4.39)	1.14 (0.00–3.53)	1.12 (0.00–3.00)	1.08 (0.00–2.37)	1.12 (0.00–3.00)	1.11 (0.00–3.55)
Pakistan	All-form TB	ASIR	2.42 (1.21–3.63)	2.57 (1.26–3.88)	2.72 (1.30–4.14)	2.74 (1.33–4.15)	2.76 (1.31–4.21)	2.85 (1.43–4.28)	2.82 (1.40–4.25)
	MDR-TB		2.17 (0.00–8.37)	2.37 (0.00–9.45)	2.53 (0.00–9.02)	2.58 (0.00–7.30)	2.58 (0.00–5.41)	2.68 (0.44–4.91)	2.69 (0.00–7.13)
	All-form TB	ASMR	1.60 (0.28–2.93)	1.72 (0.43–3.00)	1.88 (0.55–3.21)	1.90 (0.65–3.15)	1.85 (0.73–2.96)	1.95 (0.77–3.13)	1.93 (0.77–3.09)
	MDR-TB		1.59 (0.00–6.61)	1.71 (0.00–6.81)	1.86 (0.00–6.57)	1.89 (0.00–5.63)	1.84 (0.00–4.65)	1.95 (0.00–4.41)	1.92 (0.00–5.51)
Papua New Guinea	All-form TB	ASIR	1.77 (0.82–2.73)	1.82 (0.83–2.82)	1.81 (0.83–2.79)	1.84 (0.85–2.84)	1.77 (0.80–2.74)	1.72 (0.77–2.66)	1.56 (0.70–2.42)
	MDR-TB		1.51 (0.00–6.10)	1.59 (0.00–5.92)	1.60 (0.00–5.75)	1.65 (0.00–4.93)	1.62 (0.00–3.67)	1.62 (0.40–2.83)	1.49 (0.00–3.77)
	All-form TB	ASMR	0.97 (0.00–1.96)	0.97 (0.04–1.90)	1.01 (0.08–1.94)	1.03 (0.11–1.96)	0.97 (0.13–1.81)	0.91 (0.13–1.69)	0.85 (0.15–1.55)
	MDR-TB		0.94 (0.00–3.87)	0.95 (0.00–3.95)	1.02 (0.00–3.78)	1.04 (0.00–3.51)	0.98 (0.00–2.60)	0.93 (0.00–2.14)	0.87 (0.00–2.49)
Philippines	All-form TB	ASIR	0.97 (0.51–1.43)	0.90 (0.47–1.34)	0.95 (0.49–1.42)	1.05 (0.53–1.57)	1.41 (0.70–2.12)	1.00 (0.52–1.49)	0.98 (0.48–1.47)
	MDR-TB		0.95 (0.00–3.40)	0.89 (0.00–3.47)	0.94 (0.00–3.13)	1.03 (0.00–2.94)	1.37 (0.15–2.58)	1.01 (0.00–2.01)	0.98 (0.00–2.61)
	All-form	ASMR	0.95 (0.46–	0.87 (0.46–	0.87 (0.55–	0.90 (0.64–	0.88 (0.65–	0.83 (0.59–	0.87 (0.58–

Location	Disease	Metric	1990	1995	2000	2005	2010	2015	2021
	TB		1.45)	1.28)	1.18)	1.15)	1.11)	1.08)	1.15)
	MDR-TB		0.97 (0.00–3.67)	0.88 (0.00–3.22)	0.87 (0.00–2.91)	0.90 (0.00–2.63)	0.88 (0.00–1.86)	0.83 (0.00–1.91)	0.86 (0.00–2.43)
South Africa	All-form TB	ASIR	1.62 (0.94–2.29)	1.47 (0.82–2.12)	1.46 (0.75–2.17)	1.57 (0.67–2.48)	1.71 (0.64–2.79)	1.49 (0.64–2.34)	1.41 (0.69–2.13)
	MDR-TB		1.60 (0.00–5.05)	1.47 (0.00–3.44)	1.48 (0.00–3.81)	1.59 (0.00–3.95)	1.73 (0.00–3.70)	1.48 (0.36–2.61)	1.40 (0.00–3.91)
	All-form TB	ASMR	0.96 (0.39–1.53)	0.95 (0.61–1.29)	0.93 (0.69–1.17)	0.98 (0.73–1.22)	0.97 (0.72–1.22)	0.93 (0.66–1.20)	0.95 (0.57–1.32)
	MDR-TB		0.96 (0.00–3.29)	0.96 (0.00–2.62)	0.95 (0.00–2.56)	1.01 (0.00–2.73)	0.99 (0.00–2.36)	0.93 (0.00–1.96)	0.95 (0.00–2.65)
Viet Nam	All-form TB	ASIR	1.79 (0.88–2.70)	1.81 (0.90–2.71)	1.73 (0.86–2.60)	1.69 (0.91–2.48)	1.69 (0.88–2.50)	1.78 (0.87–2.70)	1.72 (0.85–2.59)
	MDR-TB		1.75 (0.00–6.03)	1.75 (0.00–3.78)	1.71 (0.00–3.51)	1.71 (0.31–3.10)	1.71 (0.61–2.82)	1.80 (0.10–3.50)	1.73 (0.00–4.66)
	All-form TB	ASMR	1.80 (0.75–2.86)	1.55 (0.70–2.39)	1.35 (0.66–2.03)	1.41 (0.76–2.06)	1.42 (0.72–2.12)	1.46 (0.65–2.27)	1.44 (0.48–2.40)
	MDR-TB		1.81 (0.00–6.19)	1.55 (0.00–3.82)	1.35 (0.00–3.17)	1.43 (0.00–3.22)	1.45 (0.00–3.04)	1.48 (0.00–3.43)	1.44 (0.00–4.21)
Zambia	All-form TB	ASIR	2.03 (0.92–3.15)	2.19 (0.82–3.56)	2.31 (0.63–3.98)	2.33 (0.45–4.22)	2.09 (0.53–3.64)	1.92 (0.57–3.27)	1.80 (0.65–2.95)
	MDR-TB		2.08 (0.00–7.07)	2.08 (0.00–5.82)	2.25 (0.00–5.06)	2.29 (0.00–5.39)	2.08 (0.00–4.73)	1.94 (0.00–5.45)	1.81 (0.00–5.74)
	All-form TB	ASMR	1.98 (0.61–3.35)	1.90 (0.60–3.20)	1.84 (0.56–3.11)	1.74 (0.50–2.98)	1.75 (0.45–3.05)	1.85 (0.30–3.39)	1.83 (0.05–3.61)
	MDR-TB		2.02 (0.00–7.03)	1.94 (0.00–5.85)	1.88 (0.00–4.77)	1.80 (0.00–4.65)	1.78 (0.00–4.49)	1.86 (0.00–5.48)	1.85 (0.00–6.18)

Notes: The female-to-male ratio (FMR) represents the rate in females divided by the corresponding rate in males. Values represent the point estimate followed by the 95% uncertainty interval in parentheses. Abbreviations: ASIR, age-standardized incidence rate; ASMR, age-standardized mortality rate; FMR, female-to-male ratio; TB, tuberculosis; MDR-TB, multidrug-resistant tuberculosis.

Table S13. Joinpoint regression analysis of FMR for pediatric all-form TB in 16 high-burden countries

Location	Metric	Periods	APC (95% CI) (%)	P value (APC)	AAPC (95% CI) (%)	P value (AAPC)
Angola	ASIR	1990–2006	-0.62 (-0.78, -0.46)	<0.001	-0.84 (-1.19, -0.49)	<0.001
		2006–2010	-2.29 (-4.27, -0.27)	0.028		
		2010–2015	0.58 (-0.73, 1.90)	0.372		
		2015–2021	-1.64 (-2.34, -0.94)	<0.001		
	ASMR	1990–1994	0.05 (-0.07, 0.16)	0.387	-0.97 (-1.00, -0.95)	<0.001
		1994–2003	-0.41 (-0.44, -0.37)	<0.001		
		2003–2010	-1.66 (-1.71, -1.61)	<0.001		
		2010–2016	-0.92 (-0.99, -0.86)	<0.001		
		2016–2021	-1.89 (-1.96, -1.82)	<0.001		
Bangladesh	ASIR	1990–2000	0.32 (0.27, 0.37)	<0.001	0.20 (0.15, 0.25)	<0.001
		2000–2005	-0.62 (-0.78, -0.45)	<0.001		
		2005–2010	0.16 (0.02, 0.30)	0.024		
		2010–2017	1.17 (1.07, 1.26)	<0.001		
		2017–2021	-0.72 (-0.93, -0.52)	<0.001		
	ASMR	1990–1997	-0.13 (-0.53, 0.28)	0.521	0.13 (-0.16, 0.41)	0.381
		1997–2006	-0.90 (-1.20, -0.60)	<0.001		
		2006–2009	3.15 (0.36, 6.02)	0.028		
		2009–2021	0.30 (0.14, 0.47)	<0.001		
China	ASIR	1990–1996	0.88 (0.71, 1.06)	<0.001	0.12 (0.00, 0.24)	0.042
		1996–2001	-0.59 (-0.90, -0.28)	<0.001		
		2001–2011	-0.11 (-0.20, -0.01)	0.027		
		2011–2014	0.74 (-0.31, 1.79)	0.156		
		2014–2021	0.04 (-0.09, 0.18)	0.501		
	ASMR	1990–2001	-0.69 (-0.83, -0.55)	<0.001	-1.36 (-1.59, -1.12)	<0.001
		2001–2007	-3.30 (-3.70, -2.91)	<0.001		
		2007–2014	-1.58 (-1.90, -1.25)	<0.001		
		2014–2017	0.59 (-1.44, 2.67)	0.549		
		2017–2021	-1.28 (-2.13, -0.43)	0.005		
Democratic Republic of the Congo	ASIR	1990–2007	-0.52 (-0.65, -0.38)	<0.001	-0.75 (-1.31, -0.18)	0.009
		2007–2010	-2.23 (-5.97, 1.66)	0.241		
		2010–2016	0.43 (-0.45, 1.32)	0.323		
		2016–2019	-3.97 (-7.50, -0.31)	0.035		
		2019–2021	0.93 (-2.92, 4.94)	0.623		

Location	Metric	Periods	APC (95% CI) (%)	P value (APC)	AAPC (95% CI) (%)	P value (AAPC)
	ASMR	1990–1992	0.05 (-0.64, 0.74)	0.891	-0.83 (-0.89, -0.76)	<0.001
		1992–1996	-1.08 (-1.42, -0.73)	<0.001		
		1996–2003	0.08 (-0.04, 0.20)	0.168		
		2003–2014	-1.02 (-1.07, -0.97)	<0.001		
		2014–2021	-1.52 (-1.62, -1.42)	<0.001		
India	ASIR	1990–1996	0.23 (0.10, 0.37)	0.002	-0.84 (-0.92, -0.76)	<0.001
		1996–2010	-2.04 (-2.08, -2.01)	<0.001		
		2010–2015	0.45 (0.22, 0.68)	<0.001		
		2015–2019	-0.72 (-1.08, -0.35)	<0.001		
		2019–2021	1.01 (0.27, 1.76)	0.011		
	ASMR	1990–2008	-0.81 (-0.99, -0.62)	<0.001	-0.55 (-1.08, -0.02)	0.042
		2008–2011	-2.64 (-7.75, 2.76)	0.317		
		2011–2021	0.54 (-0.01, 1.10)	0.055		
Indonesia	ASIR	1990–1994	-0.16 (-0.23, -0.10)	<0.001	-0.55 (-0.58, -0.53)	<0.001
		1994–2010	-0.67 (-0.68, -0.66)	<0.001		
		2010–2015	-0.96 (-1.02, -0.90)	<0.001		
		2015–2018	-0.50 (-0.70, -0.31)	<0.001		
		2018–2021	0.18 (0.08, 0.28)	0.001		
	ASMR	1990–1993	-0.94 (-1.95, 0.07)	0.065	-0.79 (-0.93, -0.66)	<0.001
		1993–2001	0.48 (0.24, 0.71)	<0.001		
		2001–2009	-1.31 (-1.49, -1.13)	<0.001		
		2009–2014	-2.17 (-2.56, -1.78)	<0.001		
		2014–2021	-0.59 (-0.78, -0.40)	<0.001		
Mongolia	ASIR	1990–1994	0.48 (0.19, 0.78)	0.003	-0.14 (-0.22, -0.07)	<0.001
		1994–2002	-0.62 (-0.74, -0.50)	<0.001		
		2002–2010	0.02 (-0.10, 0.15)	0.718		
		2010–2017	-1.01 (-1.17, -0.85)	<0.001		
		2017–2021	1.40 (1.09, 1.70)	<0.001		
	ASMR	1990–2000	-0.69 (-1.37, -0.01)	0.046	-0.98 (-1.63, -0.33)	0.003
		2000–2004	4.63 (-0.39, 9.90)	0.069		
		2004–2021	-2.42 (-2.71, -2.13)	<0.001		
Mozambique	ASIR	1990–2000	0.09 (0.04, 0.15)	0.002	-0.04 (-0.10, 0.03)	0.275
		2000–2005	1.40 (1.13, 1.67)	<0.001		
		2005–2011	0.29 (0.07, 0.51)	0.011		

Location	Metric	Periods	APC (95% CI) (%)	P value (APC)	AAPC (95% CI) (%)	P value (AAPC)
		2011–2021	-1.06 (-1.14, -0.99)	<0.001		
	ASMR	1990–2002	-0.90 (-0.95, -0.86)	<0.001	-0.53 (-0.59, -0.46)	<0.001
		2002–2007	0.54 (0.32, 0.75)	<0.001		
		2007–2011	-1.26 (-1.55, -0.97)	<0.001		
		2011–2016	0.31 (0.12, 0.49)	0.003		
		2016–2021	-0.91 (-1.06, -0.76)	<0.001		
Myanmar	ASIR	1990–1995	-0.43 (-0.56, -0.30)	<0.001	-0.37 (-0.42, -0.31)	<0.001
		1995–2001	0.44 (0.30, 0.58)	<0.001		
		2001–2013	-0.13 (-0.17, -0.08)	<0.001		
		2013–2019	-1.63 (-1.77, -1.48)	<0.001		
		2019–2021	-0.26 (-0.91, 0.40)	0.423		
	ASMR	1990–1998	0.54 (0.39, 0.69)	<0.001	-0.64 (-0.76, -0.52)	<0.001
		1998–2005	-0.65 (-0.86, -0.43)	<0.001		
		2005–2012	-2.07 (-2.28, -1.85)	<0.001		
		2012–2016	0.49 (-0.16, 1.14)	0.131		
		2016–2021	-1.39 (-1.70, -1.09)	<0.001		
Nigeria	ASIR	1990–2002	0.25 (0.20, 0.31)	<0.001	0.03 (-0.09, 0.14)	0.653
		2002–2005	-0.79 (-1.72, 0.15)	0.096		
		2005–2010	0.28 (-0.03, 0.59)	0.070		
		2010–2016	-0.54 (-0.76, -0.33)	<0.001		
		2016–2021	0.39 (0.18, 0.61)	0.001		
	ASMR	1990–2001	1.21 (1.13, 1.29)	<0.001	0.28 (0.20, 0.36)	<0.001
		2001–2009	-0.91 (-1.09, -0.72)	<0.001		
		2009–2017	0.60 (0.43, 0.76)	<0.001		
		2017–2021	-0.50 (-0.91, -0.08)	0.021		
Pakistan	ASIR	1990–2000	1.16 (1.08, 1.25)	<0.001	0.50 (0.43, 0.57)	<0.001
		2000–2009	0.08 (-0.05, 0.20)	0.219		
		2009–2015	0.60 (0.35, 0.86)	<0.001		
		2015–2021	-0.06 (-0.24, 0.13)	0.541		
	ASMR	1990–1999	1.79 (1.61, 1.97)	<0.001	0.65 (0.50, 0.81)	<0.001
		1999–2004	0.53 (-0.03, 1.09)	0.063		
		2004–2011	-0.51 (-0.78, -0.24)	<0.001		
		2011–2015	1.63 (0.84, 2.43)	<0.001		
		2015–2021	-0.22 (-0.48, 0.03)	0.085		

Location	Metric	Periods	APC (95% CI) (%)	P value (APC)	AAPC (95% CI) (%)	P value (AAPC)
Papua New Guinea	ASIR	1990–2005	0.18 (0.11, 0.24)	<0.001	-0.43 (-0.60, -0.26)	<0.001
		2005–2015	-0.68 (-0.82, -0.53)	<0.001		
		2015–2018	-2.66 (-4.20, -1.11)	0.002		
		2018–2021	-0.37 (-1.19, 0.46)	0.365		
	ASMR	1990–1994	-0.13 (-0.83, 0.58)	0.712	-0.42 (-0.56, -0.28)	<0.001
		1994–2006	0.55 (0.41, 0.69)	<0.001		
		2006–2018	-1.45 (-1.58, -1.33)	<0.001		
		2018–2021	-0.52 (-1.42, 0.40)	0.252		
Philippines	ASIR	1990–1995	-1.51 (-1.83, -1.18)	<0.001	0.03 (-0.11, 0.17)	0.698
		1995–2005	1.42 (1.28, 1.56)	<0.001		
		2005–2010	6.75 (6.24, 7.26)	<0.001		
		2010–2014	-8.17 (-8.85, -7.49)	<0.001		
		2014–2021	-0.60 (-0.80, -0.40)	<0.001		
	ASMR	1990–2001	-0.94 (-1.31, -0.56)	<0.001	-0.26 (-0.63, 0.11)	0.163
		2001–2008	1.13 (0.48, 1.79)	0.002		
		2008–2011	-3.07 (-6.23, 0.20)	0.064		
		2011–2021	0.38 (0.08, 0.67)	0.014		
South Africa	ASIR	1990–1992	2.58 (1.05, 4.12)	0.002	-0.44 (-0.59, -0.28)	<0.001
		1992–2004	0.80 (0.68, 0.93)	<0.001		
		2004–2019	-1.86 (-1.95, -1.77)	<0.001		
		2019–2021	-0.01 (-1.75, 1.75)	0.986		
	ASMR	1990–2001	-0.72 (-0.76, -0.67)	<0.001	-0.24 (-0.32, -0.17)	<0.001
		2001–2005	-1.17 (-1.53, -0.80)	<0.001		
		2005–2010	0.08 (-0.15, 0.32)	0.472		
		2010–2017	1.11 (0.97, 1.26)	<0.001		
		2017–2021	-0.75 (-1.06, -0.45)	<0.001		
Viet Nam	ASIR	1990–1994	-2.28 (-2.56, -2.00)	<0.001	-0.46 (-0.55, -0.38)	<0.001
		1994–2001	-0.10 (-0.27, 0.07)	0.225		
		2001–2010	1.92 (1.79, 2.06)	<0.001		
		2010–2016	-2.90 (-3.17, -2.61)	<0.001		
		2016–2021	-0.80 (-1.05, -0.54)	<0.001		
	ASMR	1990–2002	-0.23 (-0.38, -0.08)	0.005	-0.05 (-0.26, 0.17)	0.685
		2002–2005	1.66 (-0.06, 3.40)	0.057		
		2005–2012	-0.15 (-0.43, 0.12)	0.261		

Location	Metric	Periods	APC (95% CI) (%)	P value (APC)	AAPC (95% CI) (%)	P value (AAPC)
		2012–2016	-1.56 (-2.45, -0.65)	0.002		
		2016–2021	0.76 (0.25, 1.29)	0.006		
Zambia	ASIR	1990–1995	0.18 (0.01, 0.35)	0.036	-0.13 (-0.18, -0.07)	<0.001
		1995–2001	-0.91 (-1.07, -0.74)	<0.001		
		2001–2009	-0.30 (-0.39, -0.21)	<0.001		
		2009–2016	0.98 (0.85, 1.11)	<0.001		
		2016–2021	-0.75 (-0.92, -0.58)	<0.001		
	ASMR	1990–1998	-3.10 (-3.25, -2.94)	<0.001	-0.73 (-0.92, -0.54)	<0.001
		1998–2001	-2.07 (-3.41, -0.72)	0.005		
		2001–2004	1.90 (0.54, 3.28)	0.009		
		2004–2017	0.38 (0.30, 0.46)	<0.001		
		2017–2021	-0.50 (-1.04, 0.03)	0.064		

Notes: The female-to-male ratio (FMR) represents the rate in females divided by the corresponding rate in males. Temporal trends were quantified using Joinpoint regression models. Abbreviations: APC, annual percentage change; AAPC, average annual percentage change; ASIR, age-standardized incidence rate; ASMR, age-standardized mortality rate; CI, confidence interval; FMR, female-to-male ratio. $P < 0.05$ indicates statistical significance.

Table S14. Joinpoint regression analysis of FMR for pediatric MDR-TB in 16 high-burden countries

Location	Metric	Periods	APC (95% CI) (%)	P value (APC)	AAPC (95% CI) (%)	P value (AAPC)
Angola	ASIR	1990–2012	-0.54 (-0.64, -0.44)	<0.001	-0.56 (-0.93, -0.18)	0.004
		2012–2015	1.01 (-2.77, 4.92)	0.592		
		2015–2021	-1.38 (-2.02, -0.73)	<0.001		
	ASMR	1990–1998	-0.60 (-0.70, -0.51)	<0.001	-1.11 (-1.17, -1.04)	<0.001
		1998–2004	-0.29 (-0.47, -0.11)	0.004		
		2004–2011	-1.59 (-1.72, -1.45)	<0.001		
		2011–2016	-1.22 (-1.45, -0.99)	<0.001		
		2016–2021	-2.09 (-2.26, -1.93)	<0.001		
Bangladesh	ASIR	1990–2001	0.19 (0.07, 0.32)	0.004	0.25 (0.14, 0.37)	<0.001
		2001–2007	-0.43 (-0.68, -0.19)	0.001		
		2007–2014	1.33 (1.22, 1.43)	<0.001		
		2014–2017	0.58 (-0.29, 1.46)	0.177		
		2017–2021	-0.65 (-1.02, -0.29)	0.002		
	ASMR	1990–2006	-0.63 (-0.79, -0.46)	<0.001	0.11 (-0.15, 0.38)	0.405

Location	Metric	Periods	APC (95% CI) (%)	P value (APC)	AAPC (95% CI) (%)	P value (AAPC)
		2006–2009	3.05 (0.35, 5.83)	0.028		
		2009–2021	0.37 (0.22, 0.53)	<0.001		
China	ASIR	1990–1992	1.76 (0.33, 3.20)	0.018	0.11 (0.00, 0.21)	0.040
		1992–1996	0.19 (-0.16, 0.54)	0.268		
		1996–2001	-0.73 (-0.92, -0.54)	<0.001		
		2001–2009	-0.18 (-0.26, -0.11)	<0.001		
		2009–2021	0.36 (0.28, 0.44)	<0.001		
	ASMR	1990–1996	-0.63 (-1.03, -0.23)	0.004	-1.35 (-1.53, -1.17)	<0.001
		1996–2002	-1.21 (-1.59, -0.83)	<0.001		
		2002–2007	-3.55 (-4.10, -3.01)	<0.001		
		2007–2013	-1.39 (-1.89, -0.89)	<0.001		
		2013–2021	-0.56 (-0.93, -0.18)	0.006		
Democratic Republic of the Congo	ASIR	1990–1998	-0.72 (-1.43, 0.00)	0.051	-0.60 (-1.08, -0.11)	0.016
		1998–2005	0.64 (-0.36, 1.65)	0.194		
		2005–2010	-1.84 (-3.55, -0.10)	0.040		
		2010–2015	0.50 (-1.20, 2.23)	0.547		
		2015–2021	-1.74 (-2.66, -0.82)	<0.001		
	ASMR	1990–1992	0.33 (-0.88, 1.54)	0.578	-0.80 (-0.91, -0.70)	<0.001
		1992–1997	-1.13 (-1.45, -0.82)	<0.001		
		1997–2002	0.65 (0.34, 0.96)	<0.001		
		2002–2011	-0.82 (-0.92, -0.71)	<0.001		
		2011–2021	-1.57 (-1.64, -1.50)	<0.001		
India	ASIR	1990–1992	2.72 (-3.08, 8.86)	0.351	-0.71 (-1.08, -0.35)	<0.001
		1992–2010	-1.64 (-1.73, -1.54)	<0.001		
		2010–2021	0.19 (0.00, 0.38)	0.045		
	ASMR	1990–2008	-0.66 (-0.91, -0.42)	<0.001	-0.45 (-0.95, 0.06)	0.085
		2008–2011	-2.46 (-7.24, 2.55)	0.315		
		2011–2021	0.57 (0.04, 1.10)	0.036		
Indonesia	ASIR	1990–1992	1.04 (0.49, 1.58)	<0.001	-0.43 (-0.50, -0.37)	<0.001
		1992–1995	-1.00 (-1.56, -0.44)	0.001		
		1995–2007	-0.43 (-0.47, -0.39)	<0.001		
		2007–2017	-0.77 (-0.81, -0.73)	<0.001		
		2017–2021	0.09 (-0.06, 0.25)	0.210		
	ASMR	1990–1993	-0.73 (-1.63, 0.18)	0.108	-0.73 (-0.87, -0.59)	<0.001

Location	Metric	Periods	APC (95% CI) (%)	P value (APC)	AAPC (95% CI) (%)	P value (AAPC)
		1993–2000	0.71 (0.40, 1.01)	<0.001		
		2000–2008	-0.94 (-1.15, -0.72)	<0.001		
		2008–2014	-2.22 (-2.55, -1.89)	<0.001		
		2014–2021	-0.64 (-0.85, -0.44)	<0.001		
Mongolia	ASIR	1990–1992	2.68 (-0.12, 5.56)	0.059	0.13 (-0.16, 0.43)	0.374
		1992–1995	-1.82 (-4.27, 0.69)	0.144		
		1995–2009	0.55 (0.47, 0.63)	<0.001		
		2009–2016	-1.21 (-1.46, -0.96)	<0.001		
		2016–2021	1.04 (0.66, 1.42)	<0.001		
	ASMR	1990–2000	-0.62 (-1.22, -0.02)	0.043	-0.88 (-1.56, -0.19)	0.012
		2000–2003	5.34 (-0.95, 12.03)	0.093		
		2003–2011	-1.52 (-2.16, -0.87)	<0.001		
		2011–2015	-4.41 (-6.66, -2.10)	<0.001		
		2015–2021	-1.07 (-1.91, -0.22)	0.016		
Mozambique	ASIR	1990–1998	-0.10 (-0.32, 0.12)	0.347	-0.04 (-0.19, 0.10)	0.536
		1998–2002	0.83 (0.23, 1.44)	0.009		
		2002–2006	1.63 (1.02, 2.24)	<0.001		
		2006–2010	0.15 (-0.45, 0.75)	0.603		
		2010–2021	-0.99 (-1.11, -0.88)	<0.001		
	ASMR	1990–2003	-0.61 (-0.64, -0.57)	<0.001	-0.45 (-0.50, -0.40)	<0.001
		2003–2007	0.80 (0.56, 1.03)	<0.001		
		2007–2011	-1.36 (-1.58, -1.14)	<0.001		
		2011–2015	0.32 (0.08, 0.55)	0.010		
		2015–2021	-0.83 (-0.93, -0.73)	<0.001		
Myanmar	ASIR	1990–1996	-0.91 (-2.17, 0.37)	0.155	-0.19 (-0.44, 0.07)	0.146
		1996–2010	0.47 (0.31, 0.62)	<0.001		
		2010–2021	-0.62 (-0.83, -0.41)	<0.001		
	ASMR	1990–1998	0.55 (0.29, 0.82)	<0.001	-0.68 (-0.81, -0.55)	<0.001
		1998–2005	-0.65 (-0.88, -0.41)	<0.001		
		2005–2012	-2.05 (-2.24, -1.85)	<0.001		
		2012–2016	0.25 (-0.35, 0.87)	0.394		
		2016–2021	-1.50 (-1.84, -1.15)	<0.001		
Nigeria	ASIR	1990–1999	0.70 (0.47, 0.92)	<0.001	0.21 (0.11, 0.30)	<0.001
		1999–2011	0.05 (-0.02, 0.13)	0.135		

Location	Metric	Periods	APC (95% CI) (%)	P value (APC)	AAPC (95% CI) (%)	P value (AAPC)
		2011–2015	-0.79 (-1.21, -0.37)	<0.001		
		2015–2021	0.44 (0.17, 0.72)	0.003		
	ASMR	1990–2001	1.25 (1.17, 1.32)	<0.001	0.31 (0.23, 0.38)	<0.001
		2001–2010	-0.76 (-0.83, -0.70)	<0.001		
		2010–2015	0.86 (0.68, 1.03)	<0.001		
		2015–2019	0.07 (-0.27, 0.42)	0.657		
		2019–2021	-0.91 (-1.72, -0.08)	0.033		
Pakistan	ASIR	1990–1992	4.27 (0.42, 8.26)	0.031	0.67 (0.41, 0.93)	<0.001
		1992–2004	0.63 (0.42, 0.84)	<0.001		
		2004–2010	-0.04 (-0.46, 0.39)	0.857		
		2010–2014	0.85 (0.35, 1.36)	0.002		
		2014–2021	0.22 (0.01, 0.42)	0.039		
	ASMR	1990–1999	1.77 (1.57, 1.97)	<0.001	0.66 (0.54, 0.78)	<0.001
		1999–2004	0.59 (0.06, 1.11)	0.030		
		2004–2011	-0.50 (-0.71, -0.30)	<0.001		
		2011–2015	1.61 (1.15, 2.06)	<0.001		
		2015–2021	-0.20 (-0.39, -0.01)	0.038		
Papua New Guinea	ASIR	1990–1992	6.61 (-1.58, 15.49)	0.110	-0.14 (-0.83, 0.55)	0.688
		1992–1996	-2.93 (-6.50, 0.78)	0.114		
		1996–2006	0.81 (0.24, 1.38)	0.008		
		2006–2016	-0.57 (-0.83, -0.30)	<0.001		
		2016–2021	-1.53 (-2.25, -0.81)	<0.001		
	ASMR	1990–2005	0.77 (0.63, 0.90)	<0.001	-0.20 (-0.32, -0.08)	<0.001
		2005–2018	-1.27 (-1.39, -1.16)	<0.001		
		2018–2021	-0.33 (-1.29, 0.64)	0.483		
Philippines	ASIR	1990–1992	-3.78 (-6.13, -1.37)	0.004	0.08 (-0.10, 0.25)	0.383
		1992–2004	0.92 (0.76, 1.07)	<0.001		
		2004–2010	6.12 (5.81, 6.43)	<0.001		
		2010–2014	-7.42 (-7.73, -7.11)	<0.001		
		2014–2021	-0.80 (-0.96, -0.64)	<0.001		
	ASMR	1990–2001	-1.07 (-1.48, -0.66)	<0.001	-0.33 (-0.67, 0.00)	0.053
		2001–2008	1.16 (0.41, 1.90)	0.004		
		2008–2011	-3.11 (-5.76, -0.40)	0.027		
		2011–2021	0.30 (0.05, 0.56)	0.023		

Location	Metric	Periods	APC (95% CI) (%)	P value (APC)	AAPC (95% CI) (%)	P value (AAPC)
South Africa	ASIR	1990–1994	-0.29 (-1.01, 0.43)	0.410	-0.52 (-0.66, -0.38)	<0.001
		1994–2000	1.60 (1.25, 1.95)	<0.001		
		2000–2005	0.50 (0.09, 0.92)	0.021		
		2005–2011	-2.02 (-2.31, -1.73)	<0.001		
		2011–2021	-1.46 (-1.61, -1.31)	<0.001		
	ASMR	1990–2000	-0.61 (-0.68, -0.55)	<0.001	-0.26 (-0.32, -0.19)	<0.001
		2000–2005	-1.03 (-1.24, -0.82)	<0.001		
		2005–2011	-0.08 (-0.22, 0.06)	0.256		
		2011–2017	1.04 (0.86, 1.22)	<0.001		
		2017–2021	-0.59 (-0.89, -0.29)	<0.001		
Viet Nam	ASIR	1990–1993	-2.42 (-3.52, -1.30)	<0.001	-0.46 (-0.60, -0.32)	<0.001
		1993–2000	-0.17 (-0.44, 0.10)	0.203		
		2000–2010	1.87 (1.72, 2.02)	<0.001		
		2010–2015	-3.33 (-3.63, -3.03)	<0.001		
		2015–2021	-1.23 (-1.49, -0.98)	<0.001		
	ASMR	1990–2002	0.03 (-0.11, 0.18)	0.652	-0.02 (-0.26, 0.22)	0.878
		2002–2005	1.68 (-0.51, 3.93)	0.126		
		2005–2012	-0.41 (-0.71, -0.10)	0.012		
		2012–2016	-1.65 (-2.35, -0.94)	<0.001		
		2016–2021	0.71 (0.28, 1.15)	0.003		
Zambia	ASIR	1990–1992	0.53 (-0.77, 1.86)	0.405	-0.03 (-0.12, 0.06)	0.543
		1992–2000	-0.55 (-0.64, -0.46)	<0.001		
		2000–2011	0.07 (0.03, 0.10)	0.001		
		2011–2015	1.33 (1.13, 1.53)	<0.001		
		2015–2021	-0.59 (-0.73, -0.45)	<0.001		
	ASMR	1990–1998	-3.16 (-3.37, -2.95)	<0.001	-0.75 (-0.93, -0.57)	<0.001
		1998–2001	-1.99 (-3.42, -0.53)	0.010		
		2001–2005	1.80 (1.08, 2.54)	<0.001		
		2005–2016	0.40 (0.30, 0.50)	<0.001		
		2016–2021	-0.65 (-1.06, -0.23)	0.004		

Notes: The female-to-male ratio (FMR) represents the rate in females divided by the corresponding rate in males. Temporal trends were quantified using Joinpoint regression models. Abbreviations: APC, annual percentage change; AAPC, average annual percentage change; ASIR, age-standardized incidence rate; ASMR, age-standardized mortality rate; CI, confidence interval; FMR, female-to-male ratio; MDR-TB, multidrug-resistant tuberculosis. $P < 0.05$ indicates statistical significance.

Table S15. Sensitivity analysis of temporal trends in the multidrug-resistant tuberculosis to all-form tuberculosis ratio (MAR) among children (0–14 years), globally and by Socio-demographic Index (SDI), restricted to the 2000–2021 period

Measure	Location	Annual Slope (Post-2000)	P-value	Trend Direction
Incidence	Global	0.0273	<0.001	Increasing
	High SDI	-0.0603	<0.001	Decreasing
	High-middle SDI	-0.0271	0.039	Decreasing
	Middle SDI	-0.054	<0.001	Decreasing
	Low-middle SDI	0.0771	<0.001	Increasing
	Low SDI	0.0515	<0.001	Increasing
Deaths	Global	0.1004	<0.001	Increasing
	High SDI	-0.207	<0.001	Decreasing
	High-middle SDI	-0.2865	<0.001	Decreasing
	Middle SDI	-0.0818	<0.001	Decreasing
	Low-middle SDI	0.1562	<0.001	Increasing
	Low SDI	0.1234	<0.001	Increasing
DALYs	Global	0.0916	<0.001	Increasing
	High SDI	-0.2142	<0.001	Decreasing
	High-middle SDI	-0.2832	<0.001	Decreasing
	Middle SDI	-0.0934	<0.001	Decreasing
	Low-middle SDI	0.1449	<0.001	Increasing
	Low SDI	0.1163	<0.001	Increasing

Note: Annual slopes represent the absolute yearly change in MAR (%) calculated using ordinary least squares regression restricted to the period 2000–2021. This sensitivity analysis was conducted to assess whether the observed long-term divergence between MDR-TB and all-form TB trajectories remained robust when excluding data from the 1990s, a period characterized by lower data quality and preceding the widespread implementation of rapid molecular diagnostics (e.g., GeneXpert MTB/RIF). The results suggested that the global MAR trend remained positive across incidence, deaths, and DALYs during 2000–2021. Increases were most evident in Low and Low-middle SDI

regions, whereas decreasing trends were observed in higher-SDI settings. These findings support the descriptive consistency of the main MAR patterns after excluding the 1990s.

Table S16. Sensitivity analysis of temporal trends in the multidrug-resistant tuberculosis to all-form tuberculosis ratio (MAR) among children (0–14 years), using count-based absolute cases and deaths as an alternative definition, 1990–2021

Measure	Location	Annual Slope (Count-based)	P-value	Trend Direction
Incidence	Global	0.0937	<0.001	Increasing
	High SDI	-0.0119	0.281	Decreasing
	High-middle SDI	0.1214	<0.001	Increasing
	Middle SDI	0.0307	0.05	Increasing
	Low-middle SDI	0.1494	<0.001	Increasing
	Low SDI	0.1019	<0.001	Increasing
	Central Asia	0.9061	<0.001	Increasing
	Eastern Europe	0.9484	<0.001	Increasing
	South Asia	0.224	<0.001	Increasing
	Southeast Asia	0.0256	0.004	Increasing
	Western Sub-Saharan Africa	0.0865	<0.001	Increasing
	East Asia	-0.1874	<0.001	Decreasing
Deaths	Global	0.2138	<0.001	Increasing
	High SDI	0.0081	0.809	Increasing
	High-middle SDI	0.0073	0.884	Increasing
	Middle SDI	0.0623	0.024	Increasing
	Low-middle SDI	0.2955	<0.001	Increasing
	Low SDI	0.2223	<0.001	Increasing
	Central Asia	1.0363	<0.001	Increasing
	Eastern Europe	0.8015	<0.001	Increasing
	South Asia	0.409	<0.001	Increasing
	Southeast Asia	0.0471	0.044	Increasing
	Western Sub-Saharan Africa	0.1935	<0.001	Increasing
	East Asia	-0.2778	<0.001	Decreasing

Note: Annual slopes represent the absolute yearly change in MAR (%) calculated using ordinary least squares regression. Count-based MAR was calculated using the absolute number of incident cases and deaths (Count-based MAR = MDR-TB counts / all-form TB counts × 100%). This sensitivity analysis was conducted to evaluate whether the main MAR patterns were sensitive to the use of age-standardized rates versus count-based estimates. The count-based results were broadly consistent with the

primary ASR-based analysis, suggesting that the main descriptive patterns were not solely driven by age-standardization. However, these findings should be interpreted cautiously as GBD-modeled estimates rather than direct empirical observations.

Table S17. Sensitivity analysis of the interaction coefficient (β) assessing the temporal divergence between multidrug-resistant tuberculosis (MDR-TB) and all-form tuberculosis incidence and mortality among children (0–14 years), globally and by SDI, 1990–2021, using different arbitrary log-transformation constants

Measure	Location	Constant Used	Interaction Coefficient (β)	P-value
Incidence	Global	10^{-3}	0.0381	<0.001
		10^{-6}	0.0381	<0.001
		10^{-9}	0.0381	<0.001
	High SDI	10^{-3}	-0.0012	0.803
		10^{-6}	-0.0016	0.737
		10^{-9}	-0.0016	0.737
	High-middle SDI	10^{-3}	0.0206	<0.001
		10^{-6}	0.0206	<0.001
		10-9	0.0206	<0.001
	Middle SDI	10^{-3}	0.012	0.024
		10^{-6}	0.012	0.024
		10^{-9}	0.012	0.024
	Low-middle SDI	10^{-3}	0.0772	<0.001
		10^{-6}	0.0773	<0.001
		10^{-9}	0.0773	<0.001
	Low SDI	10^{-3}	0.0574	<0.001
		10^{-6}	0.0575	<0.001
		10^{-9}	0.0575	<0.001
Deaths	Global	10^{-3}	0.0507	<0.001
		10^{-6}	0.0507	<0.001
		10^{-9}	0.0507	<0.001
	High SDI	10^{-3}	0.0308	<0.001
		10^{-6}	0.0068	0.268
		10^{-9}	0.0068	0.271
	High-middle SDI	10^{-3}	0.006	0.432
		10^{-6}	0.0045	0.563

Measure	Location	Constant Used	Interaction Coefficient (β)	P-value
		10^{-9}	0.0045	0.563
	Middle SDI	10^{-3}	0.0127	0.019
		10^{-6}	0.0124	0.022
		10^{-9}	0.0124	0.022
	Low-middle SDI	10^{-3}	0.0799	<0.001
		10^{-6}	0.0801	<0.001
		10^{-9}	0.0801	<0.001
	Low SDI	10^{-3}	0.065	<0.001
		10^{-6}	0.065	<0.001
		10^{-9}	0.065	<0.001

Note: Interaction models were specified as $\ln(\text{Rate+constant})=\beta_0+\beta_1\text{Year}+\beta_2\text{Type}+\beta_3(\text{Year}\times\text{Type})+\epsilon$. The interaction coefficient (β_3) indicates the difference in the log-linear temporal slope between pediatric MDR-TB and all-form TB. A constant was added prior to log transformation to avoid undefined values ($log(0)$) in strata with zero counts. This sensitivity analysis evaluated whether the interaction estimates were sensitive to alternative constants (10^{-3},10^{-6},and10^{-9}). The direction of the main interaction estimates was generally consistent across constants, although the mortality interaction in the high-SDI setting showed sensitivity to the chosen constant. These findings support the descriptive stability of the main interaction patterns but should not be interpreted as definitive validation of the model specification.
